# Towards the Development of a Deep Learning Framework Using Adaptive and Non-Adaptive Time-Frequency Features for EEG-Based Depression Therapy Prediction

**DOI:** 10.3390/brainsci16030301

**Published:** 2026-03-09

**Authors:** Hesam Akbari, Sara Bagherzadeh, Javid Farhadi Sedehi, Rab Nawaz, Reza Rostami, Reza Kazemi, Sadiq Muhammad, Haihua Chen, Mutlu Mete

**Affiliations:** 1 Department of Information Science, University of North Texas, Denton, TX 76205, USA; 2 Department of Biomedical Engineering, Science and Research Branch, Islamic Azad University, Tehran 1477893855, Iran; sara.bagherzadeh@srbiau.ac (S.B.); javid.farhadi@srbiau.ac.ir (J.F.S.); 3 School of Computer Science and Electronic Engineering, University of Essex, Colchester CO4 3SQ, UK; 4 Department of Psychiatry, University of Tehran, Tehran 141556619, Iran; 5 School of Computing, Gachon University, Seongnam-si 13120, Republic of Korea; 6 Department of Data Science, University of North Texas, Denton, TX 76205, USA

**Keywords:** EEG, biomedical signal processing, time-frequency analysis, deep learning, computer-aided decision

## Abstract

**Background/Objectives:** Predicting individual response to depression therapy prior to treatment initiation remains a critical clinical challenge, as the response rate to both selective serotonin reuptake inhibitors (SSRIs) and repetitive transcranial magnetic stimulation (rTMS) is approximately 50%, leaving treatment selection largely trial-based. This study presents a computer-aided decision (CAD) framework that predicts depression therapy outcomes from pre-treatment electroencephalogram (EEG) signals using advanced time-frequency representations and pretrained convolutional neural networks (CNNs). **Methods:** EEG signals from 30 SSRI patients and 46 rTMS patients are transformed into time-frequency images using Continuous Wavelet Transform (CWT), Variational Mode Decomposition (VMD), and their pixel-level fusion. Four pretrained CNN architectures, including ResNet-18, MobileNet-V3, EfficientNet-B0, and TinyViT-Hybrid, are fine-tuned and evaluated under both image-independent and subject-independent 6-fold cross-validation (CV). **Results:** Results reveal a clear therapy-specific pattern: CWT-based representations yield superior discrimination for SSRI outcome prediction, with ResNet-18 achieving 99.43% image-level accuracy, while VMD-based representations are statistically superior for rTMS outcome prediction, with ResNet-18 reaching 98.77%. Pixel-level fusion of CWT and VMD does not consistently improve performance over the best individual representation in either therapy context. Pairwise Wilcoxon signed-rank tests confirm a two-tier architectural hierarchy in which ResNet-18 and TinyViT-Hybrid significantly outperform MobileNet-V3 and EfficientNet-B0 across all conditions, while remaining statistically indistinguishable from each other. At the subject level, the framework achieves 82.50% and 83.53% accuracy for SSRI and rTMS, respectively, under strict subject-independent evaluation. Per-channel analysis reveals occipital dominance for SSRI under CWT and frontotemporal dominance for rTMS under VMD, consistent with known neurophysiological mechanisms. **Conclusions:** These findings demonstrate that the choice of time-frequency representation is therapy-specific and at least as important as architectural complexity, and that competitive performance can be achieved without recurrent or attention layers by combining well-designed spectral images with a simple pretrained residual network.

## 1. Introduction

Over the past two decades, the rate of depression disorder has been continuously increasing worldwide, particularly in developed countries [1]. Approximately 280 million people suffer from depression [2]. Depression is a serious mood disorder that causes various emotional and physical problems [3]. Patients with depression may engage in self-harm and, in some cases, suicide if they do not receive effective therapy. Indeed, suicide is the fourth most common cause of death among young people [4].

Psychiatrists typically initiate depression treatment by prescribing either selective serotonin reuptake inhibitors (SSRIs) or repetitive transcranial magnetic stimulation (rTMS) as the first course of treatment [5,6]. However, the response rate to depression therapy is approximately 50%, which means that doctors often prescribe therapies through trial and error [5]. The brain’s response to depression therapy varies from one patient to another due to the complex nature of the brain [7]. This means that a therapy that is effective for one patient may not be effective for another, and depression treatment should, therefore, be personalised. This represents a challenging task for medical teams. When a patient does not respond to one therapy, doctors switch to another; however, a failed treatment course wastes approximately two to ten weeks and increases the risk of self-harm and suicide.

Patients with depression who receive therapy are categorised into two groups: responders and non-responders. Patients who show improvement after depression therapy are categorised as responders, while those who do not respond to the therapy are categorised as non-responders [8,9,10].

Electroencephalography (EEG) is a low-cost, real-time, and widely accessible neuroimaging modality [11]. It is used in hospitals as well as local clinics to assess brain function [12]. Pre-treatment EEG recordings from patients with depression contain informative patterns that can differentiate responders from non-responders to various depression therapies [3]. However, clinicians cannot determine therapy outcomes through visual inspection of EEG signals, because EEG is nonlinear and non-stationary data with complex patterns, making it a difficult task for clinicians to decide on the most appropriate therapy to prescribe [6,13]. Developing EEG-based computer-aided decision (CAD) systems can, therefore, support clinicians in selecting the most appropriate treatment strategy.

EEG analysis has been widely investigated for predicting depression therapy outcomes. A review of prior studies shows that traditional machine learning approaches have produced limited performance. Although deep learning methods have reported improved results, most rely on conventional EEG image representations and focus on a single therapeutic modality rather than multiple depression treatments. Therefore, a robust and computationally efficient framework is still needed to predict depression therapy outcomes.

Table 1 summarises the most relevant prior studies on EEG-based prediction of depression therapy outcomes, including the methods employed, the therapy type, the number of patients, and the reported image-level classification accuracy.

To address these limitations, this study proposes generating informative time-frequency images from pre-treatment EEG signals using two advanced signal processing techniques and their fusion. Continuous Wavelet Transform (CWT) provides a multi-resolution time-frequency representation that captures the nonlinear and non-stationary nature of EEG signals [26]. CWT time-frequency representations capture oscillatory frequency patterns over time [27]. Variational Mode Decomposition (VMD), on the other hand, is an adaptive decomposition technique [28] that decomposes the EEG signal into a set of band-limited intrinsic mode functions (IMFs) with narrow frequency bands, which is more stable for oscillatory frequency components. In addition to analysing CWT and VMD-based images separately, we construct a fusion-based representation that combines information from both domains [29,30]. It has been shown that the fusion of time-frequency representations can yield richer information [29,30]. By transforming the outputs of CWT, VMD, and their fusion into structured time-frequency images, the information in EEG related to treatment response can be more effectively utilised.

In addition, we investigate the use of four well-known pretrained convolutional neural network (CNN) architectures: ResNet-18 [31], MobileNet-V3 [32,33], EfficientNet-B0 [34,35], and TinyViT-Hybrid [36,37,38] for classifying patients into responder (R) and non-responder (NR) groups based on the CWT, VMD, and fusion-derived EEG images. ResNet-18 is a lightweight residual model that facilitates deeper training through skip connections, while MobileNet-V3 uses depthwise convolutions and attention modules for high efficiency [31,32,33]. EfficientNet-B0 applies compound scaling to balance network width, depth, and resolution to achieve better performance [34,35]. TinyViT-Hybrid integrates residual convolutional blocks with a compact vision transformer to capture both local and global representations in a low-complexity model [36,37,38].

By comparing these architectures across three types of input representations (i.e., CWT, VMD, and their fusion), we obtain a comprehensive view of the trade-offs between accuracy and robustness for EEG-based prediction of depression therapy outcomes. The aim of this work is to develop and evaluate a robust EEG-based CAD system that integrates advanced time-frequency analysis with state-of-the-art pretrained CNN models to predict treatment response for SSRI and rTMS depression therapies.

The remainder of this paper is organised as follows. Section 2 describes the two EEG datasets used in this study. Section 3 covers the preprocessing steps applied to the EEG data. Section 4 presents the methodology, including the time-frequency representations, the pretrained CNN models, and the statistical evaluation metrics. Section 5 reports and discusses the experimental results. Section 6 presents the conclusion of the study.

## 2. Materials

**Figure 1:**
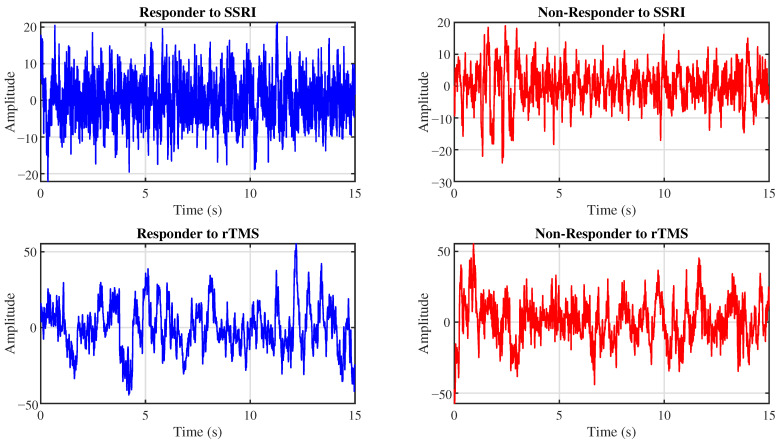
Example EEG signals from the Fp1 channel for R (**left**) and NR (**right**) patients to SSRI therapy (**top row**) and rTMS therapy (**bottom row**).

The proposed CAD system is evaluated using two EEG datasets. This section details these datasets. Figure 1 presents representative EEG signals for R and NR patients from the SSRI and rTMS datasets.  

### 2.1. SSRI Dataset

The SSRI dataset from Universiti Teknologi PETRONAS was used to evaluate the models in predicting treatment response [14]. It contains five-minute pre-treatment EEG recordings from 30 patients diagnosed with depression. SSRI therapy was prescribed for all patients in this cohort. EEG signals were recorded in an eyes-closed resting-state condition using the 10–20 international electrode placement system. The sampling frequency was 256 Hz and the EEG data comprised 19 channels.

Depression severity was assessed before and after a six-week therapy course using the Beck Depression Inventory (BDI). Patients showing more than a 50% reduction in their BDI scores were labelled as responders, while those with 50% or less improvement were labelled as non-responders. The Universiti Teknologi Malaysia Research Ethics Committee approved the data recording protocol [14]. The dataset contains 12 EEG recordings from R and 18 recordings from NR patients to SSRI therapy, provided in EDF format.

### 2.2. rTMS Dataset

The rTMS dataset was collected at Atieh Hospital, Tehran, Iran, and contains pre-treatment EEG recordings from 46 patients diagnosed with depression. Five-minute resting-state EEG signals were recorded for each patient with eyes closed, using the 10–20 international electrode placement system at a sampling frequency of 500 Hz and 19 channels.

All patients underwent a six-week rTMS therapy course. Depression severity was assessed before and after treatment using the BDI. Patients showing more than 50% improvement in BDI scores were labelled as responders, while those with 50% or less improvement were labelled as non-responders. The dataset contains EEG recordings from 23 responders and 23 non-responders.

The Ethics Committee of Shahid Beheshti University of Medical Sciences approved the data collection protocol [7], and the research team obtained written informed consent from all participants prior to data collection [7,23,24].

## 3. Preprocessing

EEG signals were band-pass filtered within 0.5–70 Hz to attenuate slow baseline fluctuations and high-frequency muscle contamination while preserving physiologically meaningful activity for brain rhythms. A 50 Hz notch filter was used to suppress power-line noise. Motion-related artifacts were mitigated using the EEGLAB toolbox version 4.5b [39]. All filtering operations were performed using zero-phase forward and reverse filtering via MATLAB R-2023a’s filtfilt function to prevent phase distortion. The processed EEG recordings were subsequently re-referenced to the common average reference (CAR) to reduce inter-channel reference bias. Finally, each subject’s data were divided into non-overlapping 15-s segments.

The continuous recordings were segmented into non-overlapping 15-s epochs. This window length was selected to achieve a balance between spectral resolution and statistical robustness. Shorter windows below 10 s resulted in unreliable estimation of low-frequency resolution, whereas windows exceeding 20 s reduced the total number of images. Therefore, 15 s represented a practical compromise between frequency resolution and sample size. High-amplitude disturbances related to movement or environmental interference were attenuated using the artifact subspace reconstruction (ASR) function in EEGLAB toolbox.

Each 15-s EEG was denoised using Multiscale Principal Component Analysis (MSPCA). MSPCA is a hybrid signal processing approach that combines wavelet decomposition and principal component analysis (PCA) to remove noise across multiple temporal scales. In MSPCA, the EEG signal is first decomposed into multiresolution wavelet subbands, and PCA is then applied within each scale to separate structured neural activity from noise components. Reconstruction is performed after suppressing noise-dominated components, resulting in enhanced signal quality while preserving physiologically meaningful brain rhythms.

The denoising procedure was implemented in MATLAB using the wmulden function with a decomposition level of 9 and the Symlet wavelet of order 4. Soft thresholding was applied based on the universal threshold selection rule, and the number of principal components retained at both the approximation and final reconstruction stages was determined using Kaiser’s criterion. These parameter settings were selected to provide effective multiscale noise reduction while maintaining clinically relevant EEG information. The number of images generated per subject for the SSRI and rTMS datasets after preprocessing is presented in Table 2.

## 4. Methodology

**Figure 2:**
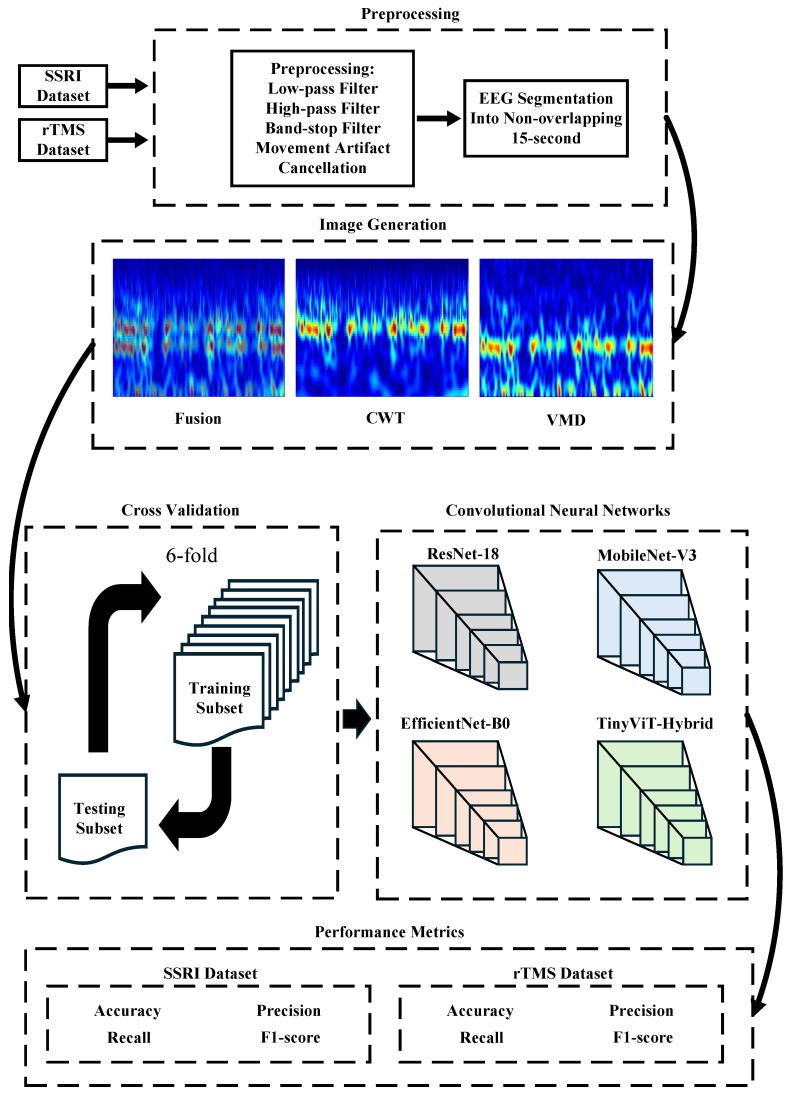
Workflow of the proposed computer-aided decision (CAD) system for predicting depression therapy outcomes from pre-treatment EEG signals. The pipeline comprises five stages: (1) Preprocessing: raw EEG recordings from the SSRI and rTMS datasets are band-pass filtered (0.5–70 Hz), notch filtered (50 Hz), motion-artifact corrected via artifact subspace reconstruction (ASR), re-referenced to the common average reference (CAR), denoised using Multiscale Principal Component Analysis (MSPCA), and segmented into non-overlapping 15-s epochs; (2) Image Generation: each epoch and channel (19 total) is transformed into a time-frequency image using Continuous Wavelet Transform (CWT), Variational Mode Decomposition (VMD), or their pixel-wise average (Fusion); (3) Cross-Validation: 6-fold CV applied at either the image level (image-independent) or patient level (subject-independent); (4) Classification: four ImageNet-pretrained CNNs (ResNet-18, MobileNet-V3, EfficientNet-B0, TinyViT-Hybrid) are fine-tuned to classify images as responder (R) or non-responder (NR); (5) Performance Metrics: accuracy, precision, recall, specificity, and F1-score are reported at the image level; subject-level accuracy with 95% CI is reported under subject-independent CV.

This section describes the steps of the CAD system for predicting the outcomes of depression therapies. Figure 2 provides an overview of the entire pipeline.

### 4.1. Time-Frequency Image Generation

To extract informative spectral-temporal patterns from pre-treatment EEG signals, three types of time-frequency representations are generated: CWT-based images, VMD-based images, and a fused representation combining both domains. This section describes the image generation techniques.

#### 4.1.1. Continuous Wavelet Transform

Continuous Wavelet Transform (CWT) is used to generate high-resolution time-frequency representations that capture the nonlinear and non-stationary characteristics of EEG data [26]. The CWT provides a multi-resolution time-frequency decomposition of a signal by correlating it with scaled and translated versions of a mother wavelet [27,40]. For a continuous signal x(t), the CWT is defined as:

(1) $$W\left(a,b\right)=\frac{1}{\sqrt{a}}\int_{-\infty }^{+\infty }x\left(t\right)\;\psi^{*}\;\left(\frac{t-b}{a}\right)dt$$

where a>0 is the scale parameter, b∈R is the translation parameter, ψ(t) is the mother wavelet, and ${\left(\cdot \right)}^{*}$ denotes complex conjugation. The scale *a* is inversely related to frequency *f* through $f=f_{c}/\left(a\cdot \Delta t\right)$, where $f_{c}$ is the centre frequency of the wavelet and Δt is the sampling interval.

The analytic Morlet wavelet was selected as the mother wavelet due to its optimal joint time-frequency localization [26]. The Morlet wavelet is defined as:

(2) $$\psi \left(t\right)=\pi^{-1/4}\;e^{j\omega_{0}t}\;e^{-t^{2}/2}$$

where $\omega_{0}$ is the central angular frequency. The analytic form of the Morlet wavelet suppresses negative frequency components, yielding a one-sided spectrum that is more suitable for interpreting oscillatory neural activity in EEG signals. The scalogram, representing the time-frequency energy distribution, is obtained from the squared modulus of the CWT coefficients:

(3) $$S\left(a,b\right)={\left|W(a,b)\right|}^{2}$$

This representation captures how the spectral energy of the EEG signal evolves over time, enabling visualization of non-stationary oscillatory dynamics associated with different frequency bands.

**Figure 3:**
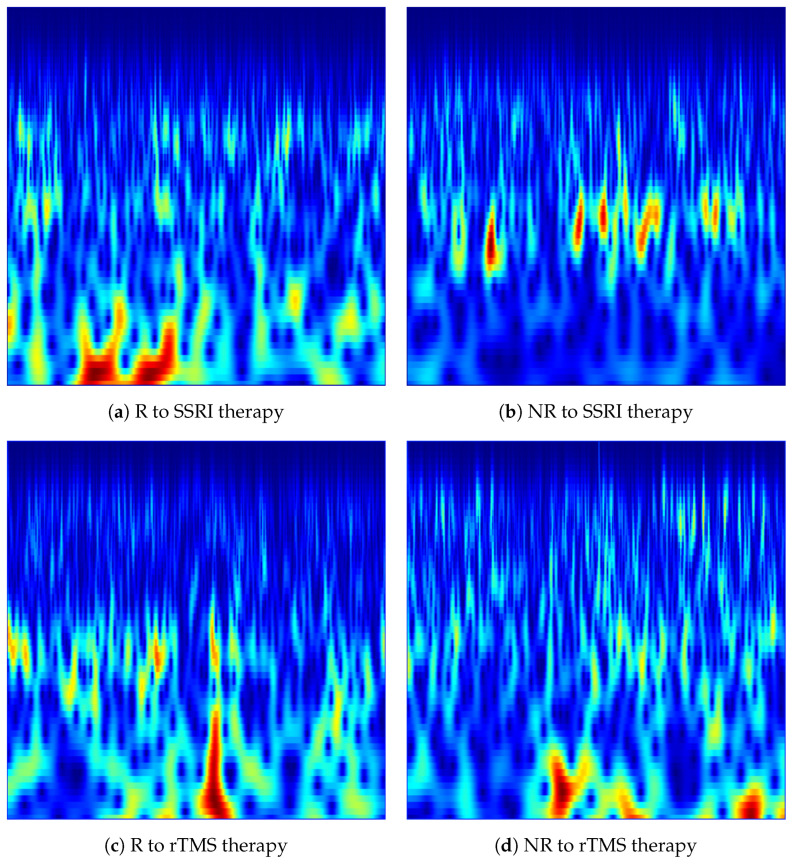
CWT-based time-frequency images generated from the Fp1 channel for responder (R) and non-responder (NR) patients across SSRI and rTMS therapies. The images illustrate the spectral-temporal structure of EEG activity used for predicting treatment outcomes.

In the implementation, each denoised 15-s EEG segment was transformed into a scalogram image independently for each of the 19 channels. The CWT was computed using MATLAB’s cwt function with the analytic Morlet wavelet (‘amor’), with 12 voices per octave to provide fine-grained frequency resolution across scales. The frequency range was restricted to [2, 60] Hz to cover all clinically relevant EEG rhythms, namely delta (δ: 1–4 Hz), theta (θ: 4–8 Hz), alpha (α: 8–13 Hz), beta (β: 13–30 Hz), and low gamma (γ: 30–60 Hz), while excluding DC drift and high-frequency noise components. The magnitude of the CWT coefficients |W(a,b)| was used as the pixel intensity in each scalogram, and the jet colormap was applied to map amplitude values to a perceptually distinguishable colour space. Each scalogram was rendered as a borderless image at a resolution of 200 DPI and saved in PNG format. The resulting CWT images constitute a structured two-dimensional representation of each EEG, with the horizontal axis encoding time and the vertical axis encoding frequency, suitable for input to pretrained convolutional neural networks. Figure 3 shows representative CWT scalogram images generated from the Fp1 channel for R and NR subjects from each dataset.

#### 4.1.2. Variational Mode Decomposition

Variational Mode Decomposition (VMD) is an adaptive, non-recursive signal decomposition method that overcomes the mode-mixing and boundary-effect limitations of Empirical Mode Decomposition [28,41]. VMD simultaneously decomposes a real-valued signal x(t) into *K* band-limited intrinsic mode functions (IMFs), denoted ${\left\{u_{k}\left(t\right)\right\}}_{k=1}^{K}$, each centred around a distinct centre frequency ${\left\{\omega_{k}\right\}}_{k=1}^{K}$. The decomposition is formulated as a constrained variational optimisation problem that minimises the total bandwidth of all modes subject to their sum reconstructing the original signal [42].

For each mode $u_{k}\left(t\right)$, its analytic signal is computed via the Hilbert transform, and the spectrum is frequency-shifted to baseband by modulating with $e^{-j\omega_{k}t}$ [43]. The constrained optimisation problem is defined as:

(4) $$\min_{\left\{u_{k}\right\},\left\{\omega_{k}\right\}}\left\{\sum_{k=1}^{K}{∥\partial_{t}\left[\left(\delta \left(t\right)+\frac{j}{\pi t}\right)*u_{k}\left(t\right)\right]e^{-j\omega_{k}t}∥}_{2}^{2}\right\}\;s.t.\;\sum_{k=1}^{K}u_{k}\left(t\right)=x\left(t\right)$$

where δ(t) is the Dirac delta, ∗ denotes convolution, and $\partial_{t}$ is the partial derivative with respect to time. The $l_{2}$-norm of the time-derivative of the analytic baseband signal serves as a proxy for the instantaneous bandwidth of each mode.

To solve Equation (4), the constrained problem is converted into an unconstrained saddle-point problem using an augmented Lagrangian:

(5) $$L\;\left(\left\{u_{k}\right\},\left\{\omega_{k}\right\},\lambda \right)=\alpha \sum_{k=1}^{K}{∥\partial_{t}\left[\left(\delta +\frac{j}{\pi t}\right)*u_{k}\right]e^{-j\omega_{k}t}∥}_{2}^{2}+{∥x-\sum_{k=1}^{K}u_{k}∥}_{2}^{2}+\langle \lambda ,\;x-\sum_{k=1}^{K}u_{k}\rangle$$

where α is a quadratic penalty parameter controlling mode bandwidth, and λ(t) is the Lagrange multiplier enforcing the reconstruction constraint. The modes, centre frequencies, and multipliers are iteratively updated in the Fourier domain until convergence. The spectral update rule for each mode is:

(6) $${\hat{u}}_{k}^{n+1}\left(\omega \right)=\frac{\hat{x}\left(\omega \right)-\sum_{i\neq k}{\hat{u}}_{i}\left(\omega \right)+\frac{\hat{\lambda }\left(\omega \right)}{2}}{1+2\alpha {\left(\omega -\omega_{k}\right)}^{2}}$$

and the centre frequency is updated as:

(7) $$\omega_{k}^{n+1}=\frac{\int_{0}^{\infty }\omega {\left|{\hat{u}}_{k}\left(\omega \right)\right|}^{2}d\omega }{\int_{0}^{\infty }{\left|{\hat{u}}_{k}\left(\omega \right)\right|}^{2}d\omega }$$

where ${\hat{u}}_{k}\left(\omega \right)$ and $\hat{x}\left(\omega \right)$ denote the Fourier transforms of $u_{k}\left(t\right)$ and x(t), respectively. Equation (7) computes the centre of gravity of the power spectrum of the *k*-th mode, which drives each mode towards its dominant frequency bin. The Lagrange multiplier is updated as:

(8) $${\hat{\lambda }}^{n+1}\left(\omega \right)={\hat{\lambda }}^{n}\left(\omega \right)+\tau \left(\hat{x}\left(\omega \right)-\sum_{k=1}^{K}{\hat{u}}_{k}^{n+1}\left(\omega \right)\right)$$

where τ is the dual ascent step size. Iterations continue until the convergence criterion $\sum_{k}∥{\hat{u}}_{k}^{n+1}-{\hat{u}}_{k}^{n}{∥}_{2}^{2}/{∥{\hat{u}}_{k}^{n}∥}_{2}^{2}<\varepsilon$ is satisfied.

Unlike CWT, which directly produces a time-frequency map, VMD yields a set of narrowband temporal waveforms. To obtain a structured two-dimensional time-frequency representation compatible with CNN input, the Short-Time Fourier Transform (STFT) spectrogram was computed for each IMF and the resulting complex spectrograms were accumulated across all modes [44]. For a given IMF $u_{k}\left(t\right)$, the STFT is defined as:

(9) $$S_{k}\left(t,f\right)=\int_{-\infty }^{+\infty }u_{k}\left(\tau \right)\;g\left(\tau -t\right)\;e^{-j2\pi f\tau }\;d\tau$$

where g(τ−t) is a sliding analysis window centred at time *t*. The aggregate complex spectrogram across all *K* modes is then:

(10) $$S_{\mathrm{VMD}}\left(t,f\right)=\sum_{k=1}^{K}S_{k}\left(t,f\right)$$

and the magnitude $\left|S_{\mathrm{VMD}}\left(t,f\right)\right|$ was rendered as a two-dimensional image. This summation strategy integrates the spectral contributions of each narrow-band mode, thereby recovering a full time-frequency picture of the original EEG epoch while preserving the sharp frequency localisation achieved by VMD decomposition.

**Figure 4:**
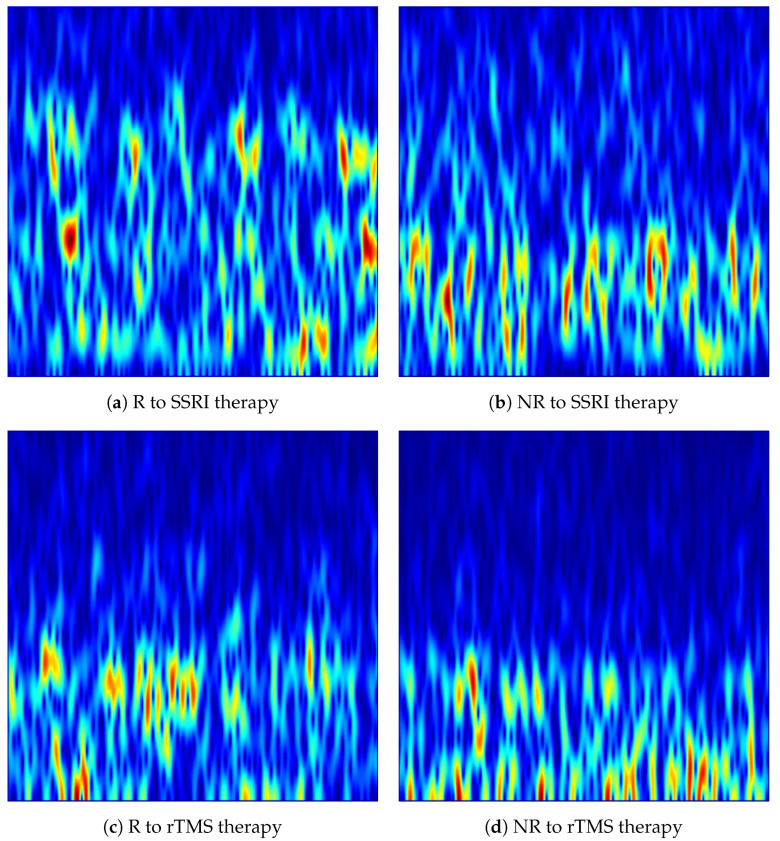
VMD-based time-frequency images generated from the Fp1 channel for responder (R) and non-responder (NR) patients across SSRI and rTMS therapies. The images highlight intrinsic mode variations that differentiate treatment-responsive from treatment-resistant neural activity.

In the implementation, each denoised 15-s EEG was processed independently for each of the 19 channels. MATLAB’s vmd function was applied with K=20 IMFs, decomposing each single-channel epoch into 20 band-limited components (i.e., IMFs). The STFT spectrogram of each IMF was subsequently computed using MATLAB’s spectrogram function with a Hamming window of length 256 samples, an overlap of 250 samples (step size of 6 samples), and 7680 FFT points at the resampled rate of $f_{s}=512$ Hz. The high overlap ensured dense temporal sampling of the spectrogram to preserve fine-grained temporal dynamics within each epoch. The 20 complex spectrograms were accumulated as in Equation (10), and the magnitude of the summed spectrogram was visualised. The frequency axis was restricted to [0, 60] Hz to focus on the clinically relevant delta, theta, alpha, beta and gamma EEG rhythms, consistent with the spectral range of interest for depression biomarker analysis. The jet colormap was applied to map magnitude values to colour, axes and tick labels were suppressed, and each image was saved in PNG format. The resulting VMD-based images constitute structured time-frequency representations suitable for direct input to pretrained CNNs. Figure 4 shows representative VMD-based spectrogram images generated from the Fp1 channel for R and NR subjects from each dataset.

#### 4.1.3. Fusion of CWT and VMD Representations

CWT and VMD capture complementary aspects of EEG spectral-temporal dynamics. CWT provides a continuous, multi-resolution time-frequency representation that tracks non-stationary oscillatory activity across a broad frequency range, whereas VMD yields a set of narrowband, adaptively decomposed modes whose accumulated spectrogram reflects stable rhythmic components with fine frequency localisation. Since the two representations encode different but mutually reinforcing aspects of the same underlying neural signal, combining them into a single fused image is expected to improve the feature space available to the downstream CNN classifier [29,30].

Let $I_{\mathrm{CWT}}\in R^{H\times W\times C}$ and $I_{\mathrm{VMD}}\in R^{H\times W\times C}$ denote the CWT scalogram image and the VMD-based spectrogram image, respectively, both corresponding to the same EEG signal, channel, and subject. Prior to fusion, each image is independently normalised to the unit interval using min-max normalisation:

(11) $$\tilde{I}=\frac{I-\min(I)}{\max(I)-\min(I)}$$

where min(·) and max(·) are computed over all pixel values of the individual image. This per-image normalisation ensures that neither representation dominates the fused output due to differences in absolute amplitude scale arising from the distinct computational pathways of CWT and VMD.

The fused image $I_{\mathrm{fused}}$ is obtained by pixel-wise arithmetic averaging of the two normalised representations:

(12) $$I_{\mathrm{fused}}\left(i,j\right)=\frac{{\tilde{I}}_{\mathrm{CWT}}\left(i,j\right)+{\tilde{I}}_{\mathrm{VMD}}\left(i,j\right)}{2}$$

where (i,j) indexes a spatial pixel location. The averaging operation in Equation (12) preserves the dynamic range within [0,1], avoids saturation artefacts, and assigns equal weight to each representation. At each pixel, the fused value reflects the joint contribution of the instantaneous wavelet energy at that time-frequency coordinate from the CWT and the accumulated narrowband spectral energy from the VMD-derived spectrogram. Regions of the time-frequency plane that are consistently active in both representations are reinforced in the fused image, while modality-specific transient features are attenuated, yielding a more robust composite representation.

From an information-theoretic perspective, the pixel-wise average can be understood as a linear combination of two complementary feature maps. Because CWT and VMD decompose the same signal through non-adaptive and adaptive mechanisms respectively, their representations are not identical, and the fusion can capture structured patterns that neither representation alone encodes completely. This complementarity has been shown to improve classification performance in multimodal signal analysis tasks [29,30].

**Figure 5:**
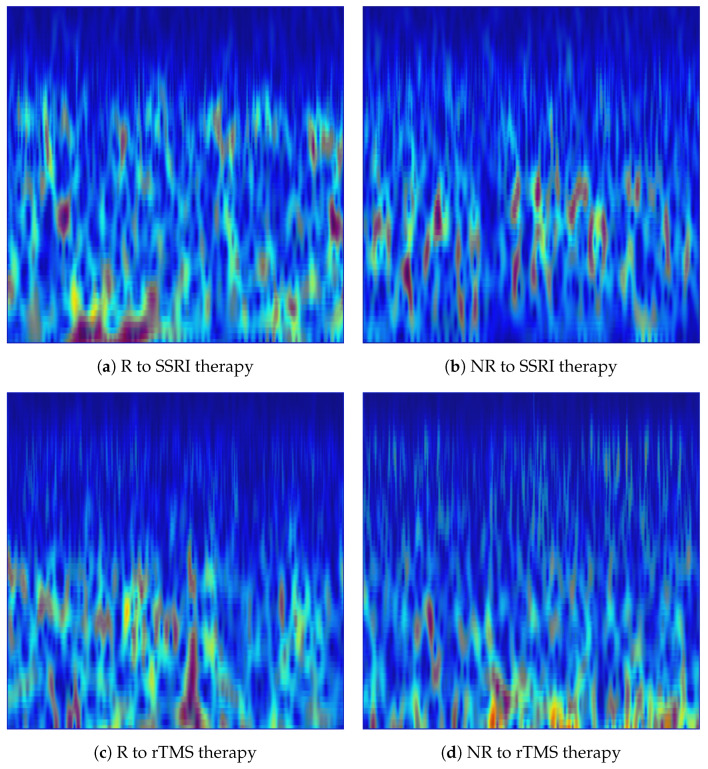
Fusion-based EEG representations of responder (R) and non-responder (NR) across SSRI and rTMS therapies, generated from the Fp1 channel. The fused time-frequency patterns combine the information of CWT scalograms and VMD-derived spectrograms via pixel-wise averaging to emphasise discriminative structures for treatment-outcome prediction.

In the implementation, each CWT image and its corresponding VMD image, matched by subject, segment index, and channel identifier embedded in the filename, were loaded. Both images were converted to double-precision floating-point arrays using im2double and subsequently normalised to [0,1] using mat2gray, which implements the min-max operation defined in Equation (11). The normalised images were then averaged pixel-wise as in Equation (12), and the resulting fused image was saved in PNG format using imwrite. The same procedure was applied independently to all 19 EEG channels across all subjects and segments in both the SSRI and rTMS datasets. The resulting fused images share the same spatial dimensions and filename convention as the individual CWT and VMD images, and serve as a third distinct input modality for the pretrained CNN classifiers evaluated in this study. Figure 5 shows representative fused images for R and NR subjects from each dataset. Table 3 summarises the complete set of parameters used for CWT, VMD, and Fusion image generation.

### 4.2. Pretrained CNN Models

Four pretrained convolutional neural network architectures are evaluated as classifiers in the proposed CAD system. All models are initialised with ImageNet pretrained weights and fine-tuned end-to-end on the EEG time-frequency images. The final classification layer of each model is replaced with a new linear layer with two output neurons corresponding to the R and NR classes.

ResNet-18 is an 18-layer residual network in which skip connections enable gradient flow across deeper layers, mitigating the vanishing gradient problem [31]. The original fully connected layer with 1000 outputs is replaced by a linear layer mapping the 512-dimensional feature vector to the two-class output space.

MobileNet-V3 Large is a lightweight architecture that combines inverted residual blocks with hard-swish activations and a Squeeze-and-Excitation attention mechanism, designed for efficient inference [32,33]. The last linear layer of its three-layer classifier head is replaced with a binary output layer while the preceding adaptive average pooling and the first two classifier layers are retained.

EfficientNet-B0 is the baseline model of the EfficientNet family, which applies compound scaling to jointly increase network depth, width, and input resolution [34,35]. Its final classification layer is replaced with a two-output linear layer while the preceding dropout and the feature extraction backbone remain intact.

TinyViT-Hybrid is a hybrid architecture proposed in this work that couples a convolutional feature extractor with a compact Transformer encoder [36,37,38]. The ResNet-18 backbone is retained up to and including layer4, producing spatial feature maps of shape (B,512,7,7) for 224×224 inputs. These feature maps are reshaped into a sequence of 49 non-overlapping tokens of dimension 512, each corresponding to one spatial position in the 7×7 grid. Learnable 1-D positional embeddings are added to each token to inject spatial order information. The token sequence is then passed through a Transformer encoder consisting of two Multi-Head Self-Attention layers with H=8 attention heads, a feed-forward inner dimension of 4×512=2048 (MLP ratio of 4), and a dropout rate of 0.1. The self-attention mechanism allows each spatial token to attend to all other tokens, capturing global spatial dependencies across the feature map. The attended token sequence is normalised with Layer Normalisation, and the 512-dimensional mean-pooled representation is passed to a linear classification head. The Transformer block is implemented using timm’s Vision Transformer Block.

Detailed architectural diagrams of the four pretrained convolutional neural network models evaluated in this study, namely ResNet-18 (Figure S1), MobileNet-V3 (Figure S2), EfficientNet-B0 (Figure S3), and TinyViT-Hybrid (Figure S4), accompanied by descriptive text explaining the layer structure, spatial resolution evolution, and task-specific modifications applied to each model (Sections S1–S4).

All experiments were implemented in Python 3.14.3 using PyTorch 2.10.0. Each model was initialised with ImageNet pretrained weights provided by torchvision and fine-tuned end-to-end on the EEG time-frequency images. Training was performed using the AdamW optimiser with a learning rate of $1\times 10^{-4}$ and a weight decay of $1\times 10^{-4}$. A cosine annealing learning rate scheduler was applied over $T_{\max}=8$ epochs with a minimum learning rate of $1\times 10^{-6}$. The maximum number of training epochs per fold was set to 8, with early stopping applied on the validation loss with a patience of 3 epochs to prevent overfitting. The batch size was set to 32 for all models and experiments, and the cross-entropy loss was used as the training objective for all four architectures. All input images were resized to 224×224 pixels and normalised using ImageNet statistics. No augmentation was applied during training and testing. A fixed random seed of 42 was used to ensure reproducibility across all runs, with the seed incremented by 100× fold index at the start of each fold to diversify weight initialisation across folds. A summary of the architecture-specific and shared training hyperparameters is provided in Table 4.

### 4.3. Dataset Statistics and Validation Strategy

The CAD system is evaluated on the SSRI and rTMS datasets. In each dataset, EEG recordings are available for both R and NR patients. Regardless of class, all EEG signals are segmented into non-overlapping 15-s segments, and a time-frequency image is generated for each segment (each segment has 19 channels). Table 5 provides the total number of R and NR EEG images generated from the SSRI and rTMS datasets. The table shows that the number of images is large enough to support training of the pretrained CNN models. However, in the SSRI dataset the number of NR images exceeds that of R images, which is a direct consequence of the larger number of NR patients in the dataset.

The test data can be categorised into four situations based on the predictions of the CAD system. A true positive (TP) occurs when a R image is correctly predicted as R by the model. A true negative (TN) occurs when a NR image is correctly predicted as NR. A false positive (FP) occurs when a NR image is incorrectly predicted as R. A false negative (FN) occurs when a R image is incorrectly predicted as NR. The performance of each CAD system was quantified using five standard evaluation metrics: accuracy, precision, recall, specificity, and F1-score. Accuracy reflects the overall correctness of the classifier and is defined as:

(13) $$\mathrm{Accuracy}=\frac{\mathrm{TP}\;+\;\mathrm{TN}}{\mathrm{TP}\;+\;\mathrm{TN}\;+\;\mathrm{FP}\;+\;\mathrm{FN}}\times 100$$

Precision reflects the model’s confidence in its R predictions by measuring the proportion of predicted R samples that are truly responders:

(14) $$\mathrm{Precision}=\frac{\mathrm{TP}}{\mathrm{TP}\;+\;\mathrm{FP}}\times 100$$

Recall quantifies how accurately the model detects R samples:

(15) $$\mathrm{Recall}=\frac{\mathrm{TP}}{\mathrm{TP}\;+\;\mathrm{FN}}\times 100$$

Specificity measures how effectively the model identifies non-R samples:

(16) $$\mathrm{Specificity}=\frac{\mathrm{TN}}{\mathrm{TN}\;+\;\mathrm{FP}}\times 100$$

The F1-score combines precision and recall into a single balanced measure:

(17) $$F1=\frac{2\times \mathrm{Precision}\times \mathrm{Recall}}{\mathrm{Precision}+\mathrm{Recall}}\times 100$$

To minimise result bias, each model is evaluated under two complementary *K*-fold cross-validation (CV) strategies. The first is an image-independent 6-fold CV, in which all images from both classes are pooled and partitioned into six folds using stratified sampling to preserve the class ratio in each fold. Within each fold, 90% of the non-test images form the training set and the remaining 10% serve as the validation set. This strategy evaluates how well the model distinguishes R from NR images when training and test images may originate from the same subject and, therefore, provides an upper-bound estimate of image-level discrimination performance. Performance is assessed at the image level by comparing the predicted class label of every test image against its true label to derive a fold-level confusion matrix.

The second strategy is a subject-independent 6-fold CV, in which the data are partitioned at the subject level so that all images from a given subject appear exclusively in either the training or the test set within each fold, but never in both. For the SSRI dataset, which contains 12 responders and 18 non-responders, each test fold consists of 2 R and 3 NR, ensuring that every subject appears in the test set exactly once across the six folds. For the rTMS dataset, which contains 23 R and 23 NR, the first five test folds each contain 4 R and 4 NR, while the sixth fold contains 3 R and 3 NR to account for the remainder. In both cases, the remaining training subjects are further split into a 90% training subset and a 10% stratified validation subset. Subject-independent CV provides a more clinically realistic estimate of generalisation, as it simulates prediction for a previously unseen patient. In the subject-level CV strategy, a subject is considered correctly classified when the majority of the subject’s images are correctly classified. Table 6 summarises the configuration and purpose of the two CV strategies employed in this study.

In image-level strategies, accuracy, precision, recall, specificity, and F1-score are computed from the fold-level confusion matrix and reported as the mean and standard deviation across the six folds. For the subject-independent strategy, subject-level accuracy along with a 95% confidence interval (CI) is reported. Additionally, a per-channel accuracy analysis is performed in the subject-level CV strategy.

## 5. Results and Discussion

This section reports and discusses the experimental results. Two independent datasets are used to evaluate the proposed CAD system for predicting the outcomes of SSRI and rTMS therapies, as described in Section 2. In the first step of the proposed pipeline, the EEG signals are denoised following the steps described in Section 3 and segmented into non-overlapping 15-s windows. The time-frequency methods are then applied to the EEG signals to convert them into images. The EEG images are generated using CWT, VMD, and their fusion as described in Section 4.1. The resulting images are subsequently fed into four pretrained CNN architectures with fine-tuned classification layers, whose structures are described in Section 4.2.

### 5.1. Performance of CAD System for SSRI Therapy

**Figure 6:**
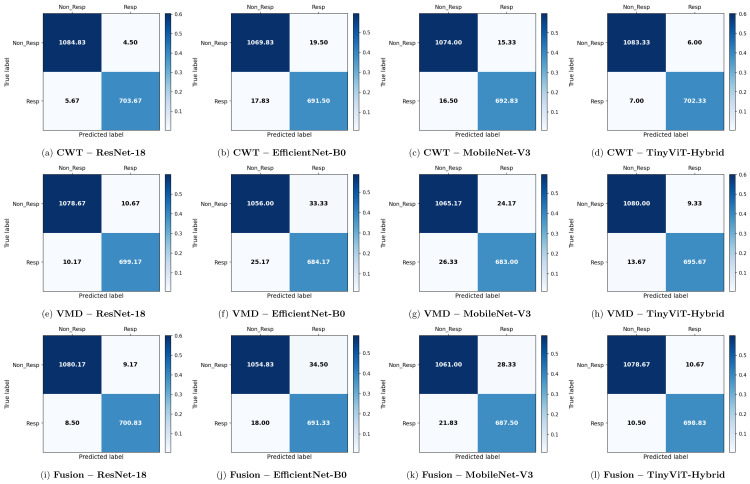
Average 6-fold confusion matrices for SSRI treatment response prediction under image-independent CV. Results are shown for three time-frequency representations—CWT (panels **a**–**d**), VMD (panels **e**–**h**), and Fusion (panels **i**–**l**)—and four CNN architectures (ResNet-18, EfficientNet-B0, MobileNet-V3, TinyViT-Hybrid). Each cell reports the mean count averaged across the six folds; non-integer values arise from this averaging. Rows indicate the true class label and columns the predicted label, where Resp = responder and Non_Resp = non-responder to SSRI therapy. The colour scale reflects the normalised proportion per cell. The approximate number of test images per fold is 2159 (∼709R and ∼1090NR).

**Figure 7:**
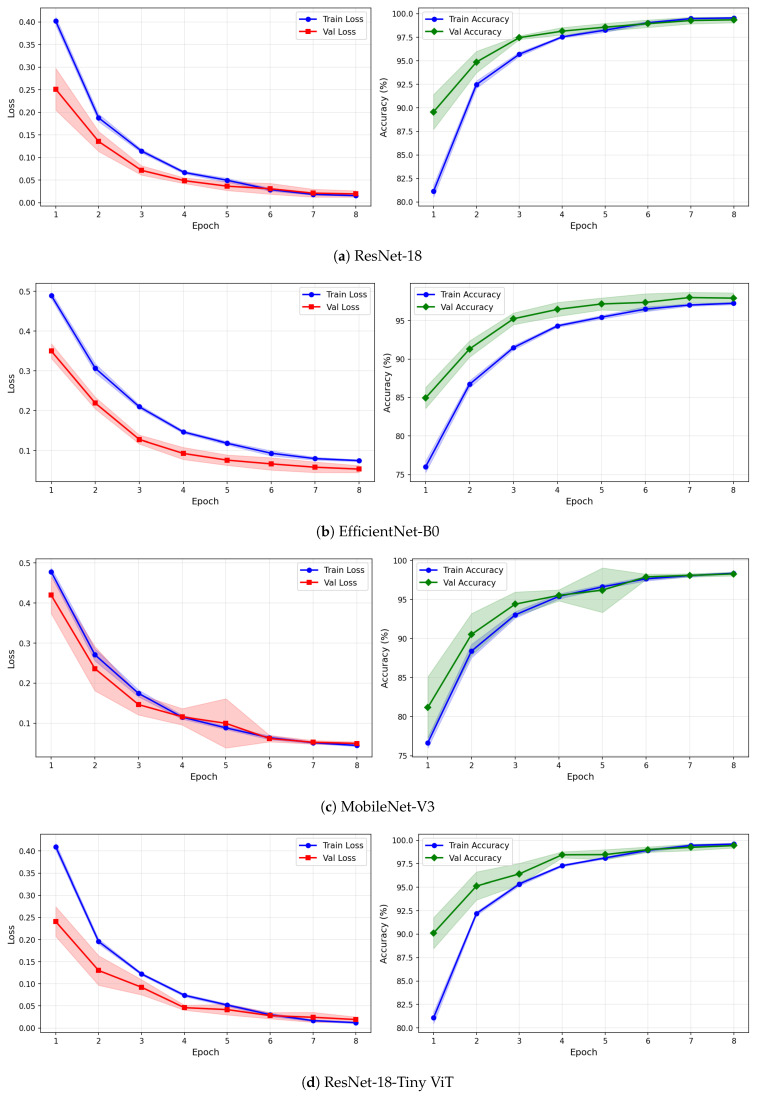
Training and validation loss and accuracy curves for CWT-based representations on the SSRI dataset under image-independent CV. Results are averaged across six folds with standard deviation shading.

**Figure 8:**
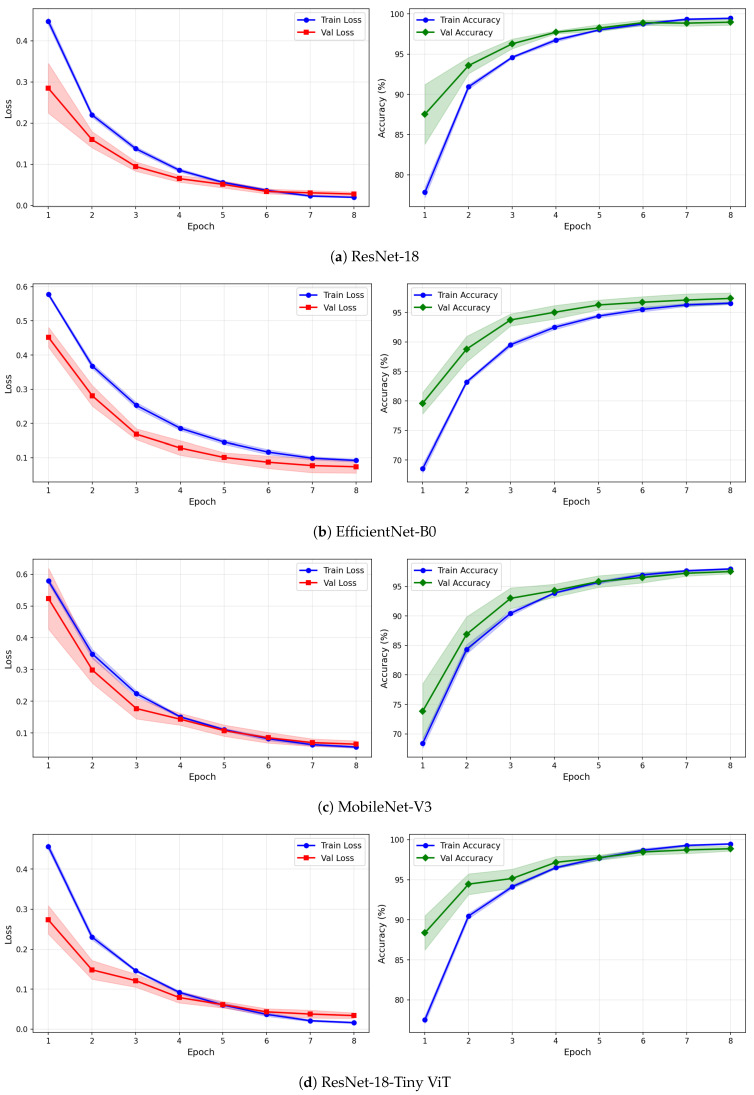
Training and validation loss and accuracy curves for VMD-based representations on the SSRI dataset under image-independent CV. Results are averaged across six folds with standard deviation shading.

**Figure 9:**
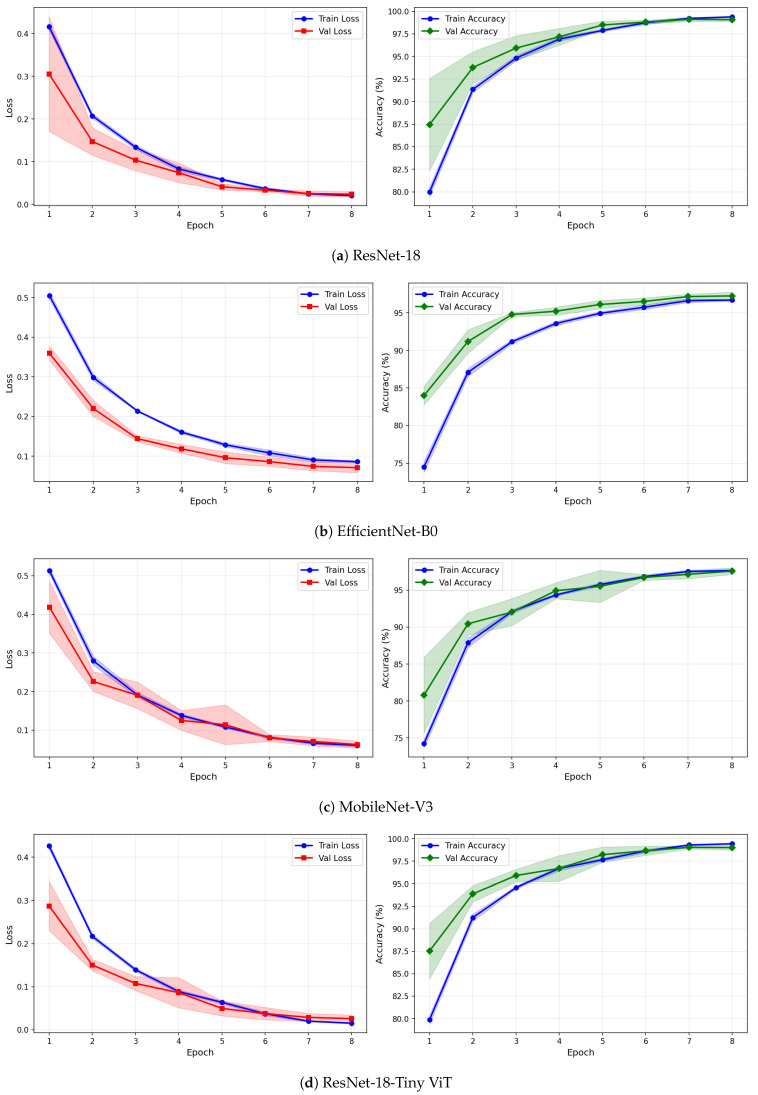
Training and validation loss and accuracy curves for the Fusion (CWT+VMD) representation on the SSRI dataset under image-independent CV. Results are averaged across six folds with standard deviation shading.

**Figure 10:**
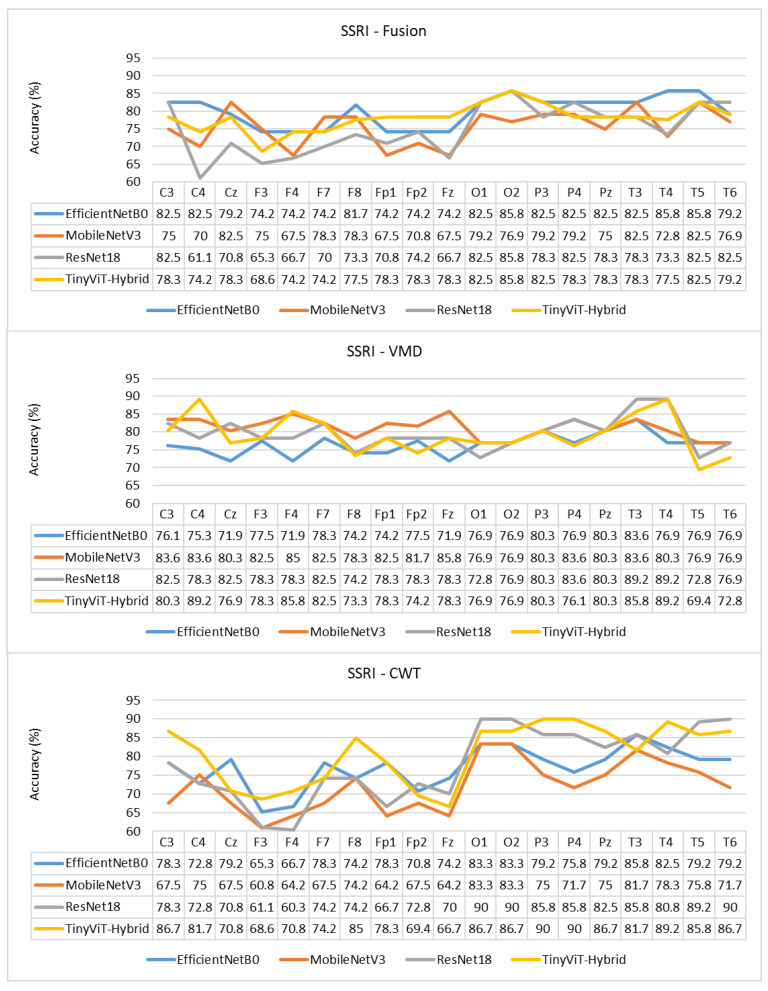
Per-channel subject-level accuracy (%) for the SSRI dataset under subject-independent CV across CWT, VMD, and Fusion representations and four CNN models.

Table 7 reports the image-level classification performance of the four pretrained CNN models under image-independent 6-fold CV for the SSRI dataset across all three time-frequency representations. Table 8 reports the corresponding subject-level accuracy under subject-independent CV. The average confusion matrices for all twelve model–representation combinations are visualised in Figure 6. The training and validation dynamics are presented in Figure 7, Figure 8 and Figure 9 for CWT, VMD, and Fusion representations respectively. The per-channel subject-level accuracy under subject-independent CV is shown in Figure 10.

Across all representations, ResNet-18 consistently achieves the highest image-level performance. With CWT-based images, ResNet-18 attains the best overall accuracy of 99.43% ± 0.14%, a precision of 99.37% ± 0.43%, a recall of 99.20% ± 0.37%, a specificity of 99.59% ± 0.28%, and an F1-score of 99.28% ± 0.18%. The corresponding confusion matrix confirms this result, showing mean per-fold misclassification counts of only 4.50 false positives and 5.67 false negatives out of approximately 1798 test images per fold. The TinyViT-Hybrid hybrid achieves comparable performance with CWT images, reaching an accuracy of 99.28% ± 0.16% and an F1-score of 99.08% ± 0.21%, indicating that the integration of local convolutional features with global Transformer-based attention does not substantially compromise accuracy relative to the plain residual architecture on this dataset.

MobileNet-V3 and EfficientNet-B0 achieve lower but still competitive performance under CWT. MobileNet-V3 reaches 98.23% ± 0.47% accuracy and EfficientNet-B0 reaches 97.92% ± 0.43%, both exhibiting slightly larger standard deviations across folds compared to ResNet-18 and TinyViT-Hybrid, suggesting reduced stability. The confusion matrices for these two models show noticeably higher off-diagonal counts, with MobileNet-V3 producing mean false positive and false negative counts of 15.33 and 16.50 respectively, and EfficientNet-B0 producing 19.50 and 17.83 respectively, confirming their lower discriminative reliability relative to the residual-based models.

Comparing representations, CWT-based images yield the highest image-level accuracy across all four models for the SSRI dataset. VMD-based representations produce a modest but consistent reduction in performance, with ResNet-18 dropping to 98.84% ± 0.18% and TinyViT-Hybrid to 98.72% ± 0.26%. The confusion matrices for VMD confirm this trend: the ResNet-18 VMD matrix shows mean off-diagonal counts of 10.67 false positives and 10.17 false negatives, compared to only 4.50 and 5.67 for CWT. MobileNet-V3 and EfficientNet-B0 are more severely affected by the change in representation, falling to 97.19% and 96.75% accuracy respectively under VMD, with EfficientNet-B0 exhibiting the highest false positive count of 33.33 among all VMD configurations. These results suggest that, for the SSRI dataset, the multi-resolution continuous wavelet representation captures more discriminative time-frequency structure than the adaptive narrowband decomposition provided by VMD.

The fusion of CWT and VMD representations does not yield consistent improvements for SSRI therapy prediction. Under the fusion representation, ResNet-18 achieves 99.02% ± 0.24% accuracy, which is lower than the 99.43% obtained with CWT alone. A similar degradation is observed for all other models: MobileNet-V3 and EfficientNet-B0 achieve 97.21% and 97.08% respectively under fusion, compared to 98.23% and 97.92% with CWT. The confusion matrices for fusion-based models show higher false positive counts relative to CWT, particularly for MobileNet-V3 (28.33) and EfficientNet-B0 (34.50), indicating increased misclassification of non-R images as responders when the two representations are combined. This performance reduction suggests that the pixel-level averaging fusion introduces competing spectral information that partially obscures the discriminative patterns captured individually by CWT for SSRI therapy.

The learning curves in Figure 7, Figure 8 and Figure 9 provide further insight into the training dynamics of each model across all three representations. For CWT-based images, ResNet-18 and TinyViT-Hybrid exhibit the most stable convergence behaviour, with validation loss closely tracking training loss from epoch 1 and both quantities reaching near-zero values by epoch 8. The narrow standard deviation bands across folds confirm that these two models converge consistently regardless of the fold assignment. The validation accuracy for ResNet-18 and TinyViT-Hybrid under CWT starts at approximately 90% at epoch 1 and saturates near 99.5% by epoch 8, with very low inter-fold variance throughout training. MobileNet-V3 exhibits a noticeably wider standard deviation band in validation loss around epoch 5 under both CWT and fusion representations, indicating sensitivity to the particular fold composition. EfficientNet-B0 shows a persistent gap between training and validation loss across all three representations, with training loss remaining consistently higher than validation loss, suggesting that the model benefits from the generalisation properties of ImageNet pretraining but converges more slowly to the target distribution.

Under VMD-based images, all four models exhibit a steeper initial loss decline compared to CWT, with validation loss beginning at higher values in epoch 1 but dropping sharply by epoch 3. ResNet-18 and TinyViT-Hybrid again demonstrate the tightest inter-fold consistency, with standard deviation bands narrowing rapidly after epoch 2. MobileNet-V3 under VMD begins training with a notably high initial loss near 0.58 and wide confidence bands in early epochs, yet converges to a stable final accuracy of approximately 97%, consistent with its image-level result. For the fusion representation, convergence patterns closely resemble those of CWT for ResNet-18 and TinyViT-Hybrid, while MobileNet-V3 again displays elevated variance in validation loss around epoch 5, reflecting the broader instability of this architecture on the SSRI dataset. Across all representations, no model shows signs of overfitting within the 8-epoch training budget, as validation loss consistently decreases or stabilises without diverging from training loss.

The per-channel subject-level accuracy analysis presented in Figure 10 reveals clear spatial patterns in the discriminative information available for SSRI therapy prediction. Under CWT-based representations, the occipital channels O1 and O2 yield the highest subject-level accuracy, reaching 90% for both ResNet-18 and TinyViT-Hybrid, and 83.3% for EfficientNet-B0 and MobileNet-V3. This finding indicates that posterior cortical oscillatory activity, which is closely associated with alpha-band rhythms at rest, carries particularly informative pre-treatment signatures for identifying SSRI responders. The frontal channels F3, F4, F7, and Fp1 produce the lowest channel-level accuracy under CWT for most models, with ResNet-18 dropping to 61.1% at F3 and MobileNet-V3 to 60.8% at F3, reflecting the higher noise and artefact susceptibility of anterior electrodes in resting-state recordings. Under VMD, the channel accuracy profile shifts towards the temporal and central regions, with T3 and T4 yielding the highest accuracy for ResNet-18 (89.2%) and TinyViT-Hybrid (89.2%), while occipital channels remain informative but show less pronounced dominance compared to CWT. MobileNet-V3 achieves its peak channel accuracy of 85% at F4 under VMD, suggesting that the narrowband decomposition of VMD exposes different frequency-specific spatial patterns compared to the continuous multi-resolution representation of CWT. Under the fusion representation, the channel accuracy profiles become more uniform across the scalp, with most channels falling in the range of 70–85% and no single electrode showing a strong advantage, consistent with the slight overall performance degradation observed under fusion at the image level. These spatial differences between representations further support the observation that CWT and VMD encode complementary but non-redundant neural features, and that their combination through pixel-level averaging attenuates rather than reinforces the most discriminative spatial signals for SSRI response prediction.

At the subject level, Table 8 shows that the highest subject-level accuracy of 82.50% is achieved by multiple model–representation combinations, including CWT with ResNet-18, VMD with ResNet-18, VMD with TinyViT-Hybrid, Fusion with MobileNet-V3, and Fusion with EfficientNet-B0. However, all subject-level results are associated with relatively large standard deviations in the range of 11–17%, which reflects the limited number of test subjects per fold (five per fold: two responders and three non-responders) inherent to the subject-independent split of the 30-subject SSRI cohort. The wide 95% confidence intervals reported in Table 8 further underscore this variability. Notably, CWT with MobileNet-V3 yields the lowest subject-level accuracy of 71.67%, consistent with its lower image-level performance. Overall, the subject-independent results indicate that ResNet-18 and TinyViT-Hybrid generalise most reliably to unseen SSRI patients across all three time-frequency representations.

### 5.2. Performance of CAD System for rTMS Therapy

**Figure 11:**
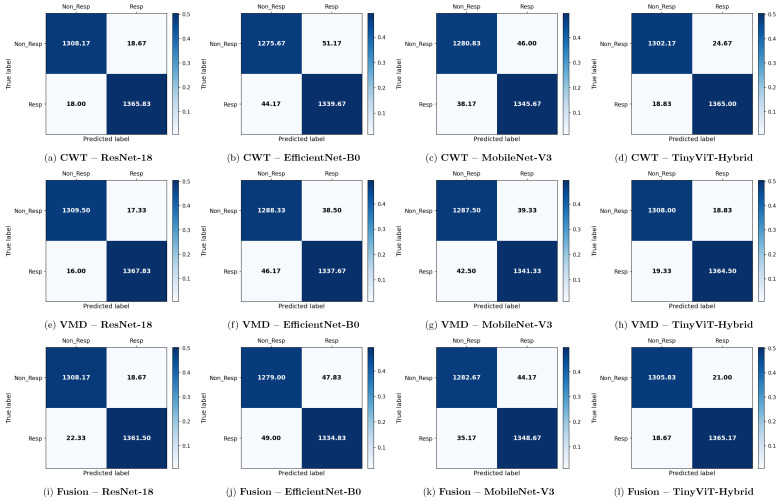
Average 6-fold confusion matrices for rTMS treatment response prediction under image-independent CV. Results are shown for three time-frequency representations—CWT (panels **a**–**d**), VMD (panels **e**–**h**), and Fusion (panels **i**–**l**)—and four CNN architectures (ResNet-18, EfficientNet-B0, MobileNet-V3, TinyViT-Hybrid). Each cell reports the mean count averaged across the six folds; non-integer values arise from this averaging. Rows indicate the true class label and columns the predicted label, where Resp = responder and Non_Resp = non-responder to rTMS therapy. The colour scale reflects the normalised proportion per cell. The approximate number of test images per fold is 2711 (∼1384R and ∼1327NR), reflecting the balanced class distribution of the rTMS dataset.

**Figure 12:**
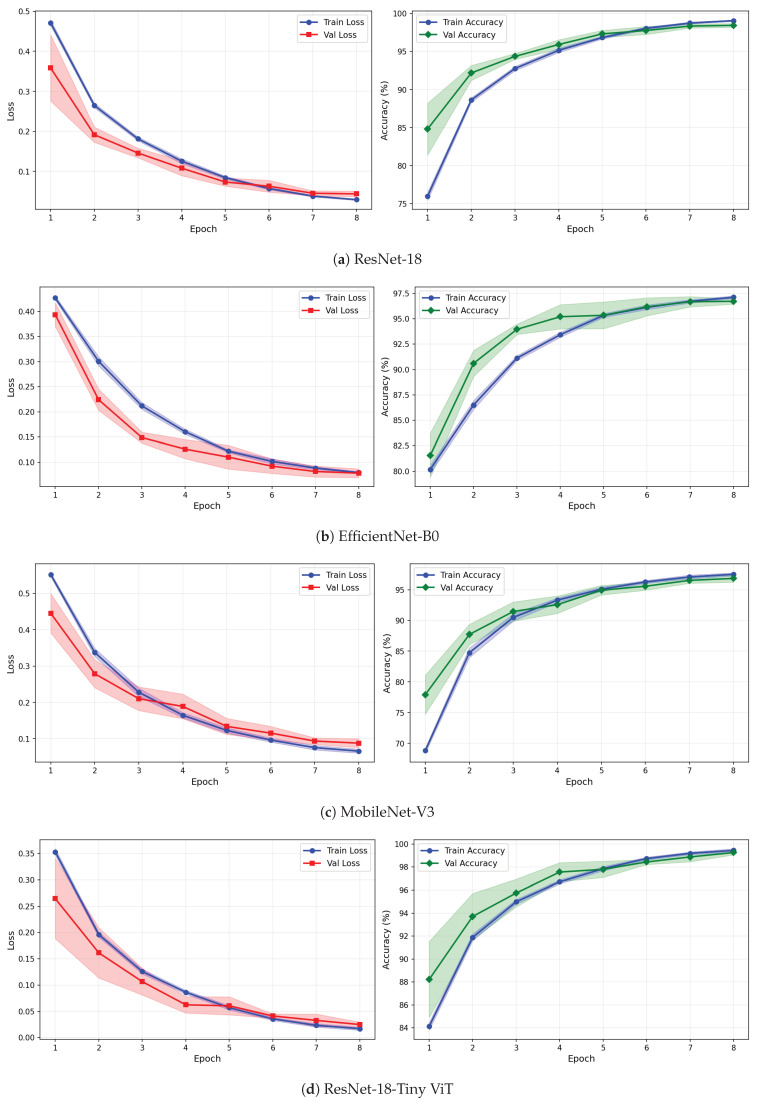
Training and validation loss and accuracy curves for CWT-based representations on the rTMS dataset under image-independent CV. Results are averaged across six folds with standard deviation shading.

**Figure 13:**
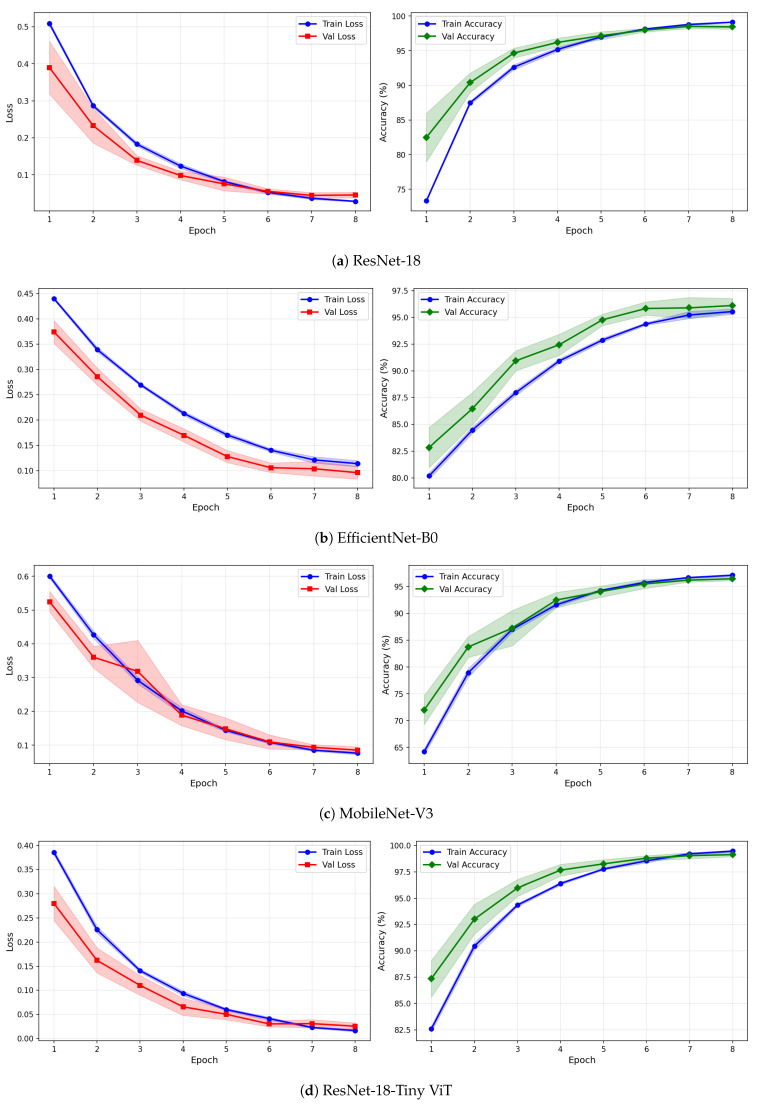
Training and validation loss and accuracy curves for VMD-based representations on the rTMS dataset under image-independent CV. Curves show mean performance across six folds with standard deviation shading.

**Figure 14:**
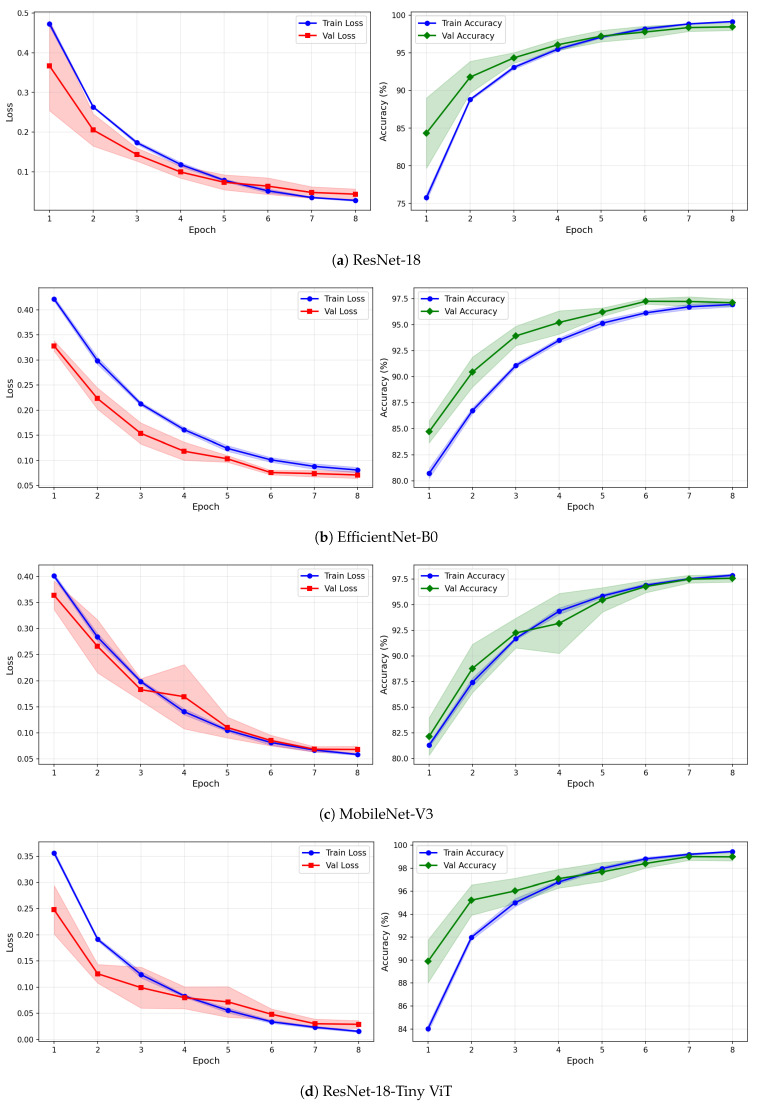
Training and validation loss and accuracy curves for the Fusion (CWT+VMD) representation on the rTMS dataset under image-independent CV. Shaded regions represent one standard deviation across six folds.

**Figure 15:**
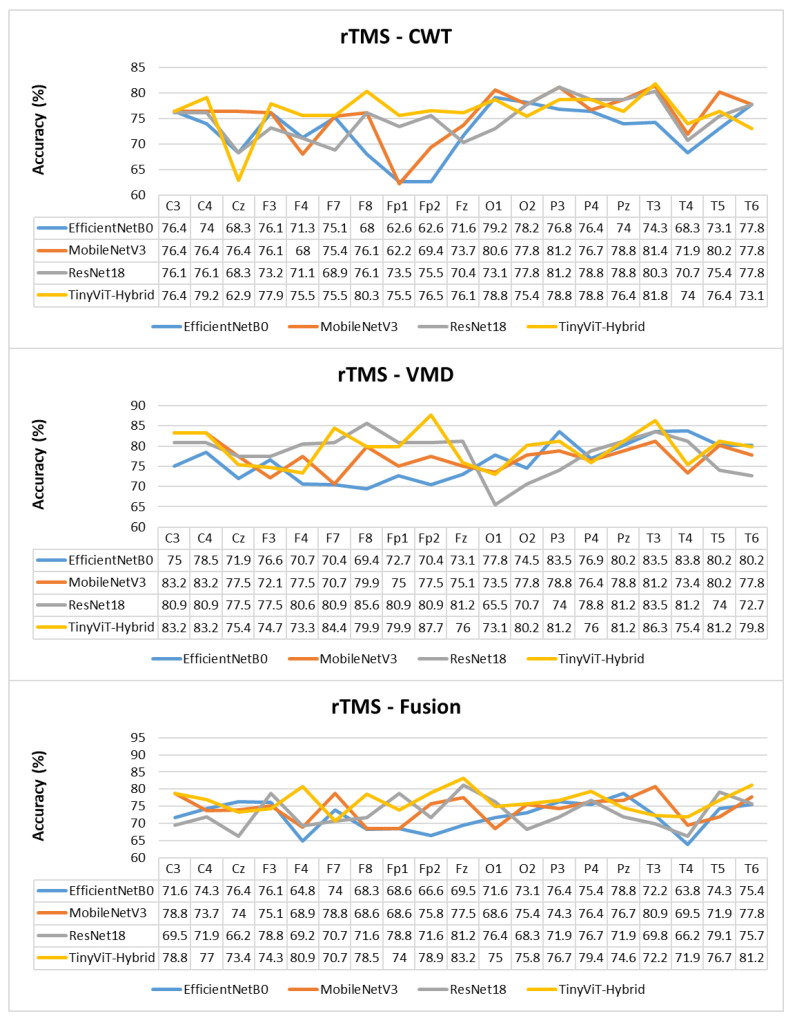
Per-channel subject-level accuracy (%) for the rTMS dataset under subject-independent CV across CWT, VMD, and Fusion representations and four CNN models.

Table 9 reports the image-level classification performance of the four pretrained CNN models under image-independent 6-fold CV for the rTMS dataset across all three time-frequency representations. Table 10 reports the corresponding subject-level accuracy under subject-independent CV. The average confusion matrices for all twelve model–representation combinations are visualised in Figure 11. The training and validation dynamics are presented in Figure 12, Figure 13 and Figure 14 for CWT, VMD, and Fusion representations respectively. The per-channel subject-level accuracy under subject-independent CV is shown in Figure 15.

The rTMS dataset presents a notably different pattern of results compared to SSRI therapy. Whereas CWT was the best-performing representation for SSRI, VMD-based images achieve the highest overall image-level accuracy for rTMS. ResNet-18 with VMD attains the best overall performance of 98.77% ± 0.14% accuracy, a precision of 98.75% ± 0.32%, a recall of 98.84% ± 0.30%, a specificity of 98.69% ± 0.34%, and an F1-score of 98.80% ± 0.13%. The corresponding confusion matrix confirms this, showing mean off-diagonal counts of only 18.67 false positives and 22.33 false negatives out of approximately 2710 test images per fold. TinyViT-Hybrid under VMD achieves closely comparable performance at 98.59% ± 0.28% accuracy with 21.00 false positives and 18.67 false negatives, demonstrating that the hybrid attention mechanism provides a modest complementary benefit over the plain residual architecture on the VMD representation for rTMS.

The superiority of VMD over CWT for rTMS therapy is consistent across all four models, though the margin is small for the stronger architectures. ResNet-18 improves from 98.65% under CWT to 98.77% under VMD, and TinyViT-Hybrid improves from 98.40% to 98.59%. For the lighter architectures the advantage of VMD is also evident: MobileNet-V3 improves from 96.90% to 96.98% and EfficientNet-B0 improves from 96.48% to 96.88%. The confusion matrices confirm this trend for EfficientNet-B0, where the VMD matrix shows 38.50 false positives and 46.17 false negatives compared to 51.17 and 44.17 respectively under CWT, indicating that VMD reduces non-R misclassification for this model in the rTMS setting. This result suggests that the adaptive narrowband decomposition of VMD captures spectral features of rTMS-relevant neural oscillations more effectively than the continuous multi-resolution representation of CWT, reflecting a therapy-specific difference in the discriminative information encoded in the EEG signal.

In contrast to the SSRI results, the fusion of CWT and VMD representations yields a more nuanced outcome for rTMS. ResNet-18 under fusion achieves 98.49% accuracy, which is slightly lower than the 98.77% obtained with VMD alone but comparable to the 98.65% obtained with CWT alone. TinyViT-Hybrid under fusion achieves 98.54% accuracy, marginally lower than its VMD counterpart. Importantly, MobileNet-V3 achieves its highest image-level accuracy of 97.07% ± 0.47% under the fusion representation, surpassing both its CWT (96.90%) and VMD (96.98%) results, which is the only instance across all experiments where fusion produces a clear improvement. EfficientNet-B0 under fusion achieves 96.43% accuracy, marginally lower than its VMD result, with 47.83 false positives and 49.00 false negatives, the highest false negative count among all rTMS configurations.

Across all representations and models, the rTMS results exhibit lower inter-fold standard deviations compared to SSRI, reflecting the larger size of the rTMS dataset and its balanced class distribution. The standard deviation of accuracy across the six folds is at most 0.47% for any model–representation combination in the rTMS dataset. ResNet-18 consistently achieves the smallest standard deviation across all three representations (0.34% for CWT, 0.14% for VMD, and 0.12% for fusion), confirming its superior stability relative to the other architectures for rTMS therapy prediction.

The learning curves in Figure 12, Figure 13 and Figure 14 reveal important differences in convergence behaviour between models and representations on the rTMS dataset. Under CWT, ResNet-18 and TinyViT-Hybrid demonstrate the most stable and rapid convergence, with validation loss closely tracking training loss from epoch 1, narrow standard deviation bands throughout training, and validation accuracy saturating near 98.5–99% by epoch 8. TinyViT-Hybrid exhibits a notably lower initial training loss of approximately 0.35 under CWT compared to ResNet-18 at 0.47, and its validation accuracy starts at 88% at epoch 1, the highest among all models under this representation, reflecting the benefit of its hybrid attention mechanism for capturing global spatial dependencies in CWT scalograms from the very first epoch. EfficientNet-B0 under CWT exhibits the largest persistent gap between training and validation loss across all eight epochs, with training loss remaining above validation loss throughout, and a wider standard deviation band in validation accuracy during epochs 2–5, indicating higher variability across folds. MobileNet-V3 under CWT shows broadly similar convergence to EfficientNet-B0 but with a somewhat tighter validation band after epoch 4.

Under VMD-based images, the convergence dynamics for ResNet-18 and TinyViT-Hybrid are qualitatively similar to CWT but with slightly higher initial loss values. ResNet-18 begins training at a loss of approximately 0.51 under VMD and descends smoothly to below 0.05 by epoch 8, with very narrow inter-fold standard deviation bands confirming consistent convergence. TinyViT-Hybrid under VMD shows the tightest validation loss band of any model–representation combination across the entire rTMS experiment, reaching near-zero validation loss by epoch 7 with negligible spread across folds, consistent with its strong and stable image-level accuracy. EfficientNet-B0 under VMD shows a persistent training-to-validation loss gap that remains visible at epoch 8, suggesting that this model continues to generalise better than it trains on VMD features, which is atypical and may reflect the mismatch between the VMD spectrogram’s spectral structure and the compound-scaling architecture of EfficientNet-B0. MobileNet-V3 under VMD exhibits a notably wide validation loss standard deviation band at epoch 4, caused by inter-fold variability in how quickly this model adapts to the VMD representation, though the band narrows substantially by epoch 6 and the model ultimately converges to a stable accuracy near 97%.

Under the fusion representation, the learning curve profiles of ResNet-18 and TinyViT-Hybrid remain stable and closely mimic their VMD counterparts, with validation accuracy reaching approximately 98.5% and 99% respectively by epoch 8. MobileNet-V3 under fusion displays a pronounced spike in the validation loss standard deviation band around epoch 5, a pattern also observed under CWT for the same model on the SSRI dataset, suggesting that MobileNet-V3 is systematically sensitive to fold composition when trained on mixed-domain representations. Despite this transient instability, the model recovers to a stable accuracy by epoch 7, consistent with its best image-level performance under fusion among the lighter architectures. EfficientNet-B0 under fusion shows the most gradual convergence of all rTMS configurations, with training accuracy still climbing noticeably between epochs 6 and 8 and the validation accuracy standard deviation band remaining wider than for ResNet-18 and TinyViT-Hybrid throughout training.

The per-channel subject-level accuracy analysis presented in Figure 15 reveals clear spatial and representation-dependent differences in the discriminative power of individual EEG channels for rTMS therapy prediction. Under VMD-based representations, the temporal and frontal regions yield the highest per-channel accuracy. ResNet-18 achieves its peak channel accuracy of 85.6% at F8 under VMD, and TinyViT-Hybrid reaches 87.7% at Fp2, also under VMD. MobileNet-V3 achieves peak channel accuracies of 83.2% at C3 and C4, and EfficientNet-B0 peaks at 83.5% at P3 and T3 under VMD. These results confirm that frontotemporal channels carry particularly strong discriminative information for rTMS response prediction, consistent with the known involvement of the prefrontal cortex and its connectivity with the default mode network in rTMS treatment effects. Under CWT-based representations, the channel accuracy profiles are more uniform and generally lower, with most models achieving peak accuracies between 78% and 82% at parietal and temporal channels including Pz, T3, and T5, and dropping noticeably at Fp1 and Fp2 for EfficientNet-B0 and MobileNet-V3, which reach as low as 62.6% and 62.2% respectively at those channels. This frontal weakness under CWT, also observed for SSRI but to a lesser extent, reflects the greater susceptibility of CWT scalograms to frontal artefact contamination in the resting-state EEG. Under the fusion representation, the channel accuracy profiles are intermediate between CWT and VMD for most channels, with TinyViT-Hybrid achieving its best fusion channel accuracy of 83.2% at Fz, and MobileNet-V3 peaking at 80.9% at T3. The overall flattening of the channel accuracy profile under fusion, combined with the reduction in peak accuracy relative to VMD, further supports the conclusion that pixel-level averaging of CWT and VMD representations partially attenuates the spatially localised discriminative features most relevant for rTMS outcome prediction.

At the subject level, Table 10 shows that the highest subject-level accuracy of 83.53% is jointly achieved by VMD with ResNet-18 and VMD with EfficientNet-B0, both with a standard deviation of 12.30% and a 95% CI of [72.81, 94.25]%. These results confirm that VMD-based representations are particularly effective for rTMS outcome prediction at the subject level, consistent with the image-level findings. CWT with MobileNet-V3 achieves the next highest subject-level accuracy of 81.15% ± 15.41%, while CWT with EfficientNet-B0 yields the lowest subject-level accuracy of 71.63% ± 21.63%, the widest CI among all rTMS configurations. Under the fusion representation, all four models achieve subject-level accuracy in the range of 71.63–76.69%, consistently lower than the best VMD results. Overall, the subject-independent results confirm that VMD with ResNet-18 provides the most reliable framework for predicting rTMS therapy outcomes in unseen patients.

Complete per-fold image-level classification results for all model–representation–dataset combinations (Tables S1–S24), reporting Accuracy, Precision, Recall, Specificity, and F1-Score across the six image-level CV folds together with the mean and standard deviation, enabling a transparent assessment of fold-to-fold variability and performance stability. Supplementary Materials S3: Per-fold training and validation loss and accuracy curves (Figures S5–S28) for all combinations of four models (ResNet-18, MobileNet-V3, EfficientNet-B0, TinyViT-Hybrid), three time-frequency representations (CWT, VMD, Fusion), and two datasets (SSRI, rTMS), each presented in a 2×3 grid layout for direct visual inspection of convergence behaviour and consistency across folds.

### 5.3. Comparative Performance and Architectural Analysis of the Evaluated Networks

To quantify whether the observed performance differences between architectures are statistically significant, pairwise Wilcoxon signed-rank tests were conducted on the per-fold accuracy scores across all model pairs, representations, and both therapy datasets. The resulting *p*-values are reported in Table 11, with a significance threshold of α=0.05. It should be noted that all statistical comparisons are based exclusively on image-level CV results, where the larger number of test samples per fold provides sufficient statistical power for pairwise testing. Subject-level results, which involve only a small number of test subjects per fold, are not included in this analysis as the limited sample size would preclude reliable non-parametric significance testing.

The statistical analysis reveals a clear and consistent two-tier architectural hierarchy across both datasets and all representations. ResNet-18 and TinyViT-Hybrid form a statistically indistinguishable pair in every experimental condition, with all pairwise *p*-values exceeding 0.08 for SSRI and 0.22 for rTMS. This confirms that the addition of the Transformer encoder to the ResNet-18 backbone does not yield a statistically meaningful accuracy gain over the plain residual architecture within the training budget used in this study.

Both residual-family models are significantly superior to MobileNet-V3 and EfficientNet-B0 across nearly all conditions, with *p*-values consistently below 0.001 for both datasets. The performance gap between the two tiers becomes most pronounced under the fusion representation, where the lighter architectures appear less capable of simultaneously integrating the mixed-domain features arising from the pixel-level combination of CWT and VMD images. The comparison between EfficientNet-B0 and MobileNet-V3 is inconsistent across conditions, reaching significance only under CWT for SSRI (p=0.0263) and under fusion for rTMS (p=0.049), while remaining non-significant in all other cases. This indicates that these two architectures occupy a statistically equivalent performance tier, and the choice between them should be guided by deployment constraints such as model size and inference latency rather than classification accuracy.

### 5.4. Influence of Time-Frequency Image Type on Classification Efficacy

To assess whether the choice of time-frequency representation significantly affects classification performance, pairwise Wilcoxon signed-rank tests were conducted on the per-fold image-level accuracy scores across the three representations for each therapy dataset. All accuracy scores were pooled across the four CNN architectures within each therapy to maximise statistical power. The resulting *p*-values are summarised in Table 12, with a significance threshold of α=0.05. As with the architectural comparisons reported in Section 5.3, all statistical tests are based exclusively on image-level CV results, as the limited number of test subjects per fold in the subject-independent setting does not provide sufficient statistical power for reliable non-parametric significance testing.

The results reveal a clear therapy-dependent pattern in how much the choice of representation influences classification performance. For the SSRI dataset, CWT is significantly superior to both VMD and fusion, with *p*-values below 0.0001 for both comparisons, confirming that the multi-resolution continuous wavelet representation captures substantially more discriminative information for SSRI therapy prediction than either the adaptive narrowband VMD decomposition or their pixel-level combination. The comparison between VMD and fusion for SSRI does not reach significance (p=0.0826), indicating that fusing CWT and VMD through pixel-level averaging does not produce a representation that is statistically distinguishable from VMD alone in this therapy context. Taken together, these findings confirm that CWT is the representation of choice for SSRI therapy, and that fusion provides no statistically meaningful benefit over the individual representations.

For the rTMS dataset, the representation differences are smaller in magnitude and the statistical picture is more nuanced. CWT and VMD differ significantly (p=0.045), with VMD yielding higher accuracy as reported in Section 5.2, confirming that the adaptive decomposition of VMD is statistically superior to CWT for rTMS outcome prediction. In contrast, CWT and fusion are not significantly different (p=0.732), and VMD versus fusion only approaches significance (p=0.057), suggesting that for rTMS the three representations produce broadly comparable performance at the image level even though VMD holds a marginal numerical advantage. These results indicate that the rTMS classification task is less sensitive to the choice of time-frequency representation than SSRI, likely reflecting the more balanced class distribution and larger dataset size of the rTMS cohort, which allows all three representations to extract sufficient discriminative information. Overall, the statistical analysis of representation effects reinforces the conclusion that the optimal time-frequency image type is therapy-specific: CWT is preferred for SSRI and VMD is preferred for rTMS, while fusion does not consistently improve upon the best single-representation result in either therapy context.

### 5.5. Comparison with State-of-the-Art Studies

Table 13 summarises the most relevant prior studies on EEG-based prediction of depression therapy outcomes alongside the best image-level results achieved by the proposed framework. Direct numerical comparison across studies should be interpreted with caution, as differences in dataset size, CV strategy, feature extraction pipeline, and class balance can substantially influence reported accuracy figures.

Early studies on EEG-based SSRI response prediction relied on hand-crafted spectral and connectivity features combined with shallow classifiers. Mumtaz et al. [14] combined wavelet transform, STFT, and empirical mode decomposition features with logistic regression under 10-fold CV, achieving 91.6% accuracy on the SSRI dataset. Khodayari et al. [15] extracted power spectral density, coherence, and multifractal analysis features and reported 87.4% accuracy with an 80/20 train-test split. While these approaches established the feasibility of EEG-based SSRI outcome prediction, their dependence on manually engineered features limits their capacity to capture the complex nonlinear and non-stationary dynamics inherent in pre-treatment EEG recordings.

More recent deep learning approaches have substantially improved performance by learning representations directly from structured EEG inputs. Shahabi et al. [5] applied an ensemble transfer learning framework to CWT images for SSRI prediction, achieving 96.55% accuracy under 5-fold CV. A subsequent study by the same group [21] incorporated a recurrent attention mechanism applied to raw EEG signals and reported 98.84% accuracy, which represents the strongest prior result on the SSRI dataset. For rTMS therapy, Shahabi et al. [7] combined CWT images with a CNN-LSTM-Attention architecture and achieved 97.1% accuracy, while a bidirectional LSTM ensemble model applied to raw EEG signals reached 98.51% [24].

The proposed framework achieves 99.43% image-level accuracy for SSRI using CWT images with ResNet-18, surpassing all prior SSRI results including the recurrent attention model of [21]. For rTMS therapy, the proposed VMD-based ResNet-18 framework attains 98.77% accuracy, exceeding the CWT-CNN-LSTM-Attention result of [7] and closely matching the bidirectional LSTM ensemble of [24]. Notably, these competitive results are achieved without any recurrent or attention layers in the classification network, relying instead on the inductive bias of pretrained residual convolutional features applied to carefully constructed time-frequency images. This demonstrates that the choice of time-frequency representation is at least as important as architectural complexity for EEG-based therapy prediction, and that simple fine-tuning of a pretrained ResNet-18 on well-designed spectral images can match or outperform substantially more complex sequence modelling architectures. Furthermore, unlike most prior studies that evaluate a single therapy modality, the proposed framework provides a unified comparative analysis across both SSRI and rTMS therapies, revealing the therapy-specific nature of the optimal time-frequency representation, a finding that has direct implications for the design of future EEG-based depression therapy prediction systems.

The proposed CAD system offers several notable advantages over existing approaches for EEG-based depression therapy outcome prediction.

- Therapy-specific representation analysis: The framework systematically compares three time-frequency representations across two therapy datasets, revealing that the optimal representation is therapy-specific: CWT for SSRI and VMD for rTMS. This finding has direct implications for the design of future EEG-based treatment selection systems.
- No recurrent or attention layers required: State-of-the-art image-level accuracy is achieved by simply fine-tuning a pretrained ResNet-18 on well-constructed time-frequency images, without any recurrent or attention mechanisms, reducing training complexity and computational cost relative to LSTM- or Transformer-based classifiers.
- Computationally efficient: All four architectures are lightweight pretrained CNNs that converge within 8 epochs on a standard GPU, making the framework practical for clinical research settings where computational resources and labelled data are limited.
- Rigorous dual-strategy evaluation: The system is assessed under both image-independent and subject-independent 6-fold CV, providing both an upper-bound discrimination estimate and a clinically realistic generalisation estimate, complemented by per-channel accuracy analysis across all 19 EEG electrodes.
- Adaptive decomposition via VMD: VMD adaptively decomposes EEG into narrowband intrinsic mode functions with fine frequency localisation, capturing oscillatory components inaccessible to fixed-scale wavelet analysis. Its statistically confirmed superiority for rTMS demonstrates the value of adaptive decomposition in EEG-based deep learning pipelines.
- Statistically validated conclusions: All findings on architectural hierarchy and representation efficacy are supported by pairwise Wilcoxon signed-rank tests, confirming that observed differences are genuine and reproducible rather than artefacts of fold assignment.
- Spatial interpretability: Per-channel accuracy analysis identifies occipital dominance for SSRI under CWT and frontotemporal dominance for rTMS under VMD, consistent with known neurophysiological mechanisms and offering guidance for electrode selection in resource-constrained deployments.

Despite the promising results, several limitations of the present study should be acknowledged. First, the patient groups are small (30 SSRI and 46 rTMS patients), and although the subject-independent CV provides a realistic estimate of generalisation, the subject-level accuracy values are associated with wide CIs that reflect this limited sample size. Replication on larger, multi-centre datasets is therefore necessary before any clinical conclusions can be drawn. Second, the absence of an external independent validation cohort is a key limitation. The subject-independent CV results provide a realistic estimate of generalisation to unseen patients within each cohort, but multi-centre external validation is necessary before clinical deployment. Third, the fusion strategy evaluated here-pixel-wise arithmetic averaging of per-image normalised representations-represents one of many possible fusion paradigms. Feature-level, channel-wise, or attention-guided late-fusion strategies may yield different results and are identified as a direction for future work. Fourth, the deep learning models employed in this study offer limited interpretability. Future work should integrate explainable AI techniques such as gradient-weighted class activation mapping (Grad-CAM) to identify the EEG frequency bands, temporal segments, and scalp regions that contribute most to treatment-response predictions, thereby supporting transparent and clinically interpretable decision-making by psychiatrists. Finally, no data augmentation was applied in this study; exploring augmentation strategies tailored to EEG time-frequency images may further improve generalisation in low-sample settings.

## 6. Conclusions

This study presented a comprehensive deep learning framework for predicting depression therapy outcomes from pre-treatment EEG signals using three time-frequency representations and four pretrained CNN architectures evaluated on two independent therapy datasets. The key findings can be summarised as follows.

The choice of time-frequency representation is therapy-specific and has a statistically significant impact on classification performance. CWT-based scalograms yield superior discrimination for SSRI therapy prediction, with ResNet-18 achieving 99.43% image-level accuracy, while VMD-based spectrograms are statistically superior for rTMS outcome prediction, with ResNet-18 reaching 98.77%. Pixel-level fusion of CWT and VMD does not consistently improve performance over the best single representation in either therapy context. These findings demonstrate that the neurophysiological signatures predictive of SSRI and rTMS response are encoded in different spectral-temporal structures, and that selecting the representation to match the therapy is a critical design decision for EEG-based CAD systems.

A clear two-tier architectural hierarchy was confirmed by pairwise Wilcoxon signed-rank tests. ResNet-18 and TinyViT-Hybrid are statistically indistinguishable and both significantly outperform MobileNet-V3 and EfficientNet-B0 across all conditions and both datasets. Notably, competitive results are achieved without any recurrent or attention mechanisms, demonstrating that careful signal representation combined with a simple pretrained residual network can match or surpass substantially more complex architectures reported in prior work.

Per-channel analysis revealed that occipital channels carry the most discriminative information for SSRI response under CWT, while frontotemporal channels are most predictive for rTMS response under VMD, findings that are consistent with known neurophysiological mechanisms and offer practical guidance for electrode selection in resource-constrained clinical settings. At the subject level, the framework achieves 82.50% and 83.53% accuracy for SSRIs and rTMS respectively under strict subject-independent CV, highlighting the remaining challenge of generalisation to unseen patients in small clinical cohorts.

Future work should focus on validating the proposed framework on larger, multi-centre datasets to improve subject-level generalisation, and on integrating explainable AI methods to identify the EEG biomarkers and frequency bands that are most informative for each therapy, thereby supporting transparent and clinically interpretable treatment selection. 

## Figures and Tables

**Table 1:** A summary of previous works on predicting outcomes of depression therapy. All reported accuracy values correspond to image-level classification results.

Ref, Year	Methods	Therapy	No. of Patients	Accuracy
[14]	Wavelet transform + STFT + EMD; ROC analysis; logistic regression	SSRI	16 R vs. 18 NR	91.60%
[15]	Nonlinear EEG features; Fisher discriminant ratio; multifractal analysis classifier	SSRI	11 R vs. 11 NR	87.40%
[16]	EEG biomarkers + clinical variables; Student’s *t*-test; SVM	SSRI	155 R vs. 67 NR	82.40%
[17]	Demographic + EEG + source-localised features; PCA; random forest	SSRI	27 R vs. 24 NR	88.00%
[18]	Absolute/relative EEG band power + beta-to-alpha ratio; gradient boosted decision tree	SSRI	528 patients	C-index = 0.963
[19]	EEG signal signatures; sequential least-squares regression	SSRI	309 patients	RMSE = 5.68
[5]	CWT scalogram images; majority voting ensemble of five pretrained TL-CNN models	SSRI	12 R vs. 18 NR	96.55%
[20]	Low-resolution brain tomography; functional connectivity + coherence; ROC	SSRI	12 R vs. 18 NR	–
[21]	Cascaded pretrained TL pipeline; biologically inspired LSTM; raw EEG	SSRI	12 R vs. 18 NR	98.84%
[22]	Inter-channel brain rhythm connectivity; four sequential TL models; LSTM	SSRI	12 R vs. 18 NR	98.33%
[23]	EEG channel connectivity images; voting-based TL ensemble + LSTM	rTMS	23 R vs. 23 NR	99.32%
[7]	CWT scalogram images; fine-tuned TL backbone; biologically inspired LSTM	rTMS	23 R vs. 23 NR	97.10%
[24]	Pretrained TL ensemble; biologically inspired LSTM; raw EEG sequences	rTMS	23 R vs. 23 NR	98.51%
[25]	EEG inter-channel connectivity; pretrained TL ensemble	rTMS	34 patients	92.28%
Proposed	CWT/VMD/Fusion images; ResNet-18, MobileNet-V3, EfficientNet-B0, TinyViT-Hybrid	SSRI/rTMS	30/46 patients	99.43% / 98.77%

**Table 2:** Number of images per subject for SSRI and rTMS datasets.

SSRI Therapy				rTMS Therapy			
R		NR		R		NR	
Subject	Images	Subject	Images	Subject	Images	Subject	Images
1	380	1	361	1	380	1	380
2	361	2	361	2	361	2	342
3	209	3	361	3	323	3	361
4	380	4	361	4	361	4	266
5	380	5	361	5	342	5	285
6	361	6	361	6	190	6	399
7	361	7	380	7	760	7	361
8	380	8	380	8	304	8	456
9	361	9	361	9	380	9	323
10	361	10	361	10	380	10	380
11	361	11	380	11	361	11	361
12	361	12	380	12	380	12	247
–	–	13	361	13	361	13	361
–	–	14	304	14	285	14	399
–	–	15	380	15	380	15	304
–	–	16	361	16	342	16	399
–	–	17	361	17	342	17	342
–	–	18	361	18	304	18	399
–	–	–	–	19	361	19	190
–	–	–	–	20	323	20	342
–	–	–	–	21	342	21	342
–	–	–	–	22	361	22	342
–	–	–	–	23	380	23	380
Total	4256	Total	6536	Total	8303	Total	7961

**Table 3:** Summary of image generation parameters for CWT, VMD, and Fusion representations.

Parameter	CWT	VMD	Fusion
Method	Continuous Wavelet Transform	Variational Mode Decomposition + STFT	Pixel-wise average of CWT and VMD
Mother wavelet/window	Analytic Morlet (amor)	Hamming window (256 samples)	–
Frequency resolution	12 voices per octave	7680 FFT points	Inherited from CWT and VMD
Frequency range	2–60 Hz	0–60 Hz	0–60 Hz
Number of modes/overlap	–	K=20 IMFs; overlap = 250 samples (step = 6)	–
Penalty parameter α	–	2000 (MATLAB default)	–
Convergence tolerance ε	–	$10^{-7}$	–
Normalisation	Magnitude |W(a,b)| mapped to pixel intensity	Magnitude $|S_{\mathrm{VMD}}\left(t,f\right)|$ mapped to pixel intensity	Per-image min-max to [0,1] via mat2gray; pixel-wise average
Colourmap	Jet	Jet	Jet
Output format	PNG, borderless	PNG, borderless	PNG, borderless
Output size (CNN input)	224×224 pixels for all models		

**Table 4:** Architecture configurations and shared training hyperparameters for the four pretrained CNN models.

Parameter	ResNet-18	MobileNet-V3	EfficientNet-B0	TinyViT-Hybrid
Pretrained weights	ImageNet	ImageNet	ImageNet	ImageNet (backbone)
Backbone output dim	512	960	1280	512
Classifier head	Linear	3-layer MLP	2-layer MLP	Transformer + Linear
Transformer layers	–	–	–	2
Attention heads	–	–	–	8
Token dimension	–	–	–	512
Number of tokens	–	–	–	49 (7 × 7)
MLP ratio	–	–	–	4.0
Dropout	–	–	–	0.1
Output classes	2	2	2	2
**Shared Training Hyperparameters**				
Input resolution	224×224			
Batch size	32			
Optimiser	AdamW			
Learning rate	$1\times 10^{-4}$			
Weight decay	$1\times 10^{-4}$			
LR scheduler	Cosine annealing ($T_{\max}=8$, $\eta_{\min}=1\times 10^{-6}$)			
Loss function	Cross-entropy			
Max epochs	8			
Early stopping patience	3 (monitor: validation loss)			
Normalisation (mean)	[0.485,0.456,0.406]			
Normalisation (std)	[0.229,0.224,0.225]			
Random seed	42 (incremented by 100×foldindex per fold)			
Data augmentation	Not used			

**Table 5:** Number of R and NR EEG images in the SSRI and rTMS datasets.

Dataset	R Images	NR Images
SSRI	4256	6536
rTMS	8303	7961

**Table 6:** Summary of the two CV strategies employed in this study.

Property	Image-Independent CV	Subject-Independent CV
**Purpose**	Assess upper-bound image-level discrimination between R and non-R classes	Assess generalisation to completely unseen patients, simulating real clinical deployment
**Partitioning unit**	Individual images	Subjects (patients)
**Number of folds**	6	6
**SSRI test fold**	Stratified 1/6 of all images	responders + 3 non-responders
		(5 subjects per fold, 30 total)
**rTMS test fold**	Stratified 1/6 of all images	Folds 1–5: 4R + 4NR;
		Fold 6: 3R + 3NR
		(46 subjects total)
**Training (%) / validation split (%)**	90/10	90/10
	(stratified, from non-test images)	(stratified by subject)
**Can train and test images share**	Yes, images from the same subject may	No, all images of a given subject are in
**the same subject?**	appear in both sets	one set only
**Prediction level**	Each image is classified independently	Images of each test subject are aggregated by a majority-vote
**Reported metrics**	Accuracy, Precision, Recall, Specificity, F1-Score	Subject-level Accuracy, (95% CI)
	(mean ± std over 6 folds)	
**Interpretation**	Upper-bound estimate of image-level class separability	Clinically realistic estimate; reflects true patient-level generalisation performance

**Table 7:** Image-level classification performance (%) for the SSRI dataset using three time–frequency representations (CWT, VMD, and Fusion) and four deep learning models under image-level CV. Values are reported as mean ± standard deviation across 6 folds.

Img Type	Model	Acc	Std	Prec	Std	Rec	Std	Spec	Std	F1	Std
CWT	EfficientNet-B0	97.92	0.43	97.27	1.01	97.49	0.55	98.21	0.68	97.37	0.53
CWT	MobileNet-V3	98.23	0.47	97.85	1.04	97.67	0.72	98.59	0.69	97.76	0.59
CWT	ResNet-18	99.43	0.14	99.37	0.43	99.20	0.37	99.59	0.28	99.28	0.18
CWT	TinyViT-Hybrid	99.28	0.16	99.16	0.49	99.01	0.26	99.45	0.33	99.08	0.21
VMD	EfficientNet-B0	96.75	0.55	95.39	1.61	96.45	0.57	96.94	1.13	95.91	0.66
VMD	MobileNet-V3	97.19	0.41	96.60	1.17	96.29	0.74	97.78	0.79	96.44	0.50
VMD	ResNet-18	98.84	0.18	98.50	0.54	98.57	0.47	99.02	0.36	98.53	0.23
VMD	TinyViT-Hybrid	98.72	0.26	98.68	0.37	98.07	0.43	99.14	0.24	98.37	0.33
Fusion	EfficientNet-B0	97.08	0.42	95.26	1.01	97.46	0.56	96.83	0.71	96.34	0.51
Fusion	MobileNet-V3	97.21	0.34	96.06	1.01	96.92	0.36	97.40	0.71	96.48	0.41
Fusion	ResNet-18	99.02	0.24	98.72	0.69	98.80	0.36	99.16	0.46	98.76	0.30
Fusion	TinyViT-Hybrid	98.82	0.35	98.50	0.75	98.52	0.40	99.02	0.49	98.51	0.44

**Table 8:** Subject-level classification accuracy (%) for the SSRI dataset under subject-independent CV.

Img Type	Model	Acc	Std	CI Low	CI High
CWT	EfficientNet-B0	79.17	16.44	64.64	93.70
CWT	MobileNet-V3	71.67	14.34	55.55	87.79
CWT	ResNet-18	82.50	14.07	68.90	96.10
CWT	TinyViT-Hybrid	78.33	11.79	63.59	93.07
VMD	EfficientNet-B0	76.94	12.56	61.87	92.01
VMD	MobileNet-V3	76.94	12.56	61.87	92.01
VMD	ResNet-18	82.50	14.07	68.90	96.10
VMD	TinyViT-Hybrid	82.50	14.07	68.90	96.10
Fusion	EfficientNet-B0	82.50	14.07	68.90	96.10
Fusion	MobileNet-V3	82.50	14.07	68.90	96.10
Fusion	ResNet-18	78.33	11.79	63.59	93.07
Fusion	TinyViT-Hybrid	78.33	11.79	63.59	93.07

Note: Subject-level predictions are obtained by majority voting over all images belonging to each subject. Because the number of subjects per fold is small (five subjects per fold in a 6-fold scheme over 30 subjects), the discrete nature of majority-vote outcomes means that only a limited set of accuracy values is achievable per fold. Consequently, identical mean accuracy and CI values across different model–representation configurations are an inherent artefact of this granularity and do not indicate a reporting error.

**Table 9:** Image-level classification performance (%) for the rTMS dataset using three time–frequency representations (CWT, VMD, and Fusion) and four deep learning models under image-level CV. Values are reported as mean ± standard deviation across six folds.

Img Type	Model	Acc	Std	Prec	Std	Rec	Std	Spec	Std	F1	Std
CWT	EfficientNet-B0	96.48	0.43	96.33	0.76	96.81	0.72	96.14	0.85	96.56	0.42
CWT	MobileNet-V3	96.90	0.22	96.70	0.58	97.24	0.45	96.53	0.64	96.97	0.21
CWT	ResNet-18	98.65	0.34	98.66	0.63	98.70	0.32	98.59	0.67	98.68	0.33
CWT	TinyViT-Hybrid	98.40	0.19	98.23	0.28	98.64	0.49	98.14	0.30	98.43	0.19
VMD	EfficientNet-B0	96.88	0.32	97.20	0.32	96.66	0.51	97.10	0.35	96.93	0.32
VMD	MobileNet-V3	96.98	0.35	97.15	0.42	96.93	0.41	97.04	0.45	97.04	0.34
VMD	ResNet-18	98.77	0.14	98.75	0.32	98.84	0.30	98.69	0.34	98.80	0.13
VMD	TinyViT-Hybrid	98.59	0.28	98.64	0.57	98.60	0.36	98.58	0.60	98.62	0.27
Fusion	EfficientNet-B0	96.43	0.34	96.54	0.53	96.46	0.59	96.39	0.58	96.50	0.34
Fusion	MobileNet-V3	97.07	0.47	96.83	0.48	97.46	0.72	96.67	0.52	97.14	0.46
Fusion	ResNet-18	98.49	0.12	98.65	0.39	98.39	0.50	98.59	0.42	98.52	0.12
Fusion	TinyViT-Hybrid	98.54	0.28	98.49	0.56	98.65	0.26	98.42	0.59	98.57	0.27

**Table 10:** Subject-level classification accuracy (%) for the rTMS dataset under subject-independent CV.

Img Type	Model	Acc	Std	CI Low	CI High
CWT	EfficientNet-B0	71.63	21.63	58.60	84.66
CWT	MobileNet-V3	81.15	15.41	69.85	92.45
CWT	ResNet-18	78.77	13.26	66.95	90.59
CWT	TinyViT-Hybrid	76.39	17.53	64.12	88.66
VMD	EfficientNet-B0	83.53	12.30	72.81	94.25
VMD	MobileNet-V3	77.82	5.58	65.81	89.83
VMD	ResNet-18	83.53	12.30	72.81	94.25
VMD	TinyViT-Hybrid	81.15	10.06	69.85	92.45
Fusion	EfficientNet-B0	76.39	21.06	64.12	88.66
Fusion	MobileNet-V3	76.39	21.06	64.12	88.66
Fusion	ResNet-18	71.63	21.63	58.60	84.66
Fusion	TinyViT-Hybrid	76.69	14.61	64.47	88.91

Note: Subject-level predictions are obtained by majority voting over all images belonging to each subject. Because the number of subjects per fold is small (approximately 7–8 subjects per fold in a 6-fold scheme over 46 subjects), the discrete nature of majority-vote outcomes means that only a limited set of accuracy values is achievable per fold. Consequently, identical mean accuracy and CI values across different model–representation configurations are an inherent artefact of this granularity and do not indicate a reporting error.

**Table 11:** Pairwise statistical comparison (Wilcoxon signed-rank test, *p*-values) of per-fold image-level accuracy between the four CNN architectures across three time-frequency representations for the SSRI and rTMS datasets.

Dataset	Model Pair	CWT	VMD	Fusion
SSRI	EfficientNet-B0 vs. MobileNet-V3	0.0263	0.303	0.427
	EfficientNet-B0 vs. ResNet-18	0.0003	0.0003	0.0001
	EfficientNet-B0 vs. TinyViT-Hybrid	0.0002	0.0004	<0.0001
	MobileNet-V3 vs. ResNet-18	0.0009	0.0001	0.0001
	MobileNet-V3 vs. TinyViT-Hybrid	0.001	0.0009	<0.0001
	ResNet-18 vs. TinyViT-Hybrid	0.084	0.384	0.236
rTMS	EfficientNet-B0 vs. MobileNet-V3	0.0759	0.678	0.049
	EfficientNet-B0 vs. ResNet-18	<0.0001	<0.0001	<0.0001
	EfficientNet-B0 vs. TinyViT-Hybrid	0.0001	<0.0001	<0.0001
	MobileNet-V3 vs. ResNet-18	<0.0001	0.0001	0.0015
	MobileNet-V3 vs. TinyViT-Hybrid	0.0001	0.0003	0.0005
	ResNet-18 vs. TinyViT-Hybrid	0.224	0.257	0.666

**Table 12:** Pairwise statistical comparison (Wilcoxon signed-rank test, *p*-values) of per-fold image-level accuracy between the three time-frequency representations for the SSRI and rTMS datasets.

Representation Pair	SSRI	rTMS
CWT vs. VMD	<0.0001	0.045
CWT vs. Fusion	<0.0001	0.732
VMD vs. Fusion	0.0826	0.057

**Table 13:** Comparison with state-of-the-art studies on EEG-based prediction of depression therapy outcome on image level.

Study	Therapy	Model	CV	Accuracy (%)
[14]	SSRI	WT + STFT + EMD + LR	10-fold	91.6
[15]	SSRI	PSD + Coherence + MFA	80/20	87.4
[5]	SSRI	CWT + Ensemble TL	5-fold	96.55
[21]	SSRI	Raw EEG + TL-LSTM-Att	5-fold	98.84
[7]	rTMS	CWT + CNN-LSTM-Attention	5-fold	97.1
[24]	rTMS	Raw EEG + TL-BLSTM Ensemble	5-fold	98.51
Proposed (SSRI)	SSRI	CWT + ResNet-18	6-fold	99.43
Proposed (rTMS)	rTMS	VMD + ResNet-18	6-fold	98.77

## Data Availability

The SSRI dataset used in this study are publicly available on Figshare at https://figshare.com/articles/dataset/EEG_Data_New/4244171 (accessed on 1 January 2026). The rTMS dataset was collected at Atieh Hospital, Tehran, Iran, under the ethical approval of Shahid Beheshti University of Medical Sciences, and is available from the corresponding author upon reasonable request.

## Supplementary Materials

The following supporting information can be downloaded at: https://www.mdpi.com/article/10.3390/brainsci16030301/s1, Supplementary Materials S1: Detailed architectural diagrams of the four pretrained convolutional neural network models evaluated in this study, namely ResNet-18 (Figure S1), MobileNet-V3 (Figure S2), EfficientNet-B0 (Figure S3), and TinyViT-Hybrid (Figure S4), accompanied by descriptive text explaining the layer structure, spatial resolution evolution, and task-specific modifications applied to each model (Sections S1–S4). Supplementary Materials S2: Complete per-fold image-level classification results for all model–representation–dataset combinations (Tables S1–S24), reporting Accuracy, Precision, Recall, Specificity, and F1-Score across the six image-level CV folds together with the mean and standard deviation, enabling a transparent assessment of fold-to-fold variability and performance stability. Supplementary Materials S3: Per-fold training and validation loss and accuracy curves (Figures S5–S28) for all combinations of four models (ResNet-18, MobileNet-V3, EfficientNet-B0, TinyViT-Hybrid), three time-frequency representations (CWT, VMD, Fusion), and two datasets (SSRI, rTMS), each presented in a 2×3 grid layout for direct visual inspection of convergence behaviour and consistency across folds.

## Abbreviations

ACC	Accuracy
AdamW	Adaptive Moment Estimation with Weight Decay
ASR	Artifact Subspace Reconstruction
BDI	Beck Depression Inventory
CAD	Computer-Aided Decision
CAR	Common Average Reference
CI	Confidence Interval
CNN	Convolutional Neural Network
CWT	Continuous Wavelet Transform
EEG	Electroencephalography
EMD	Empirical Mode Decomposition
F1	F1-score
FFT	Fast Fourier Transform
FN	False Negative
FP	False Positive
GPU	Graphics Processing Unit
Grad-CAM	Gradient-weighted Class Activation Mapping
ICA	Independent Component Analysis
IMF	Intrinsic Mode Function
LR	Logistic Regression
LSTM	Long Short-Term Memory
MFA	Multifractal Analysis
MLP	Multi-Layer Perceptron
MSPCA	Multiscale Principal Component Analysis
NR	Non-Responder
PCA	Principal Component Analysis
PNG	Portable Network Graphics
PRE	Precision
PSD	Power Spectral Density
R	Responder
REC	Recall
ROC	Receiver Operating Characteristic
rTMS	Repetitive Transcranial Magnetic Stimulation
SPE	Specificity
SSRI	Selective Serotonin Reuptake Inhibitor
STD	Standard Deviation
STFT	Short-Time Fourier Transform
SVM	Support Vector Machine
TL	Transfer Learning
TN	True Negative
TP	True Positive
VMD	Variational Mode Decomposition
ViT	Vision Transformer
