# Supplementary material for: Towards the Development of a Deep Learning Framework Using Adaptive and Non-Adaptive Time-Frequency Features for EEG-Based Depression Therapy Prediction
Source: Brain Sci. 2026 Mar 9;16(3):301. doi: 10.3390/brainsci16030301 (PMC13025224; doi:10.3390/brainsci16030301)
Supplement: Supplementary file 1 [file brainsci-16-00301-s001.zip › brainsci-4091926-supplementary.pdf]

brain sciences

MDPI

Article

Towards the Development of a Deep Learning Framework Using Adaptive and Non-Adaptive Time-Frequency Features for EEG-Based Depression Therapy Prediction

Hesam Akbari [1], Sara Bagherzadeh [2], Javid Farhadi Sedehi [2], Rab Nawaz [3,*], Reza Rostami [4], Reza Kazemi [4], Sadiq Muhammad [5,*], Haihua Chen [6] and Mutlu Mete [1]

1 Department of Information Science, University of North Texas, Denton, TX 76205, USA; hesam.akbari@unt.edu (H.A.); mutlu.mete@unt.edu (M.M.)
2 Department of Biomedical Engineering, Science and Research Branch, Islamic Azad University, Tehran 1477893855, Iran; sara.bagherzadeh@srbiau.ac (S.B.); javid.farhadi@srbiau.ac.ir (J.F.S.)
3 School of Computer Science and Electronic Engineering, University of Essex, Colchester CO4 3SQ, UK
4 Department of Psychiatry, University of Tehran, Tehran 141556619, Iran; reza.rostami@ut.ac.ir (R.R.); reza.kazemi@ut.ac.ir (R.K.)
5 School of Computing, Gachon University, Seongnam-si 13120, Republic of Korea
6 Department of Data Science, University of North Texas, Denton, TX 76205, USA; haihua.chen@unt.edu
* Correspondence: rab.nawaz@essex.ac.uk (R.N.); 202511115.gachon@gmail.com (S.M.)

Introduction

The supplementary materials comprise 28 figures, 24 tables, and 4 subsections. Figures S1–S4 illustrate the detailed layer-by-layer architectures of the four pretrained CNN models evaluated in this study, namely ResNet-18, MobileNet-V3, EfficientNet-B0, and TinyViT-Hybrid, with accompanying descriptions of the spatial resolution evolution, block structures, and task-specific modifications applied to each model. Tables S1–S24 report the complete per-fold classification results including Accuracy, Precision, Recall, Specificity, and F1-Score across the six image-level CV folds for all combinations of four models, three time-frequency representations (CWT, VMD, and Fusion), and two treatment datasets (SSRI and rTMS), together with the mean and standard deviation to enable a transparent assessment of fold-to-fold variability and performance stability. Figures S5–S28 present the corresponding per-fold training and validation loss and accuracy curves for each model–representation–dataset combination, arranged in a 2 × 3 grid layout to allow direct visual inspection of convergence behaviour and consistency across folds. The supplementary is organised into four subsections: Subsection S1 (ResNet-18), Subsection S2 (MobileNet-V3 Large), Subsection S3 (EfficientNet-B0), and Subsection S4 (TinyViT-Hybrid), each providing the architectural description of the corresponding model, followed by Subsection S5, which consolidates all per-fold quantitative results and training dynamics across both datasets and all three time-frequency representations.

S1. ResNet-18

ResNet-18 is an 18-layer deep residual network that accepts a $224 \times 224 \times 3$ input image. The network begins with a convolutional stem consisting of a 7×7 convolution with 64 filters and stride 2, followed by max pooling, which reduces the spatial resolution to $56 \times 56 \times 64$. The backbone comprises four sequential stages, each composed of two Basic Blocks with residual (skip) connections that bypass the nonlinear transformations to facilitate gradient flow during training. The spatial resolution and channel depth evolve through the stages as follows: $56 \times 56 \times 64$ (Stage 1), $28 \times 28 \times 128$ (Stage 2), $14 \times 14 \times 256$

(Stage 3), and $7 \times 7 \times 512$ (Stage 4). Each Basic Block consists of two 3×3 convolutions, each followed by Batch Normalisation and ReLU activation, with a shortcut 1×1 convolution applied when the number of channels changes between the input and output of the block. After the final stage, global average pooling produces a 512-dimensional feature vector, which is passed to a fully connected layer. In the proposed CAD system, the original 1000-class output layer is replaced by a linear layer mapping the 512-dimensional representation to a 2-class output (responder vs. non-responder). The model is initialised with ImageNet pretrained weights and fine-tuned end-to-end. The architecture is illustrated in Figure S1.

S2. MobileNet-V3

MobileNet-V3 accepts a $224 \times 224 \times 3$ input image and is designed for efficient inference through a combination of depthwise separable convolutions and neural architecture search. The network begins with a 3×3 convolution (stride 2, 16 channels) followed by Batch Normalisation and ReLU activation, reducing the spatial resolution to $112 \times 112 \times 16$. The backbone consists of 15 Inverted Residual Blocks (also called Bottleneck blocks) that progressively expand and contract the channel dimension through expansion ratios, with spatial dimensions evolving from $112 \times 112 \times 16$ to $7 \times 7 \times 160$ across successive blocks. Each Inverted Residual Block consists of a pointwise 1×1 expansion convolution, Batch Normalisation, and Swish activation; a depthwise 3×3 convolution with Batch Normalisation and Swish activation; a Squeeze-and-Excitation (SE) attention module comprising average pooling, a 1×1 pointwise reduction convolution with ReLU, and a 1×1 re-expansion convolution with Swish; and a final pointwise 1×1 projection convolution with Batch Normalisation and Swish activation. Skip connections are applied when the input and output dimensions match. After the last Inverted Residual Block, a 1×1 convolution (stride 2, 960 channels) expands the feature map to $7 \times 7 \times 960$, followed by global average pooling and a three-layer MLP classifier head. In the proposed CAD system, the final linear layer of the classifier head is replaced by a binary output layer. The model is initialised with ImageNet pretrained weights and fine-tuned end-to-end. The architecture is illustrated in Figure S2.

S3. EfficientNet-B0

EfficientNet-B0 is the baseline model of the EfficientNet family, which applies compound scaling to jointly optimise network depth, width, and input resolution using a fixed scaling coefficient. The model accepts a $224 \times 224 \times 3$ input image. The network begins with a 3×3 convolution (stride 2, 32 channels) followed by Batch Normalisation and Swish activation, reducing the spatial resolution to $112 \times 112 \times 32$. The backbone comprises seven stages of Mobile Inverted Bottleneck (MBConv) blocks with depthwise separable convolutions of either 3×3 or 5×5 kernel size depending on the stage. Each MBConv block follows the structure: a pointwise 1×1 expansion convolution with Batch Normalisation and Swish activation; a depthwise convolution (3×3 or 5×5) with Batch Normalisation and Swish activation; an integrated Squeeze-and-Excitation (SE) module with a reduction ratio of 0.25; and a final pointwise 1×1 projection convolution with Batch Normalisation. Skip connections with stochastic depth regularisation are applied when the input and output dimensions match. The spatial dimensions and channel depths evolve progressively across blocks as: $112 \times 112 \times 16$, $56 \times 56 \times 24$, $28 \times 28 \times 40$, $14 \times 14 \times 80$, $14 \times 14 \times 112$, $7 \times 7 \times 192$, and $7 \times 7 \times 320$. A final 3×3 convolution (stride 2) expands the feature map to $7 \times 7 \times 1280$, followed by Batch Normalisation, Swish activation, and global average pooling. In the proposed CAD system, the final classification layer is replaced by a binary linear output layer. The model is initialised with ImageNet pretrained weights and fine-tuned end-to-end. The architecture is illustrated in Figure S3.

S4. TinyViT-Hybrid

TinyViT-Hybrid is a hybrid architecture that integrates a convolutional feature extractor with a compact Transformer encoder. The model accepts a $224 \times 224 \times 3$ input image. The convolutional stem consists of two successive 3×3 convolutions (stride 2, $c = 64$ and $c = 96$ channels respectively), followed by Batch Normalisation and GELU activation, reducing the spatial resolution to $56 \times 56 \times 96$. The backbone is organised into four stages of Transformer Blocks with interleaved downsampling convolutions. Stage 1 contains two Transformer Blocks followed by a 3×3 convolution (stride 2, $c = 192$), outputting $28 \times 28 \times 192$. Stage 2 contains two Transformer Blocks followed by a 3×3 convolution (stride 2, $c = 384$), outputting $14 \times 14 \times 384$. Stage 3 contains four Transformer Blocks followed by a 3×3 convolution (stride 2, $c = 768$), outputting $7 \times 7 \times 768$. Stage 4 contains two Transformer Blocks, preserving the $7 \times 7 \times 768$ spatial resolution. Each Transformer Block consists of Layer Normalisation, Local Window Multi-Head Self-Attention (W-MSA) with residual connection, a second Layer Normalisation, and a Multi-Layer Perceptron (MLP) with residual connection. The local window attention mechanism restricts self-attention computation to non-overlapping spatial windows, reducing the quadratic complexity of full self-attention while preserving local context modelling. After Stage 4, Layer Normalisation is applied, followed by global average pooling and a fully connected classification head with Softmax. In the proposed CAD system, the ResNet-18 convolutional backbone (up to `layer4`) replaces the convolutional stem and staged downsampling, producing $7 \times 7 \times 512$ feature maps that are reshaped into 49 tokens of dimension 512 and passed to a two-layer Multi-Head Self-Attention Transformer encoder ($H = 8$ heads, MLP ratio = 4, dropout = 0.1), followed by mean pooling and a binary linear classification head. The convolutional backbone is initialised with ImageNet pretrained weights and fine-tuned end-to-end. The architecture is illustrated in Figure S4.

Table S1. Per-fold image-level classification results for EfficientNet-B0 with CWT representation on the SSRI dataset.

Fold	Accuracy (%)	Precision (%)	Recall (%)	Specificity (%)	F1-Score (%)
1	97.39	96.36	97.04	97.61	96.70
2	97.89	96.67	98.03	97.80	97.34
3	97.50	95.86	97.88	97.25	96.86
4	97.83	97.99	96.47	98.72	97.23
5	98.55	98.44	97.88	98.99	98.16
6	98.39	98.30	97.60	98.90	97.95
**Mean ± Std**	97.92 ± 0.47	97.27 ± 1.11	97.49 ± 0.61	98.21 ± 0.75	97.37 ± 0.58

Table S2. Per-fold image-level classification results for MobileNet-V3 with CWT representation on the SSRI dataset.

Fold	Accuracy (%)	Precision (%)	Recall (%)	Specificity (%)	F1-Score (%)
1	97.50	97.30	96.34	98.26	96.82
2	98.44	97.49	98.59	98.35	98.04
3	97.67	96.13	98.03	97.43	97.07
4	98.50	98.03	98.17	98.72	98.10
5	98.67	99.28	97.32	99.54	98.29
6	98.61	98.86	97.60	99.27	98.23
**Mean ± Std**	98.23 ± 0.51	97.85 ± 1.14	97.67 ± 0.79	98.59 ± 0.76	97.76 ± 0.64

Table S3. Per-fold image-level classification results for ResNet-18 with CWT representation on the SSRI dataset.

Fold	Accuracy (%)	Precision (%)	Recall (%)	Specificity (%)	F1-Score (%)
1	99.17	98.87	99.01	99.27	98.94
2	99.39	99.57	98.87	99.72	99.22
3	99.44	98.88	99.72	99.27	99.30
4	99.61	100.00	99.01	100.00	99.50
5	99.56	99.16	99.72	99.45	99.44
6	99.44	99.72	98.87	99.82	99.29
**Mean ± Std**	99.43 ± 0.15	99.37 ± 0.47	99.20 ± 0.41	99.59 ± 0.31	99.28 ± 0.20

Table S4. Per-fold image-level classification results for TinyViT-Hybrid with CWT representation on the SSRI dataset.

Fold	Accuracy (%)	Precision (%)	Recall (%)	Specificity (%)	F1-Score (%)
1	99.11	98.46	99.30	98.99	98.88
2	99.28	99.57	98.59	99.72	99.08
3	99.06	98.46	99.15	98.99	98.81
4	99.33	99.43	98.87	99.63	99.15
5	99.33	99.43	98.87	99.63	99.15
6	99.56	99.58	99.29	99.72	99.44
**Mean ± Std**	99.28 ± 0.18	99.16 ± 0.54	99.01 ± 0.28	99.45 ± 0.36	99.08 ± 0.22

Table S5. Per-fold image-level classification results for EfficientNet-B0 with VMD representation on the SSRI dataset.

Fold	Accuracy (%)	Precision (%)	Recall (%)	Specificity (%)	F1-Score (%)
1	96.44	94.61	96.48	96.42	95.54
2	97.17	96.47	96.34	97.70	96.41
3	96.05	92.76	97.60	95.05	95.12
4	97.50	97.16	96.47	98.17	96.82
5	97.16	97.00	95.77	98.07	96.38
6	96.16	94.32	96.05	96.24	95.18
**Mean ± Std**	96.75 ± 0.60	95.39 ± 1.76	96.45 ± 0.63	96.94 ± 1.24	95.91 ± 0.72

Table S6. Per-fold image-level classification results for MobileNet-V3 with VMD representation on the SSRI dataset.

Fold	Accuracy (%)	Precision (%)	Recall (%)	Specificity (%)	F1-Score (%)
1	97.28	96.48	96.62	97.70	96.55
2	96.39	94.98	95.92	96.69	95.44
3	97.39	95.97	97.46	97.34	96.71
4	97.00	96.32	96.05	97.61	96.19
5	97.50	97.03	96.61	98.07	96.82
6	97.61	98.83	95.06	99.27	96.91
**Mean ± Std**	97.19 ± 0.45	96.60 ± 1.28	96.29 ± 0.81	97.78 ± 0.86	96.44 ± 0.55

Table S7. Per-fold image-level classification results for ResNet-18 with VMD representation on the SSRI dataset.

Fold	Accuracy (%)	Precision (%)	Recall (%)	Specificity (%)	F1-Score (%)
1	98.83	99.00	98.03	99.36	98.51
2	98.67	97.64	99.01	98.44	98.32
3	98.72	98.17	98.59	98.81	98.38
4	98.78	98.18	98.73	98.81	98.45
5	99.22	98.87	99.15	99.27	99.01
6	98.83	99.14	97.88	99.45	98.51
**Mean ± Std**	98.84 ± 0.20	98.50 ± 0.59	98.57 ± 0.52	99.02 ± 0.40	98.53 ± 0.25

Table S8. Per-fold image-level classification results for TinyViT-Hybrid with VMD representation on the SSRI dataset.

Fold	Accuracy (%)	Precision (%)	Recall (%)	Specificity (%)	F1-Score (%)
1	98.28	98.43	97.18	98.99	97.80
2	98.83	99.00	98.03	99.36	98.51
3	98.50	98.16	98.03	98.81	98.09
4	98.83	98.59	98.45	99.08	98.52
5	98.83	98.59	98.45	99.08	98.52
6	99.05	99.29	98.31	99.54	98.80
**Mean ± Std**	98.72 ± 0.28	98.68 ± 0.40	98.07 ± 0.48	99.14 ± 0.26	98.37 ± 0.36

Table S9. Per-fold image-level classification results for EfficientNet-B0 with Fusion representation on the SSRI dataset.

Fold	Accuracy (%)	Precision (%)	Recall (%)	Specificity (%)	F1-Score (%)
1	96.55	93.67	97.89	95.68	95.73
2	96.94	94.92	97.46	96.60	96.18
3	96.66	95.26	96.33	96.88	95.79
4	97.11	94.82	98.03	96.51	96.39
5	97.72	96.91	97.32	97.98	97.11
6	97.50	95.98	97.74	97.34	96.86
**Mean ± Std**	97.08 ± 0.46	95.26 ± 1.10	97.46 ± 0.61	96.83 ± 0.78	96.34 ± 0.56

Table S10. Per-fold image-level classification results for MobileNet-V3 with Fusion representation on the SSRI dataset.

Fold	Accuracy (%)	Precision (%)	Recall (%)	Specificity (%)	F1-Score (%)
1	97.39	96.23	97.18	97.52	96.71
2	96.83	95.16	96.90	96.79	96.02
3	96.66	94.27	97.46	96.15	95.84
4	97.33	96.75	96.47	97.89	96.61
5	97.61	96.90	97.04	97.98	96.97
6	97.44	97.02	96.47	98.07	96.75
**Mean ± Std**	97.21 ± 0.37	96.06 ± 1.11	96.92 ± 0.39	97.40 ± 0.77	96.48 ± 0.45

Table S11. Per-fold image-level classification results for ResNet-18 with Fusion representation on the SSRI dataset.

Fold	Accuracy (%)	Precision (%)	Recall (%)	Specificity (%)	F1-Score (%)
1	98.89	98.32	98.87	98.90	98.60
2	99.11	99.15	98.59	99.45	98.87
3	98.78	97.51	99.44	98.35	98.46
4	98.89	98.87	98.31	99.27	98.59
5	98.94	98.73	98.59	99.17	98.66
6	99.50	99.72	99.01	99.82	99.36
**Mean ± Std**	99.02 ± 0.26	98.71 ± 0.75	98.80 ± 0.40	99.16 ± 0.50	98.76 ± 0.33

Table S12. Per-fold image-level classification results for TinyViT-Hybrid with Fusion representation on the SSRI dataset.

Fold	Accuracy (%)	Precision (%)	Recall (%)	Specificity (%)	F1-Score (%)
1	98.44	97.89	98.17	98.62	98.03
2	98.50	97.63	98.59	98.44	98.11
3	98.50	98.16	98.03	98.81	98.09
4	99.00	98.32	99.15	98.90	98.74
5	99.28	99.29	98.87	99.54	99.08
6	99.22	99.71	98.31	99.82	99.01
**Mean ± Std**	98.82 ± 0.39	98.50 ± 0.82	98.52 ± 0.43	99.02 ± 0.54	98.51 ± 0.49

Table S13. Per-fold image-level classification results for EfficientNet-B0 with CWT representation on the rTMS dataset.

Fold	Accuracy (%)	Precision (%)	Recall (%)	Specificity (%)	F1-Score (%)
1	95.83	95.88	95.95	95.70	95.92
2	96.72	96.55	97.04	96.38	96.79
3	96.42	96.33	96.68	96.16	96.50
4	97.23	97.05	97.54	96.91	97.30
5	96.20	94.95	97.76	94.57	96.33
6	96.49	97.21	95.88	97.14	96.54
**Mean ± Std**	96.48 ± 0.47	96.33 ± 0.83	96.81 ± 0.79	96.14 ± 0.93	96.56 ± 0.46

Table S14. Per-fold image-level classification results for MobileNet-V3 with CWT representation on the rTMS dataset.

Fold	Accuracy (%)	Precision (%)	Recall (%)	Specificity (%)	F1-Score (%)
1	96.75	97.02	96.60	96.91	96.81
2	96.75	96.22	97.47	96.01	96.84
3	96.64	96.21	97.25	96.01	96.73
4	97.05	96.24	98.05	96.01	97.14
5	96.86	96.69	97.18	96.53	96.94
6	97.31	97.81	96.89	97.74	97.35
**Mean ± Std**	96.90 ± 0.24	96.70 ± 0.64	97.24 ± 0.50	96.53 ± 0.70	96.97 ± 0.23

Table S15. Per-fold image-level classification results for ResNet-18 with CWT representation on the rTMS dataset.

Fold	Accuracy (%)	Precision (%)	Recall (%)	Specificity (%)	F1-Score (%)
1	98.27	97.65	98.99	97.51	98.31
2	99.11	99.13	99.13	99.10	99.13
3	98.38	98.55	98.27	98.49	98.41
4	98.82	99.34	98.34	99.32	98.84
5	98.30	98.06	98.63	97.96	98.34
6	99.00	99.20	98.84	99.17	99.02
**Mean ± Std**	98.65 ± 0.38	98.66 ± 0.69	98.70 ± 0.35	98.59 ± 0.73	98.68 ± 0.37

Table S16. Per-fold image-level classification results for TinyViT-Hybrid with CWT representation on the rTMS dataset.

Fold	Accuracy (%)	Precision (%)	Recall (%)	Specificity (%)	F1-Score (%)
1	98.05	98.33	97.83	98.27	98.08
2	98.27	97.85	98.77	97.74	98.31
3	98.56	98.49	98.70	98.42	98.59
4	98.52	97.93	99.21	97.81	98.56
5	98.60	98.14	99.13	98.04	98.63
6	98.38	98.62	98.19	98.57	98.41
**Mean ± Std**	98.40 ± 0.21	98.23 ± 0.31	98.64 ± 0.54	98.14 ± 0.33	98.43 ± 0.21

Table S17. Per-fold image-level classification results for EfficientNet-B0 with VMD representation on the rTMS dataset.

Fold	Accuracy (%)	Precision (%)	Recall (%)	Specificity (%)	F1-Score (%)
1	97.16	97.74	96.68	97.66	97.20
2	97.23	97.26	97.33	97.14	97.29
3	96.31	96.86	95.88	96.76	96.37
4	97.01	97.38	96.75	97.29	97.06
5	96.90	96.76	97.18	96.61	96.97
6	96.64	97.22	96.17	97.14	96.69
**Mean ± Std**	96.88 ± 0.35	97.20 ± 0.35	96.66 ± 0.56	97.10 ± 0.38	96.93 ± 0.35

Table S18. Per-fold image-level classification results for MobileNet-V3 with VMD representation on the rTMS dataset.

Fold	Accuracy (%)	Precision (%)	Recall (%)	Specificity (%)	F1-Score (%)
1	96.94	96.76	97.25	96.61	97.01
2	96.94	97.31	96.68	97.21	96.99
3	97.49	97.89	97.18	97.81	97.53
4	97.34	97.40	97.40	97.29	97.40
5	96.46	96.87	96.17	96.76	96.52
6	96.72	96.68	96.89	96.53	96.79
**Mean ± Std**	96.98 ± 0.38	97.15 ± 0.46	96.93 ± 0.45	97.04 ± 0.49	97.04 ± 0.38

Table S19. Per-fold image-level classification results for ResNet-18 with VMD representation on the rTMS dataset.

Fold	Accuracy (%)	Precision (%)	Recall (%)	Specificity (%)	F1-Score (%)
1	98.52	98.35	98.77	98.27	98.56
2	98.97	99.13	98.84	99.10	98.99
3	98.78	99.13	98.48	99.10	98.80
4	98.86	98.36	99.42	98.27	98.89
5	98.78	98.70	98.92	98.64	98.81
6	98.71	98.84	98.63	98.79	98.73
**Mean ± Std**	98.77 ± 0.15	98.75 ± 0.35	98.84 ± 0.32	98.69 ± 0.37	98.80 ± 0.15

Table S20. Per-fold image-level classification results for TinyViT-Hybrid with VMD representation on the rTMS dataset.

Fold	Accuracy (%)	Precision (%)	Recall (%)	Specificity (%)	F1-Score (%)
1	98.75	98.35	99.21	98.27	98.78
2	98.78	98.77	98.84	98.72	98.81
3	98.30	98.20	98.48	98.12	98.34
4	98.78	98.98	98.63	98.94	98.81
5	98.12	97.92	98.41	97.81	98.16
6	98.82	99.63	98.05	99.62	98.83
**Mean ± Std**	98.59 ± 0.30	98.64 ± 0.62	98.60 ± 0.40	98.58 ± 0.65	98.62 ± 0.29

Table S21. Per-fold image-level classification results for EfficientNet-B0 with Fusion representation on the rTMS dataset.

Fold	Accuracy (%)	Precision (%)	Recall (%)	Specificity (%)	F1-Score (%)
1	95.98	96.03	96.10	95.86	96.06
2	96.61	96.34	97.04	96.16	96.69
3	96.20	96.92	95.59	96.83	96.25
4	97.01	96.97	97.18	96.83	97.08
5	96.20	95.78	96.82	95.55	96.30
6	96.57	97.22	96.02	97.14	96.62
**Mean ± Std**	96.43 ± 0.37	96.54 ± 0.58	96.46 ± 0.64	96.39 ± 0.63	96.50 ± 0.37

Table S22. Per-fold image-level classification results for MobileNet-V3 with Fusion representation on the rTMS dataset.

Fold	Accuracy (%)	Precision (%)	Recall (%)	Specificity (%)	F1-Score (%)
1	97.64	97.28	98.12	97.14	97.70
2	96.75	96.15	97.54	95.93	96.84
3	96.79	97.10	96.60	96.99	96.85
4	97.75	97.42	98.19	97.29	97.81
5	97.01	96.30	97.90	96.08	97.10
6	96.49	96.73	96.38	96.61	96.56
**Mean ± Std**	97.07 ± 0.51	96.83 ± 0.52	97.46 ± 0.78	96.67 ± 0.57	97.14 ± 0.50

Table S23. Per-fold image-level classification results for ResNet-18 with Fusion representation on the rTMS dataset.

Fold	Accuracy (%)	Precision (%)	Recall (%)	Specificity (%)	F1-Score (%)
1	98.34	98.34	98.41	98.27	98.37
2	98.60	98.07	99.21	97.97	98.64
3	98.38	99.26	97.54	99.25	98.40
4	98.64	98.98	98.34	98.94	98.66
5	98.38	98.62	98.19	98.57	98.41
6	98.60	98.63	98.63	98.57	98.63
**Mean ± Std**	98.49 ± 0.14	98.65 ± 0.43	98.39 ± 0.54	98.59 ± 0.46	98.52 ± 0.14

Table S24. Per-fold image-level classification results for TinyViT-Hybrid with Fusion representation on the rTMS dataset.

Fold	Accuracy (%)	Precision (%)	Recall (%)	Specificity (%)	F1-Score (%)
1	98.52	98.35	98.77	98.27	98.56
2	98.30	98.27	98.41	98.19	98.34
3	98.23	98.06	98.48	97.97	98.27
4	99.04	99.27	98.84	99.25	99.06
5	98.38	97.79	99.06	97.66	98.42
6	98.75	99.20	98.34	99.17	98.77
**Mean ± Std**	98.54 ± 0.31	98.49 ± 0.61	98.65 ± 0.28	98.42 ± 0.65	98.57 ± 0.30

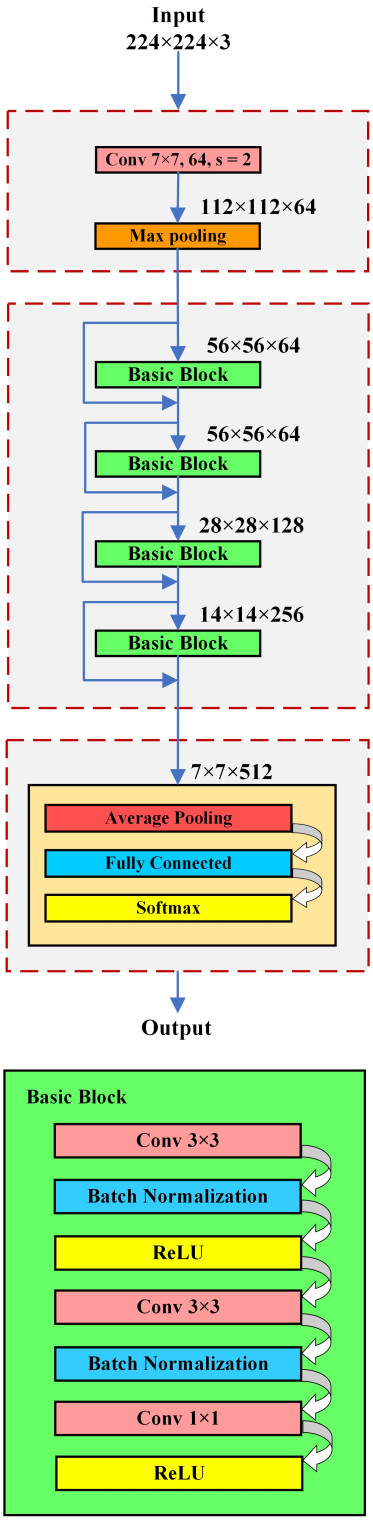

Figure S1. Architecture diagram of ResNet-18.

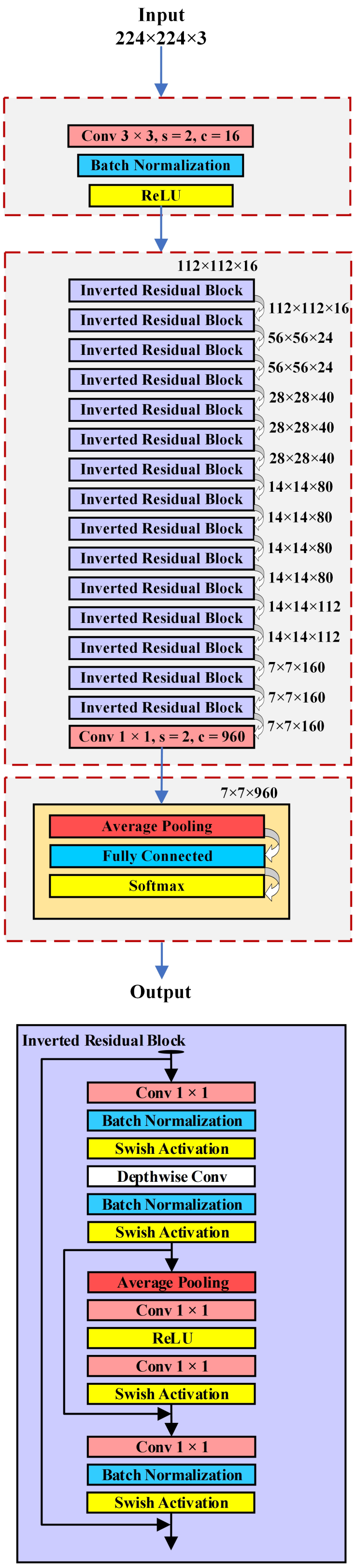

Figure S2. Architecture diagram of MobileNet-V3.

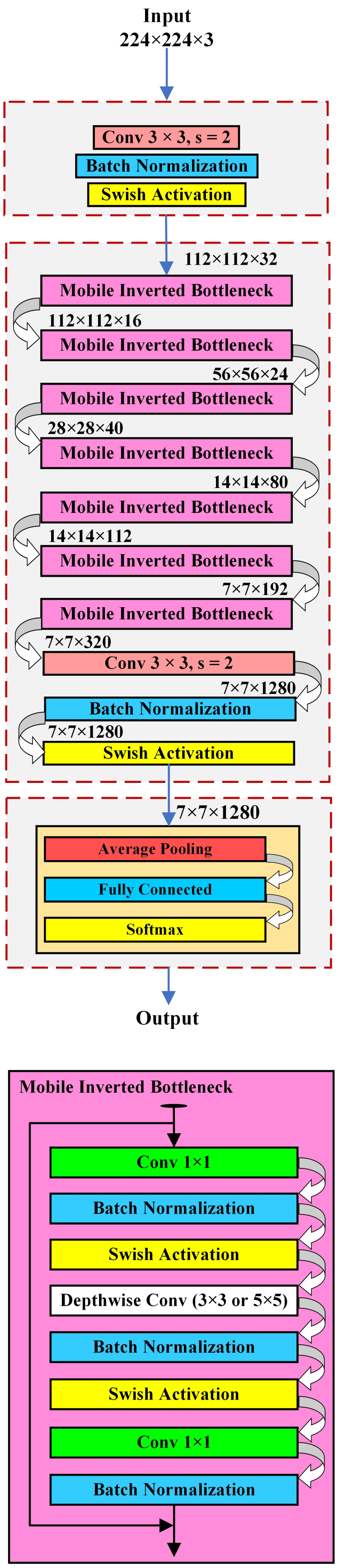

Figure S3. Architecture diagram of EfficientNet-B0.

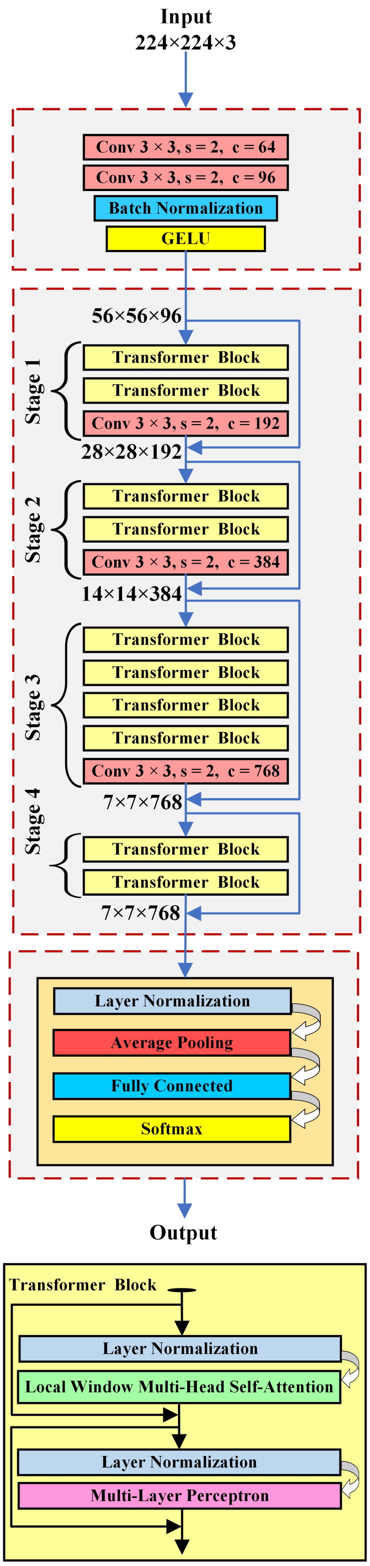

Figure S4. Architecture diagram of TinyViT-Hybrid.

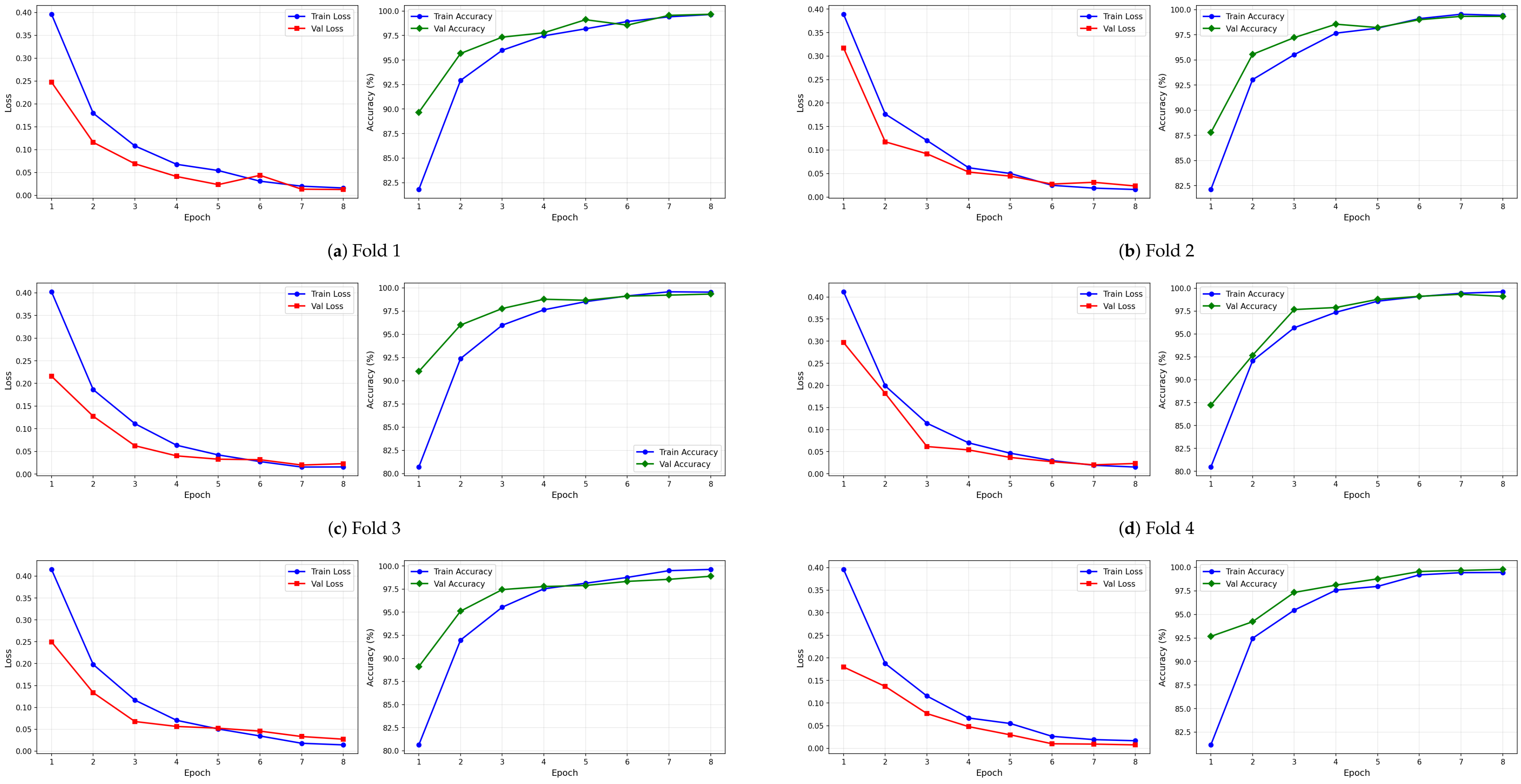

(**a**) Fold 1 (**b**) Fold 2
(**c**) Fold 3 (**d**) Fold 4
(**e**) Fold 5 (**f**) Fold 6

Figure S5. Per-fold training and validation loss and accuracy curves for ResNet-18 with CWT representation on the SSRI dataset.

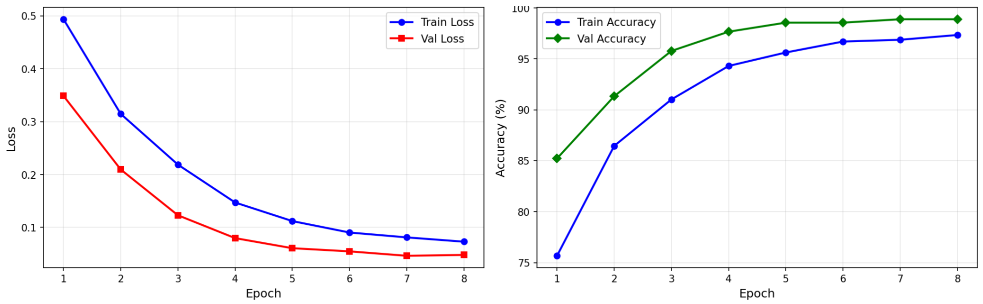

(**a**) Fold 1

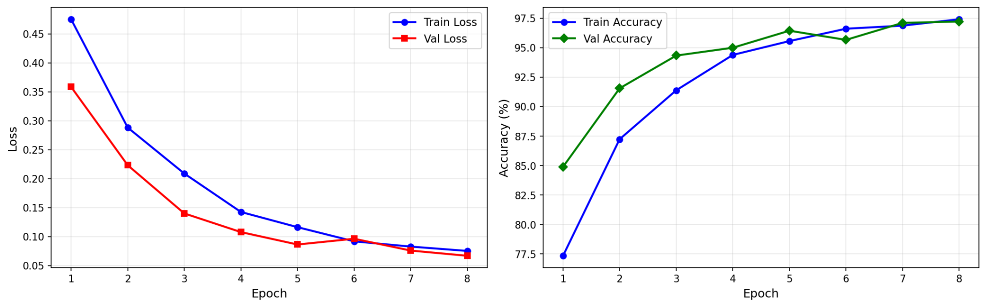

(**b**) Fold 2

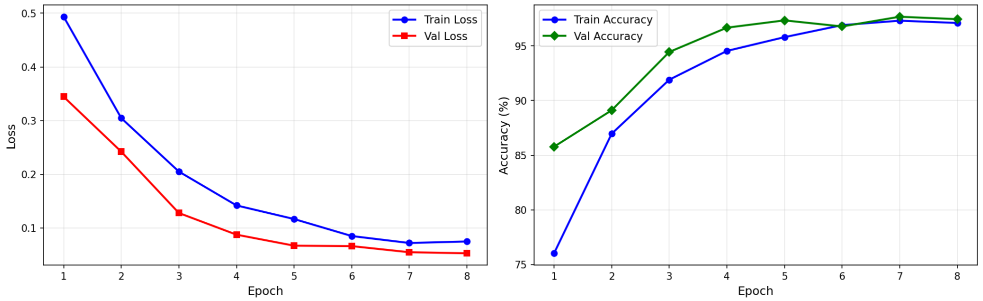

(**c**) Fold 3

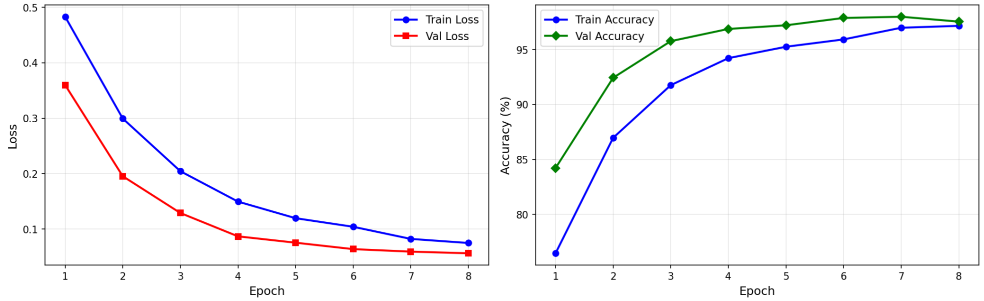

(**d**) Fold 4

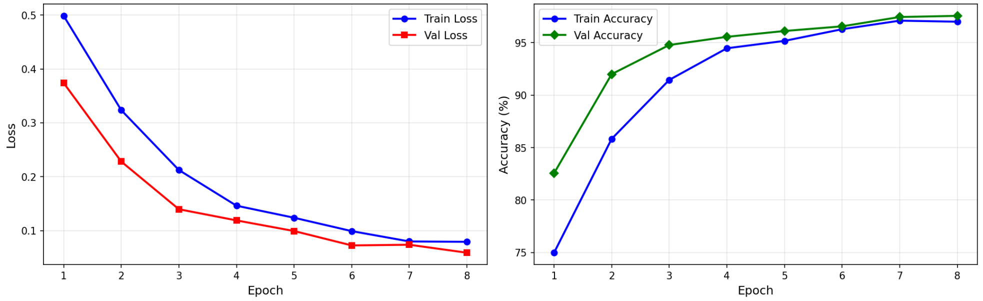

(**e**) Fold 5

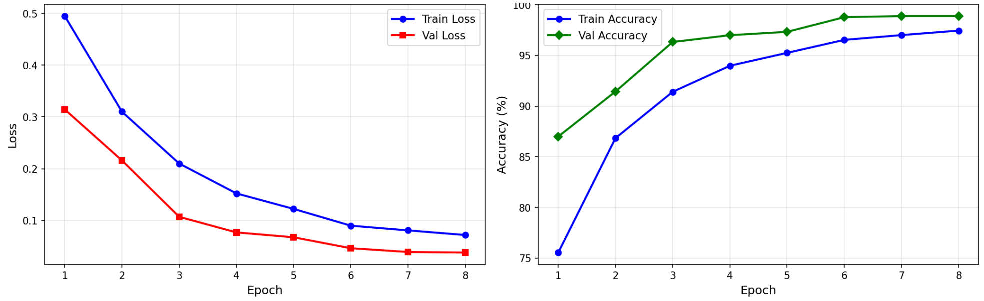

(**f**) Fold 6

Figure S6. Per-fold training and validation loss and accuracy curves for EfficientNet-B0 with CWT representation on the SSRI dataset.

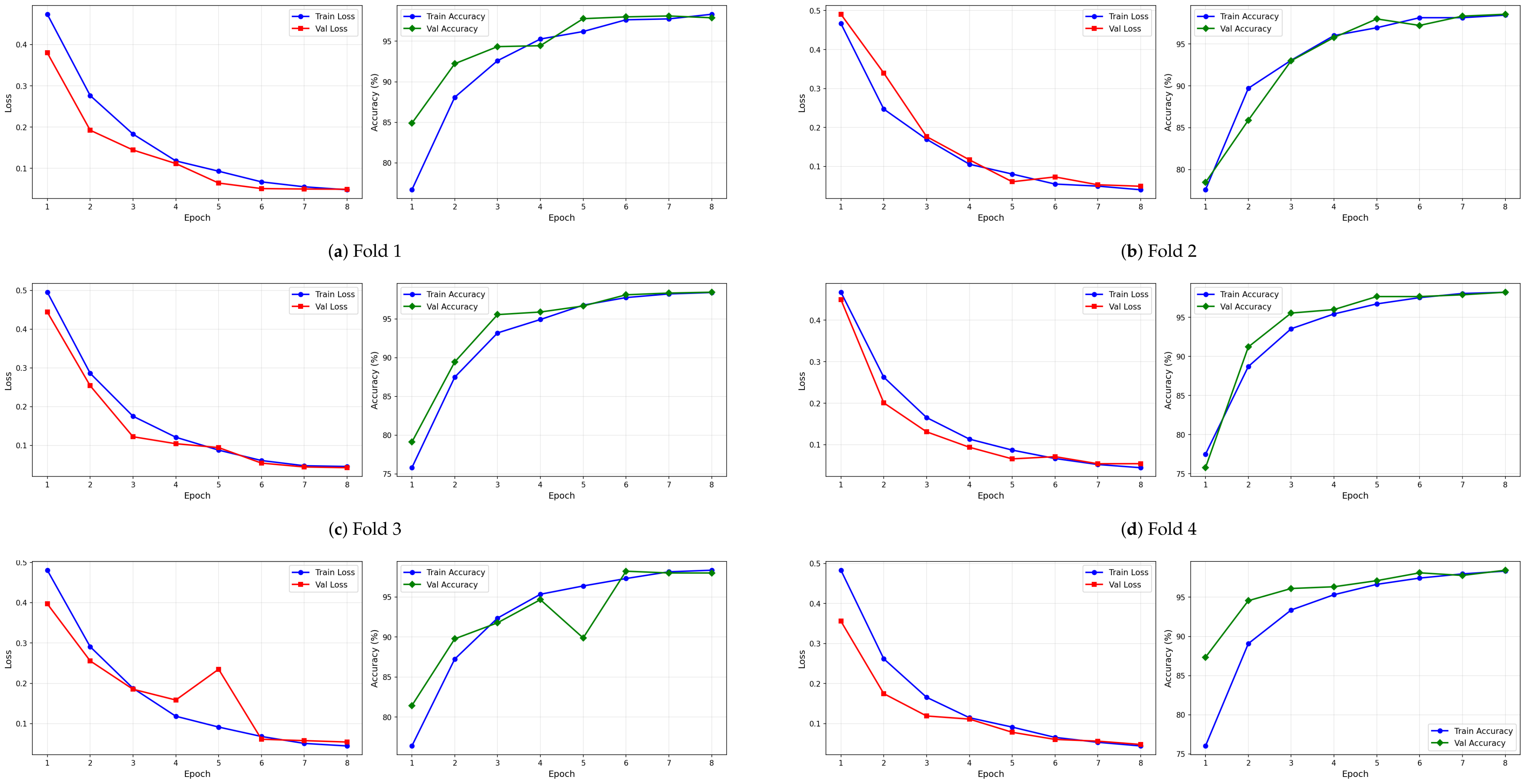

(**a**) Fold 1 (**b**) Fold 2

(**c**) Fold 3 (**d**) Fold 4

(**e**) Fold 5 (**f**) Fold 6

Figure S7. Per-fold training and validation loss and accuracy curves for MobileNet-V3 with CWT representation on the SSRI dataset.

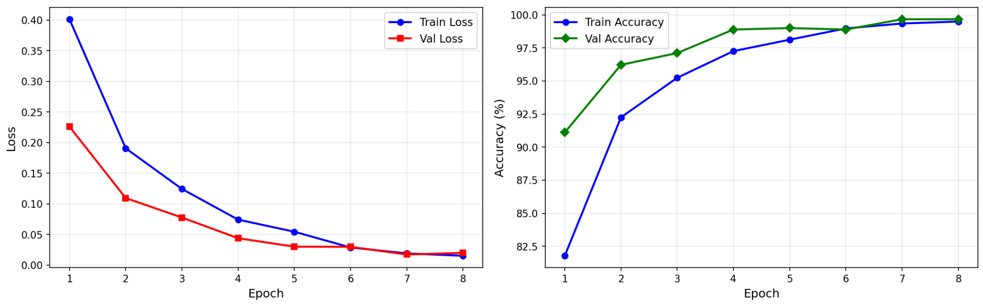

(**a**) Fold 1

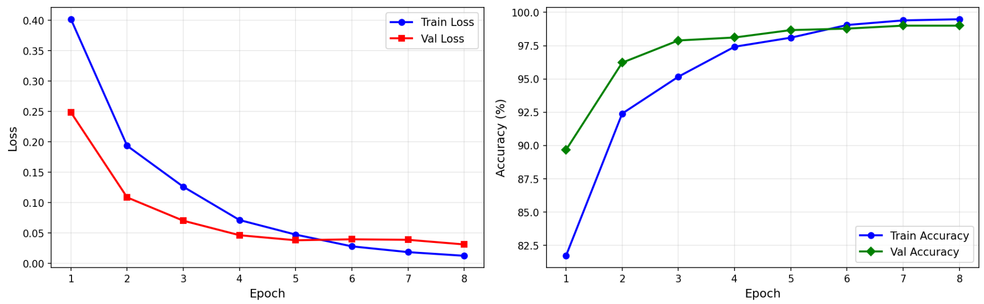

(**b**) Fold 2

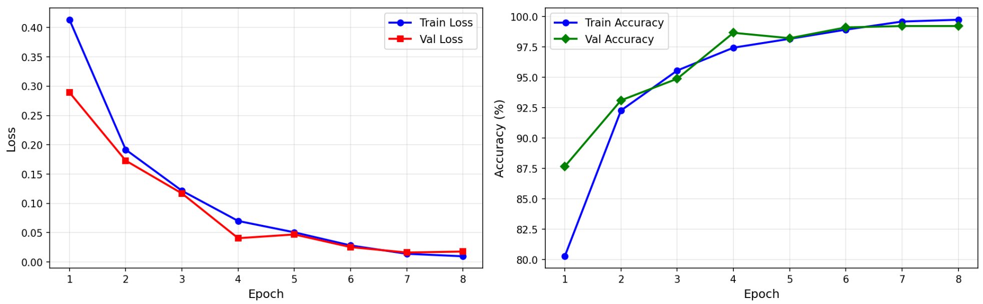

(**c**) Fold 3

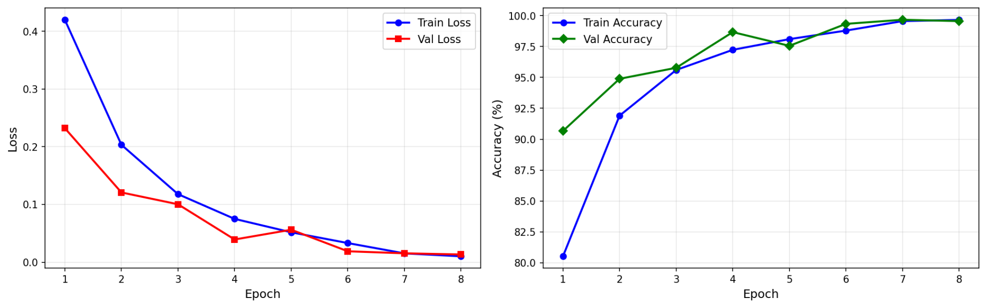

(**d**) Fold 4

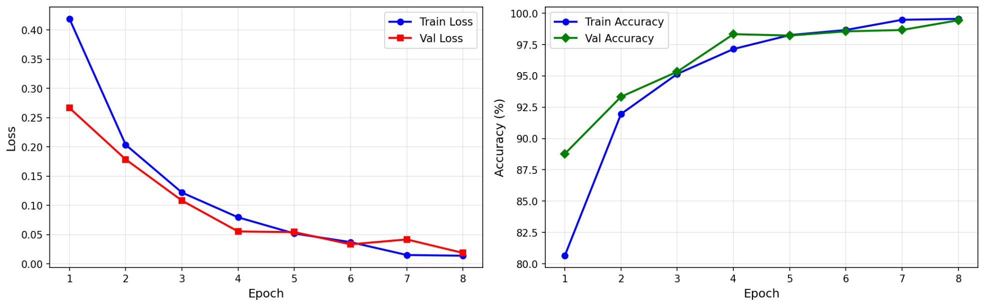

(**e**) Fold 5

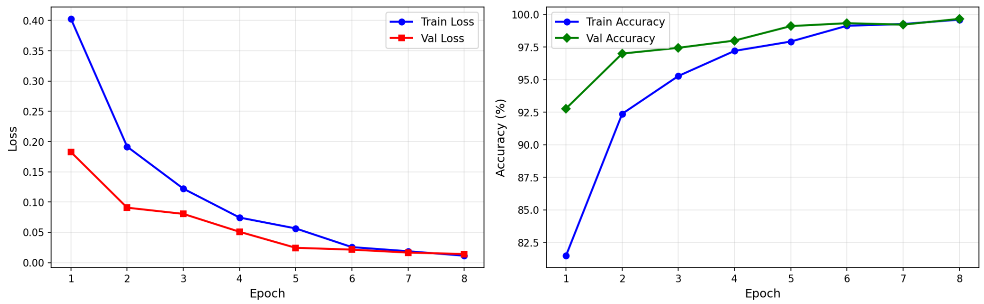

(**f**) Fold 6

Figure S8. Per-fold training and validation loss and accuracy curves for TinyViT-Hybrid with CWT representation on the SSRI dataset.

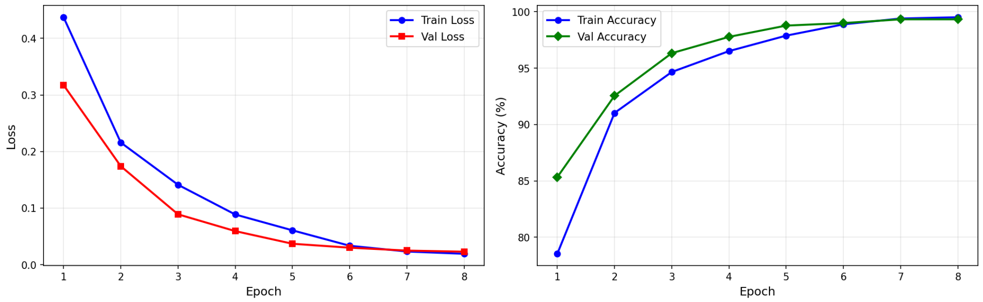

(**a**) Fold 1

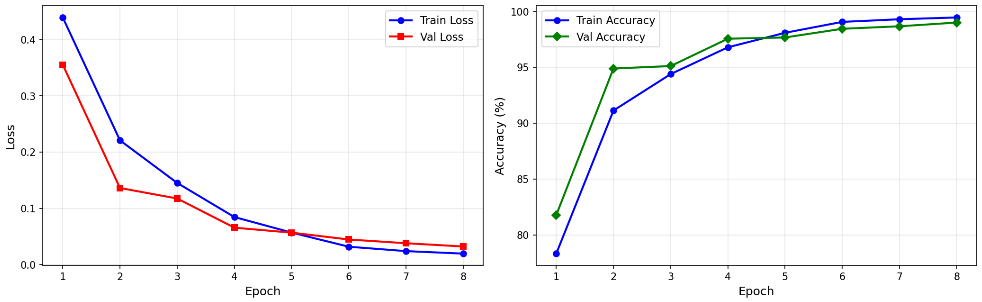

(**b**) Fold 2

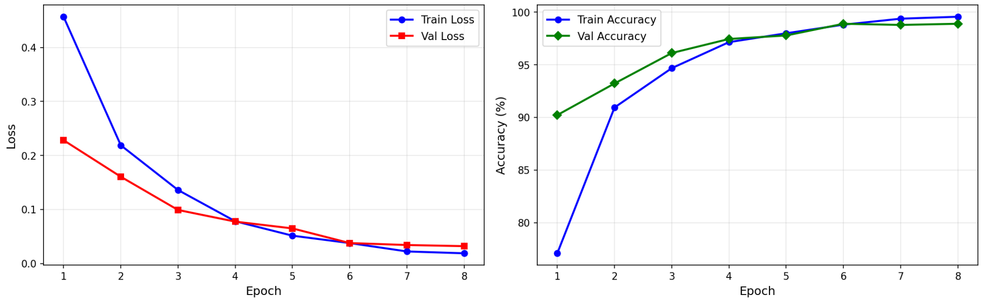

(**c**) Fold 3

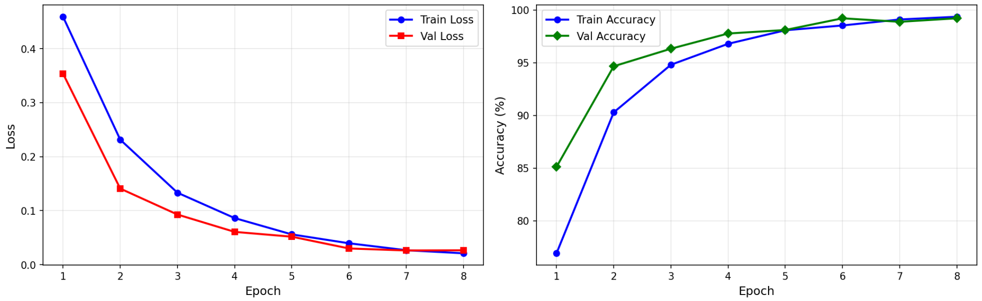

(**d**) Fold 4

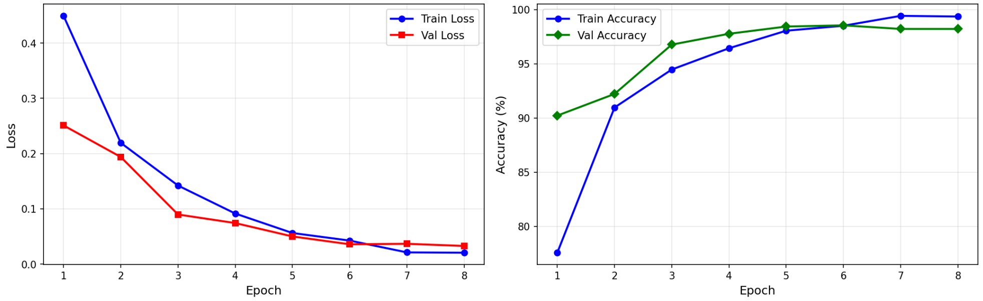

(**e**) Fold 5

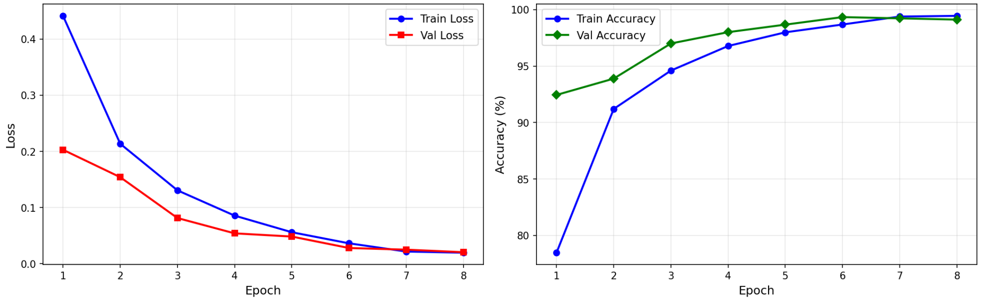

(**f**) Fold 6

Figure S9. Per-fold training and validation loss and accuracy curves for ResNet-18 with VMD representation on the SSRI dataset.

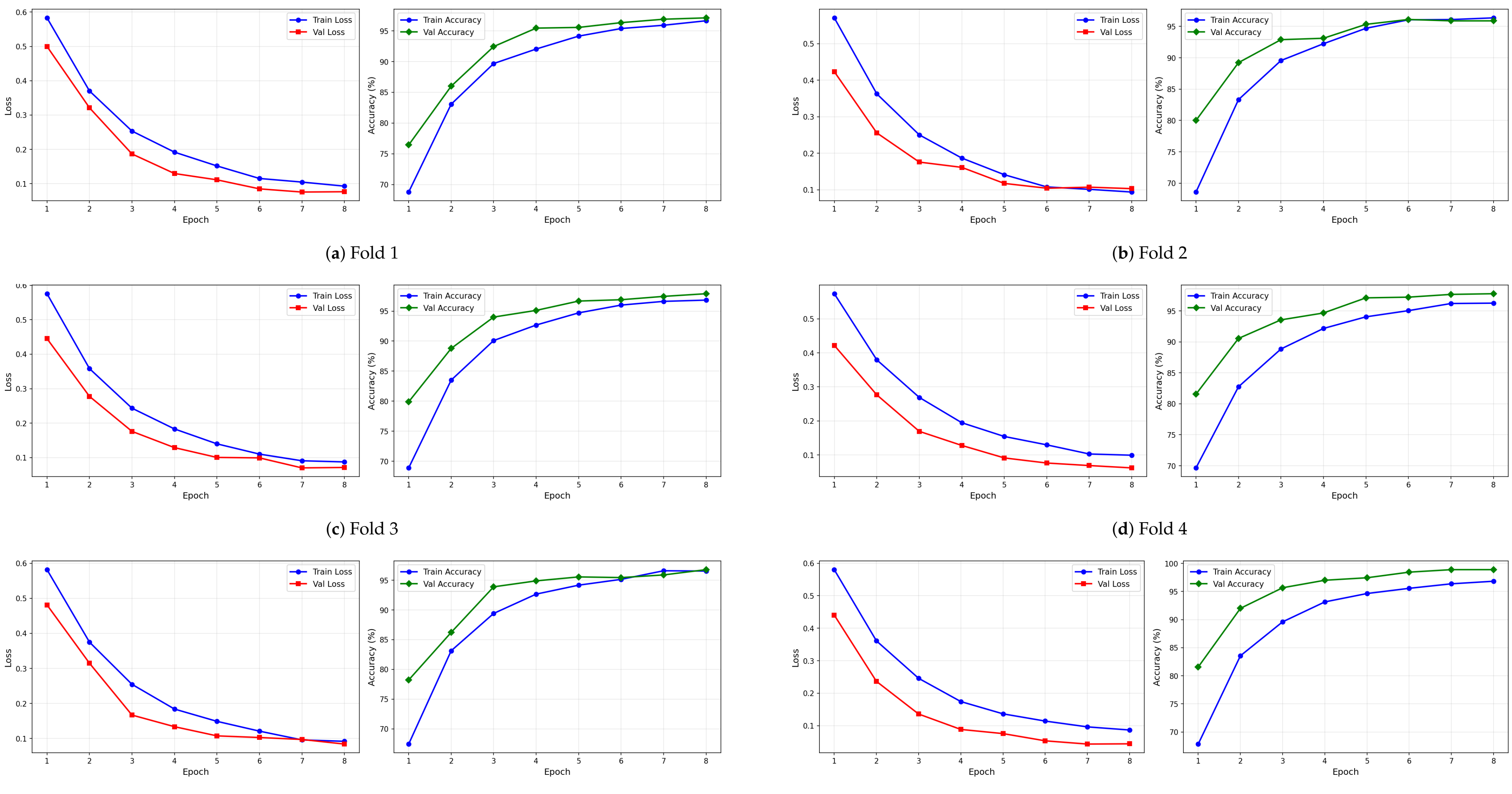

(**a**) Fold 1 (**b**) Fold 2
(**c**) Fold 3 (**d**) Fold 4
(**e**) Fold 5 (**f**) Fold 6

Figure S10. Per-fold training and validation loss and accuracy curves for EfficientNet-B0 with VMD representation on the SSRI dataset.

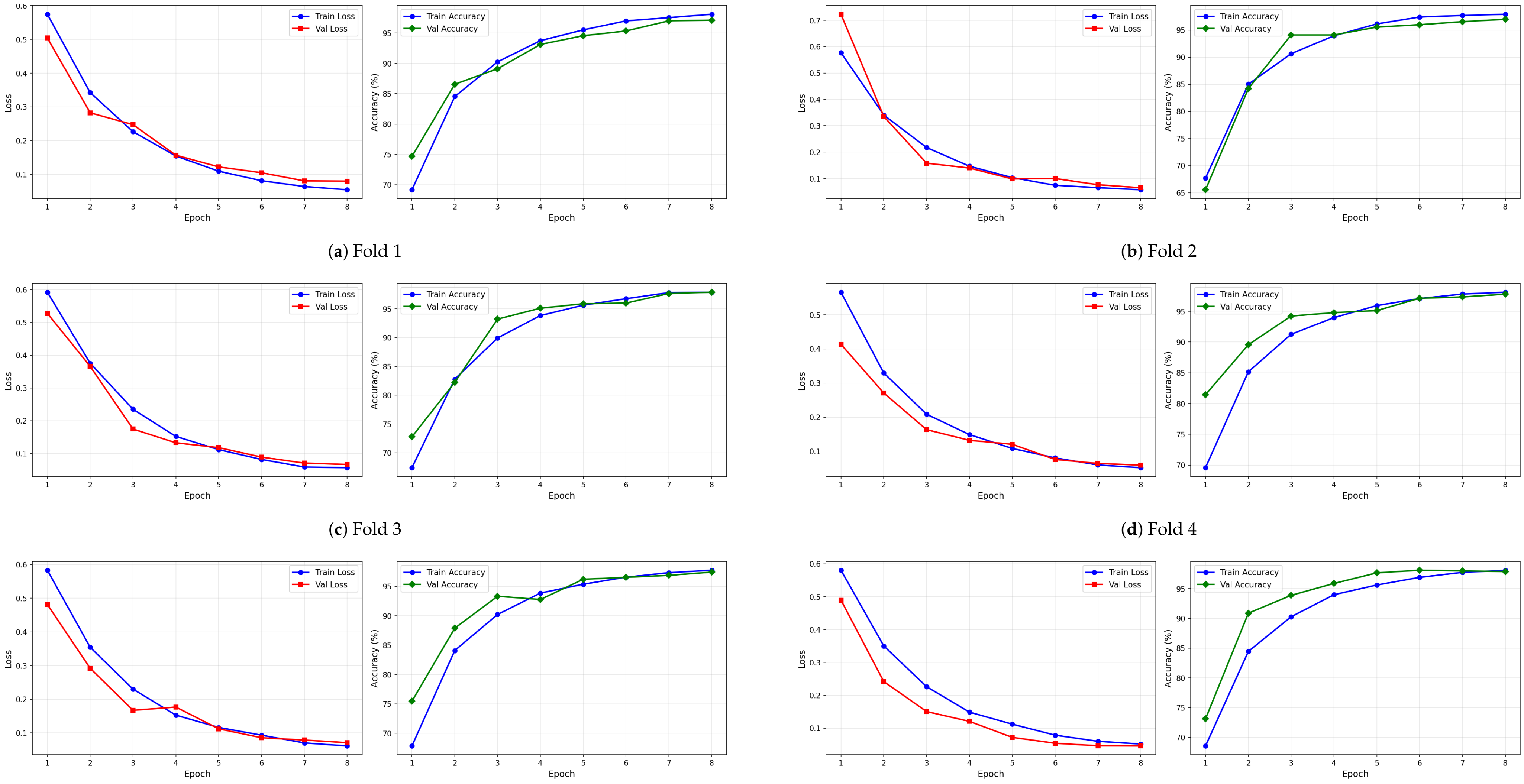

(**a**) Fold 1 (**b**) Fold 2

(**c**) Fold 3 (**d**) Fold 4

(**e**) Fold 5 (**f**) Fold 6

Figure S11. Per-fold training and validation loss and accuracy curves for MobileNet-V3 with VMD representation on the SSRI dataset.

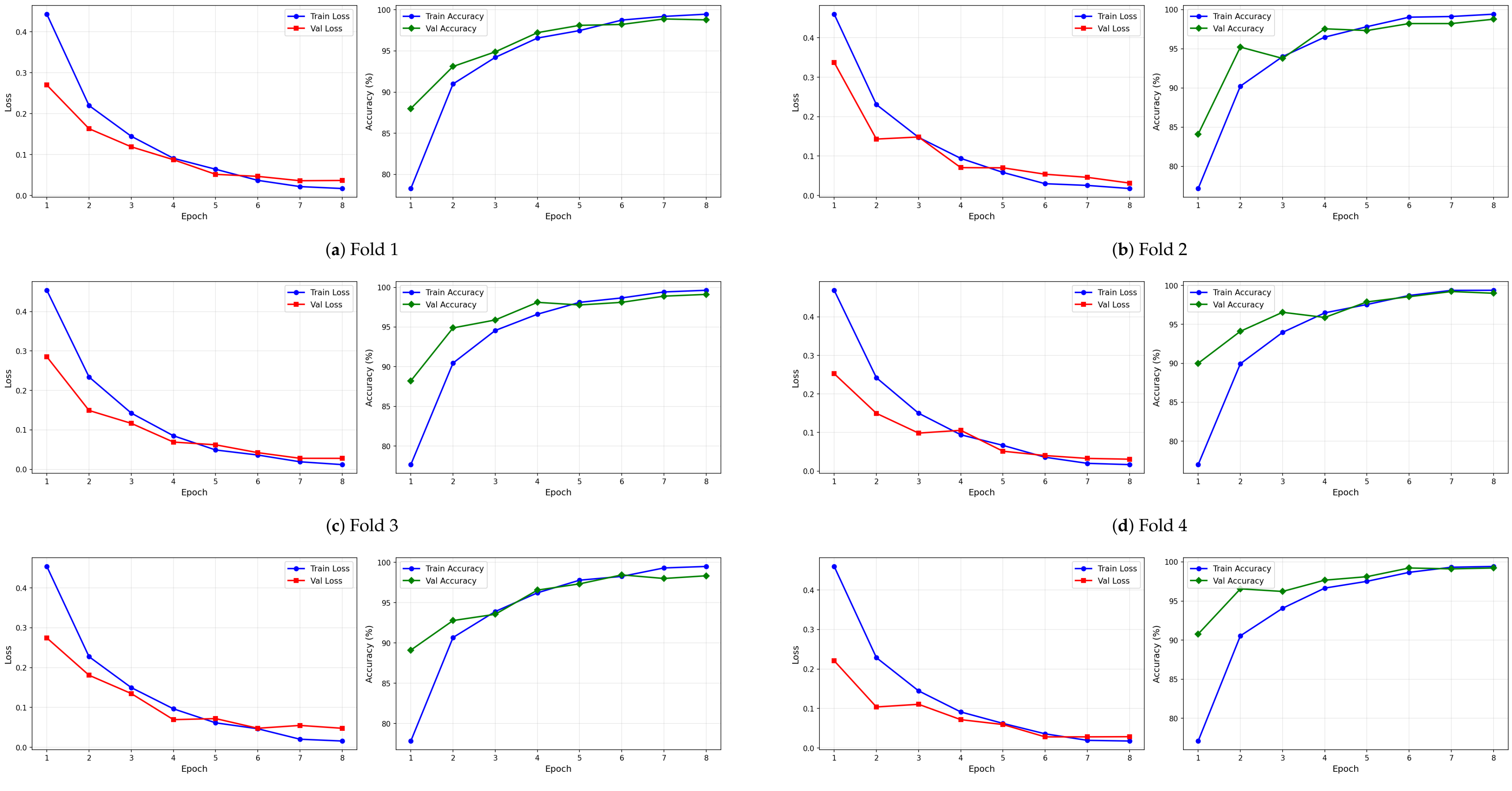

(**a**) Fold 1 (**b**) Fold 2

(**c**) Fold 3 (**d**) Fold 4

(**e**) Fold 5 (**f**) Fold 6

Figure S12. Per-fold training and validation loss and accuracy curves for TinyViT-Hybrid with VMD representation on the SSRI dataset.

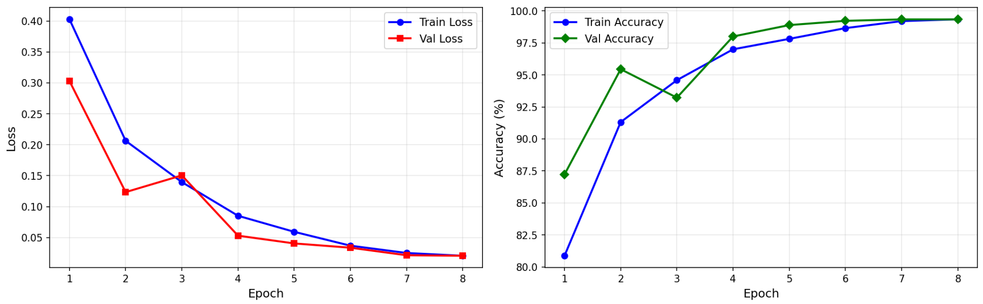

(**a**) Fold 1

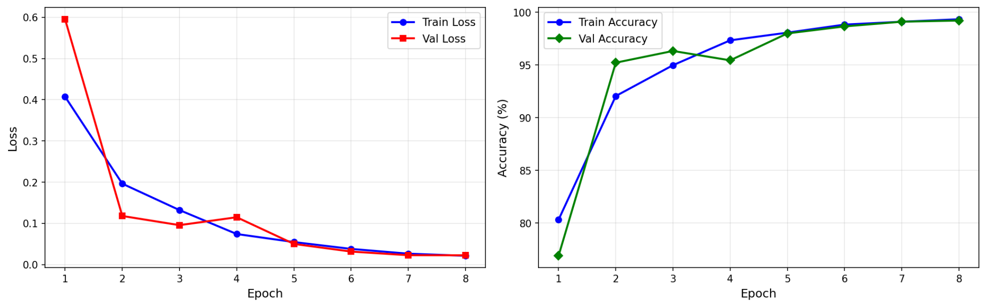

(**b**) Fold 2

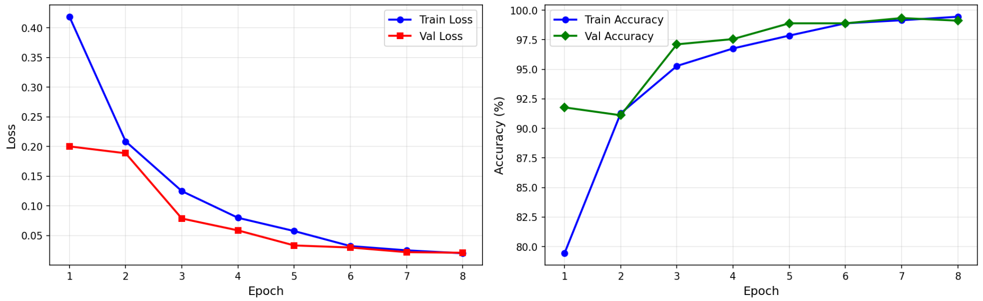

(**c**) Fold 3

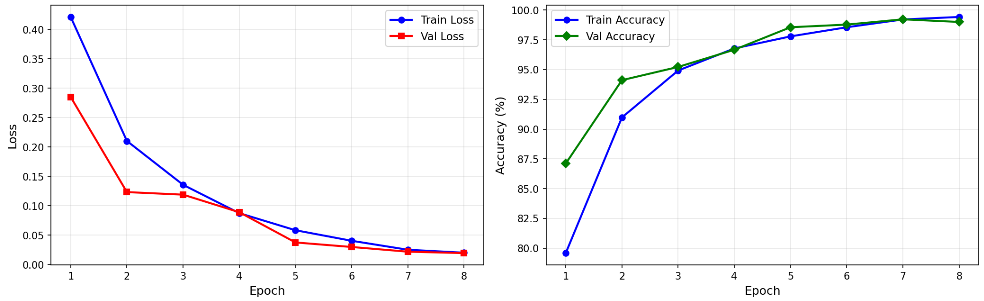

(**d**) Fold 4

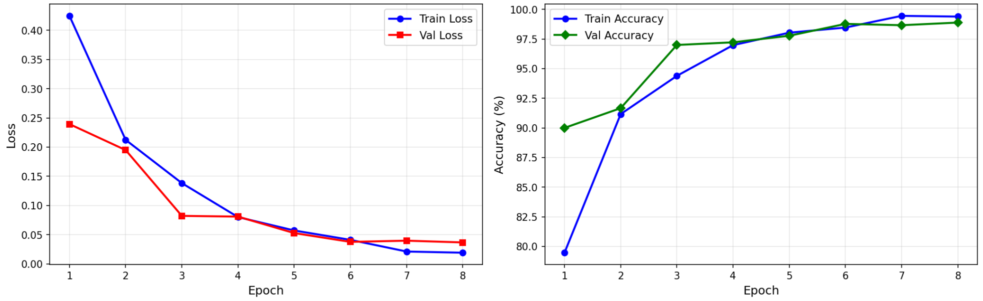

(**e**) Fold 5

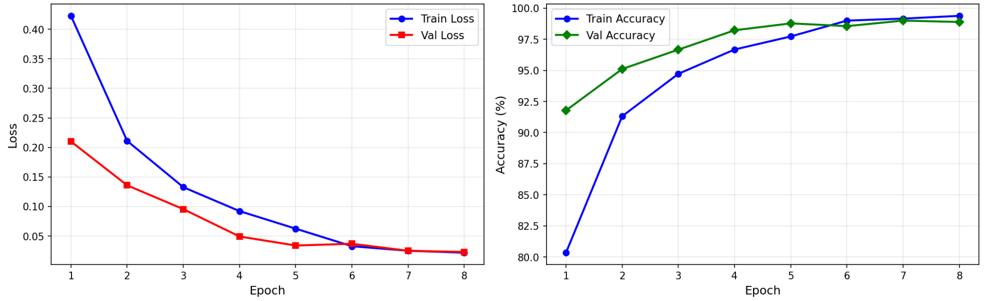

(**f**) Fold 6

Figure S13. Per-fold training and validation loss and accuracy curves for ResNet-18 with Fusion representation on the SSRI dataset.

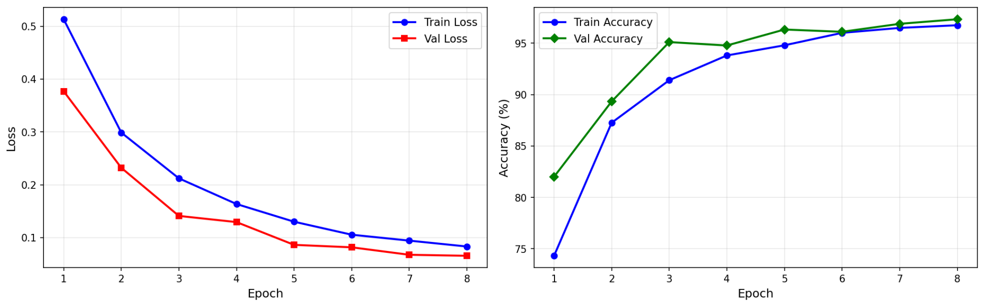

(**a**) Fold 1

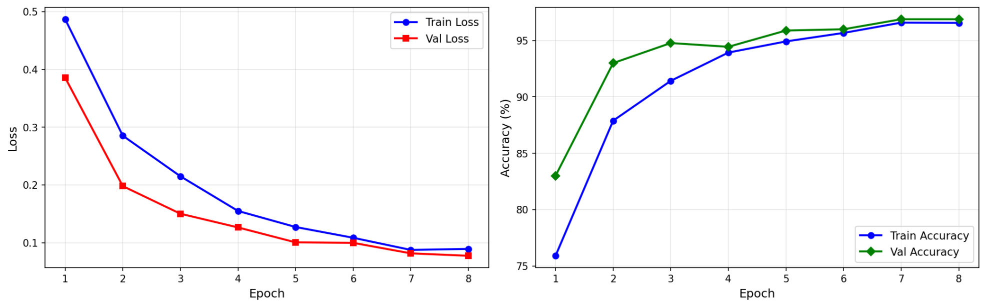

(**b**) Fold 2

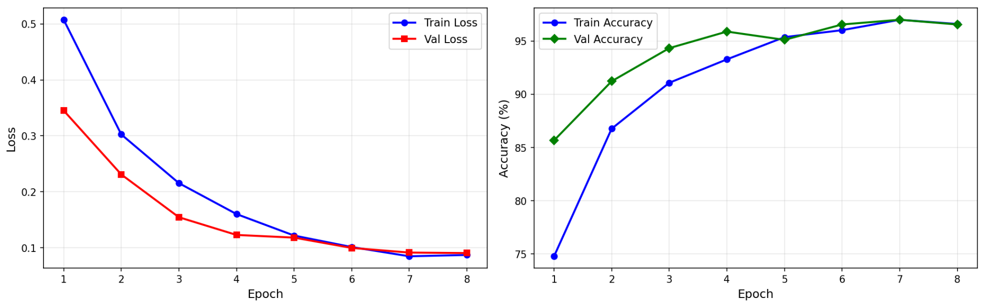

(**c**) Fold 3

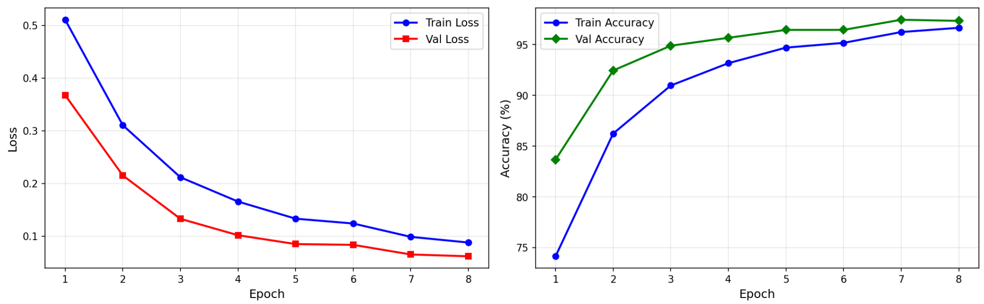

(**d**) Fold 4

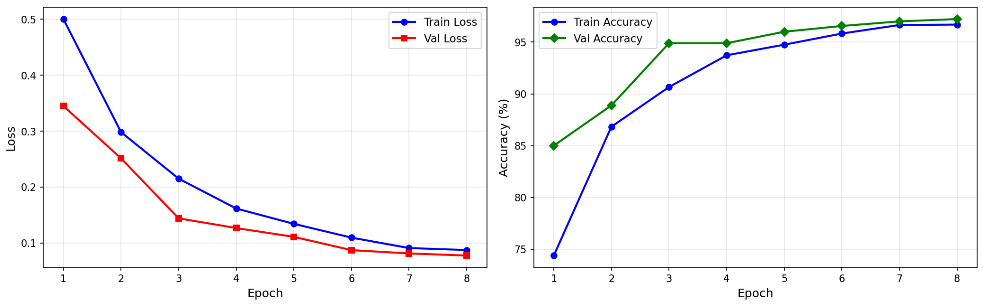

(**e**) Fold 5

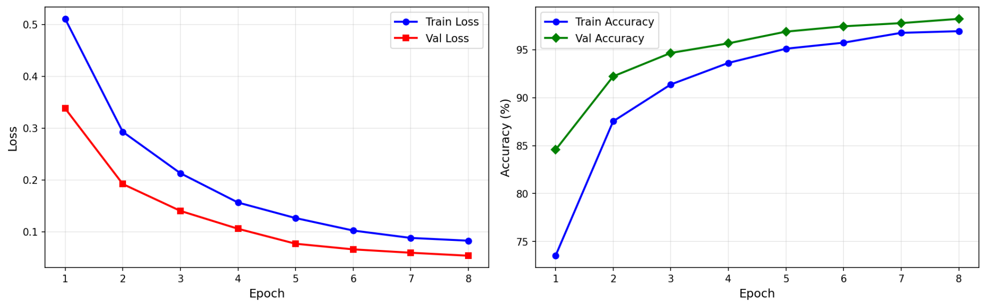

(**f**) Fold 6

Figure S14. Per-fold training and validation loss and accuracy curves for EfficientNet-B0 with Fusion representation on the SSRI dataset.

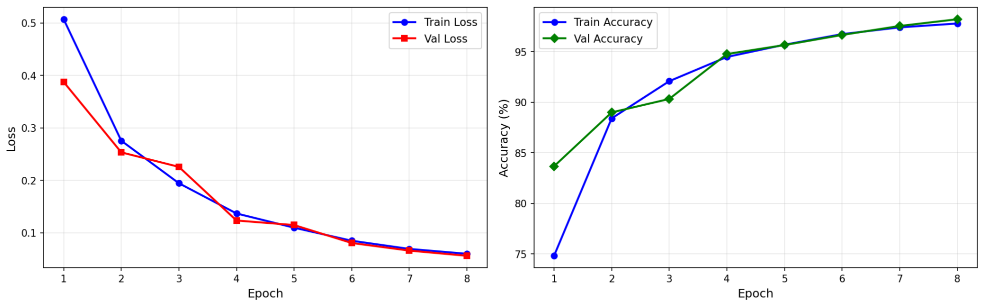

(**a**) Fold 1

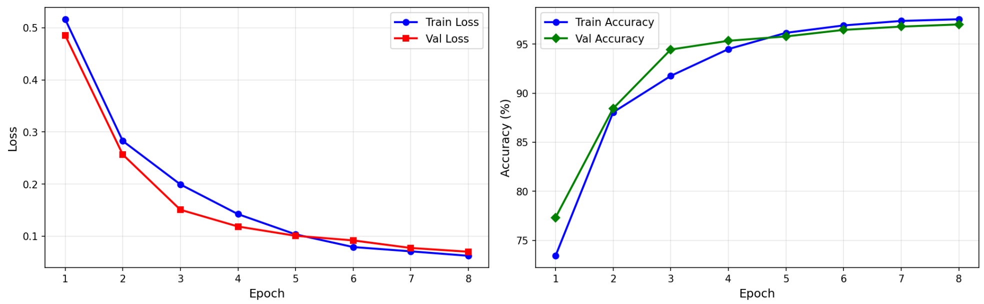

(**b**) Fold 2

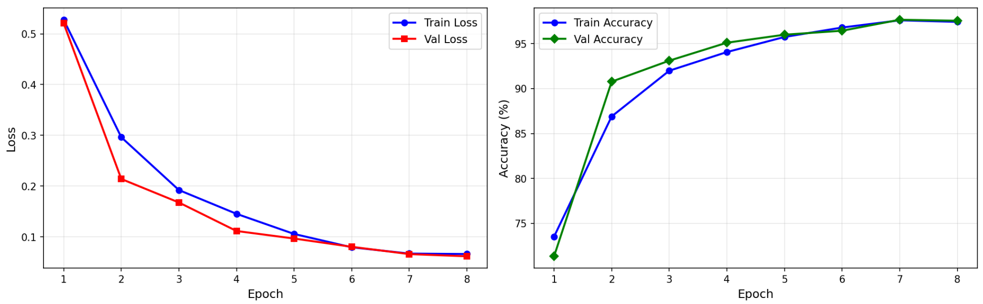

(**c**) Fold 3

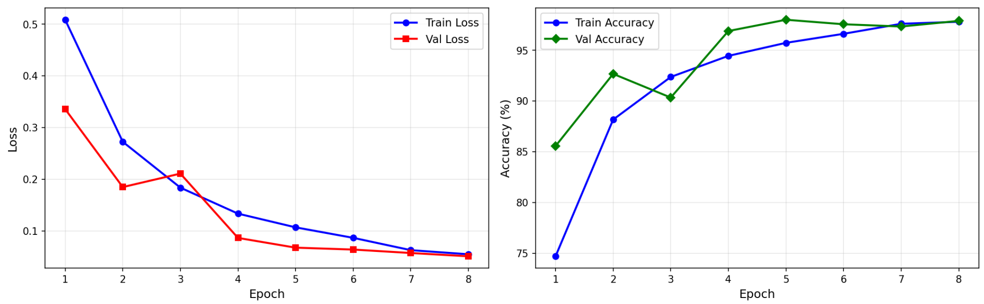

(**d**) Fold 4

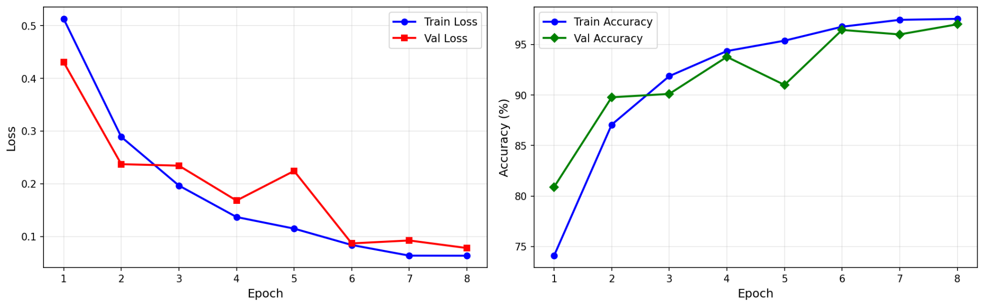

(**e**) Fold 5

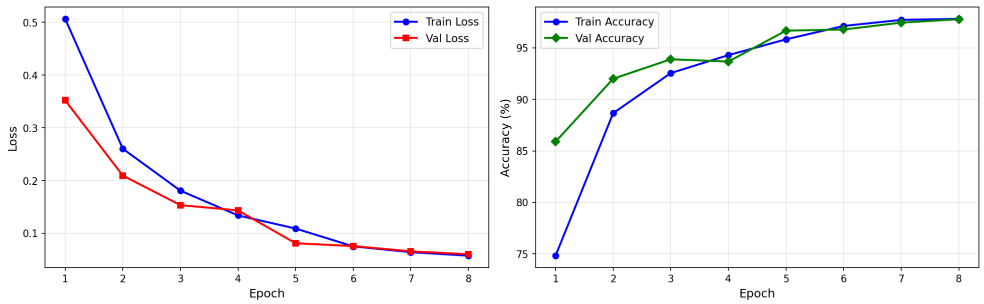

(**f**) Fold 6

Figure S15. Per-fold training and validation loss and accuracy curves for MobileNet-V3 with Fusion representation on the SSRI dataset.

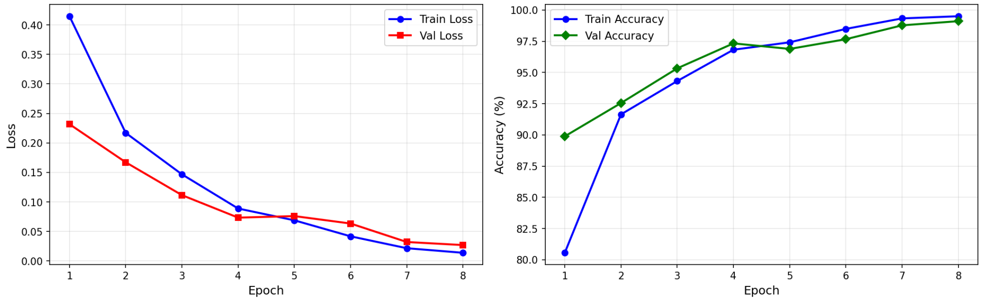

(**a**) Fold 1

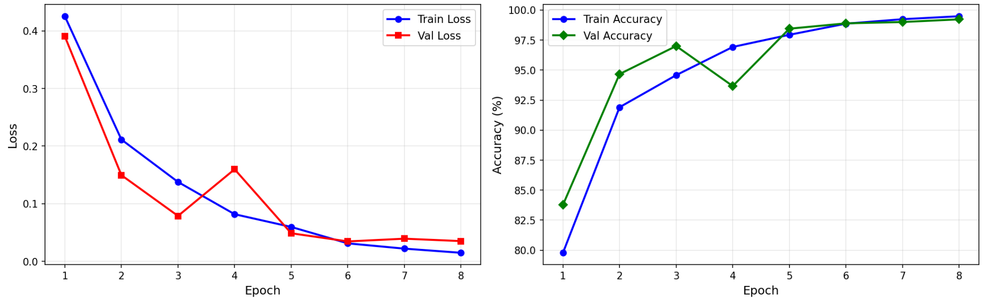

(**b**) Fold 2

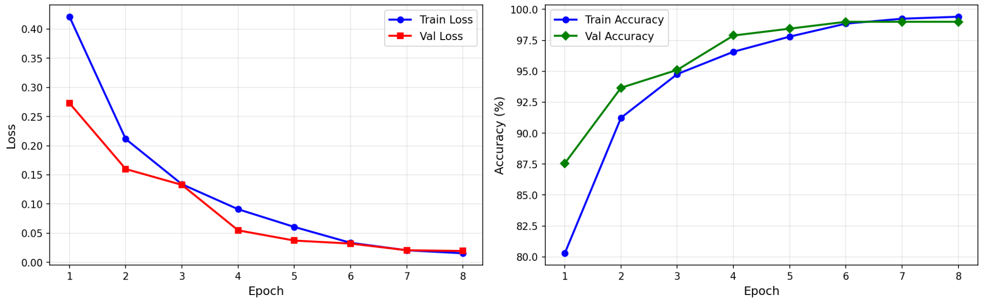

(**c**) Fold 3

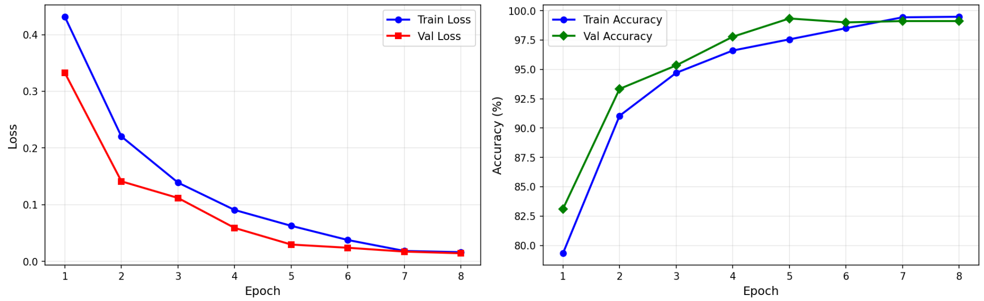

(**d**) Fold 4

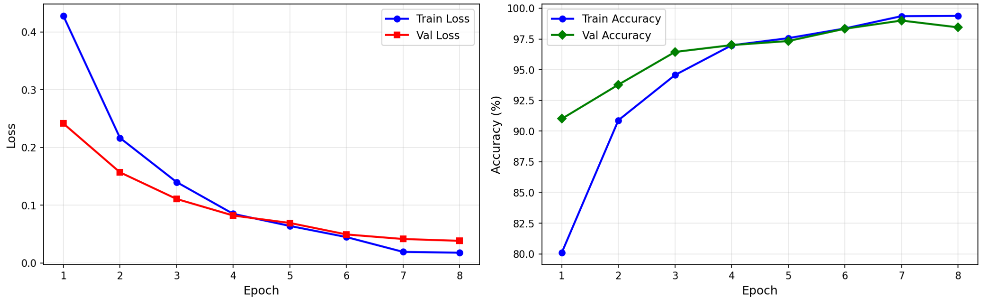

(**e**) Fold 5

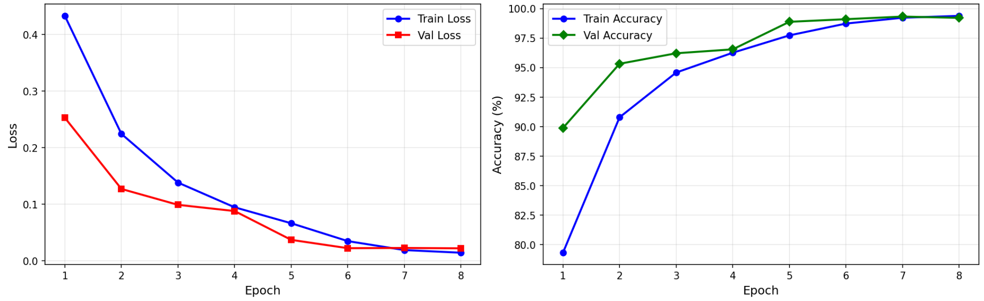

(**f**) Fold 6

Figure S16. Per-fold training and validation loss and accuracy curves for TinyViT-Hybrid with Fusion representation on the SSRI dataset.

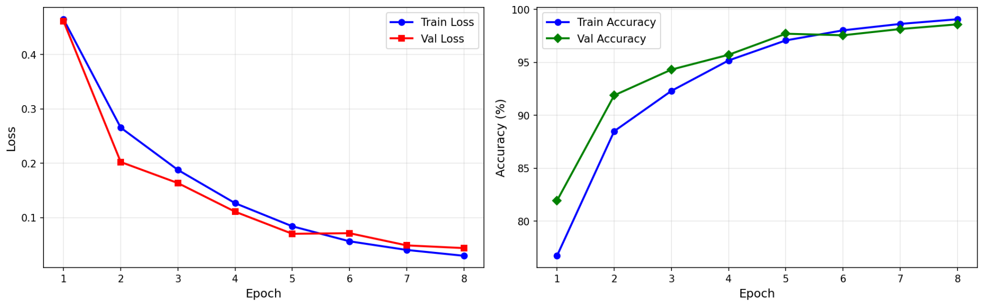

(**a**) Fold 1

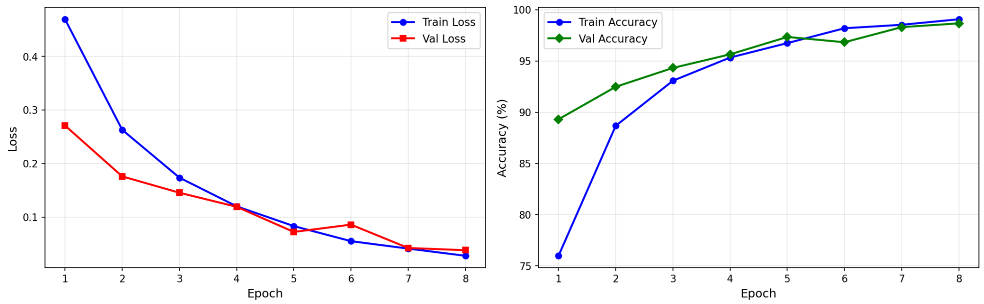

(**b**) Fold 2

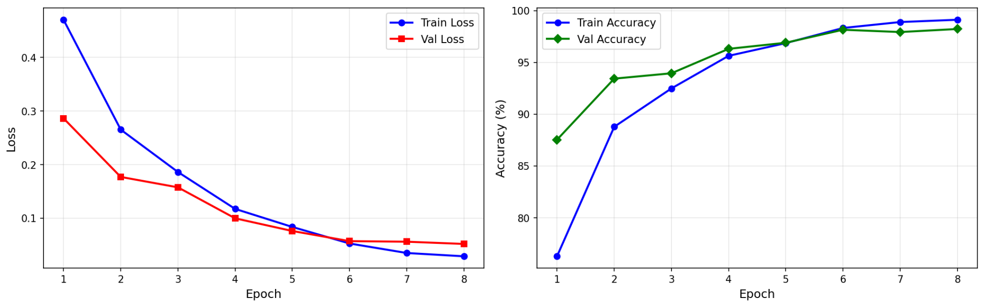

(**c**) Fold 3

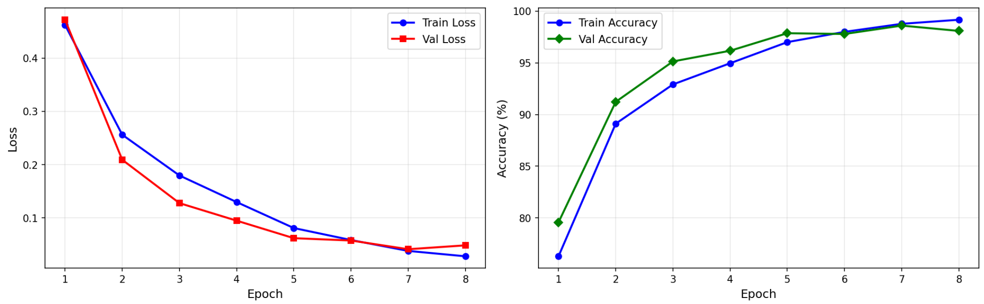

(**d**) Fold 4

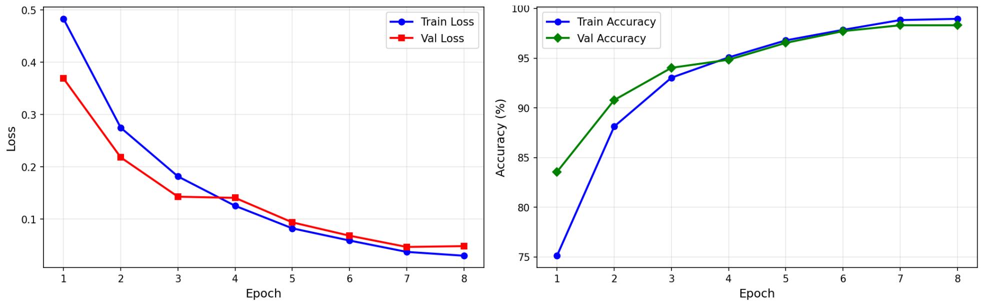

(**e**) Fold 5

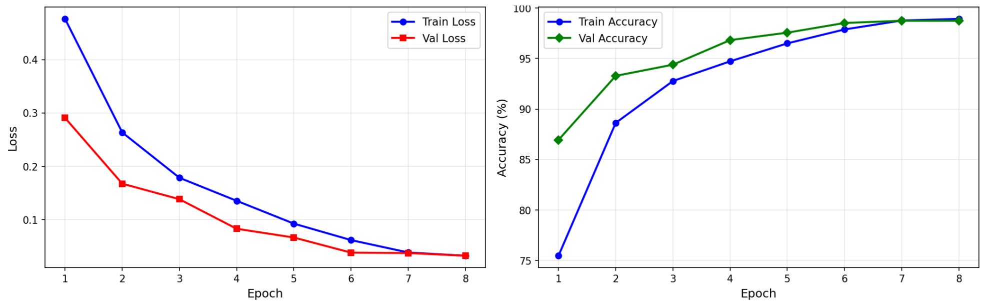

(**f**) Fold 6

Figure S17. Per-fold training and validation loss and accuracy curves for ResNet-18 with CWT representation on the rTMS dataset.

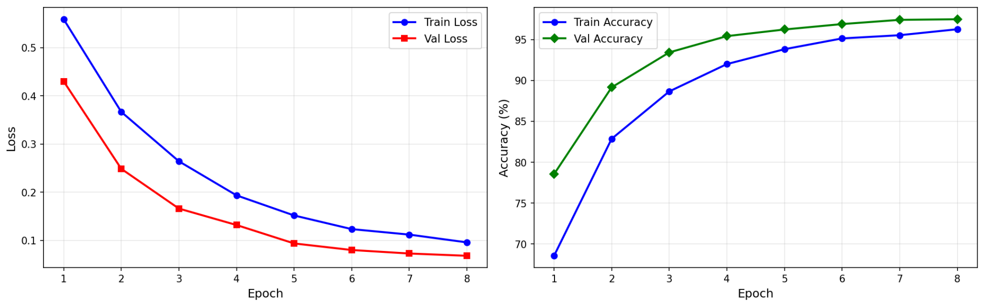

(**a**) Fold 1

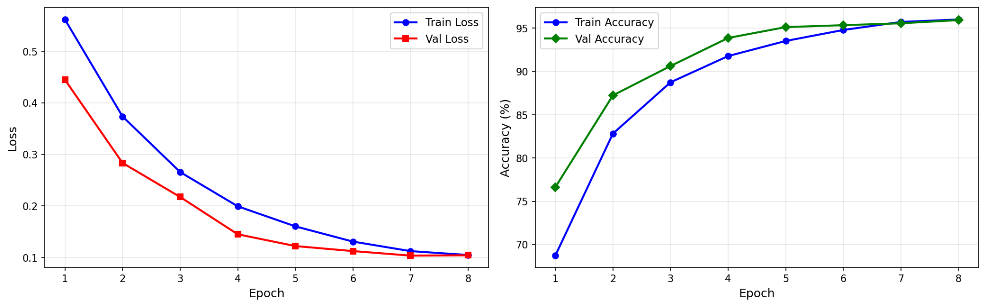

(**b**) Fold 2

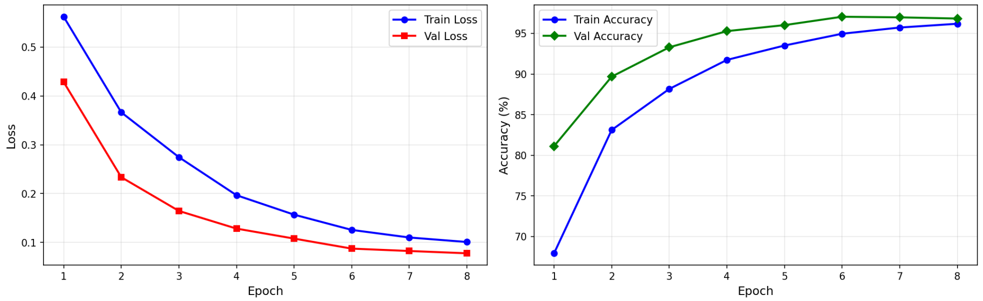

(**c**) Fold 3

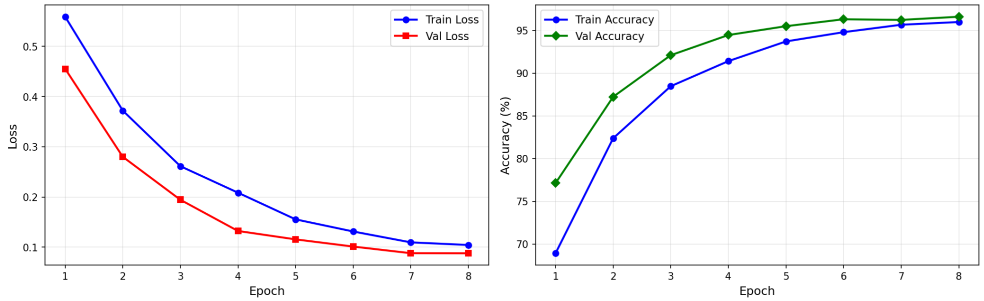

(**d**) Fold 4

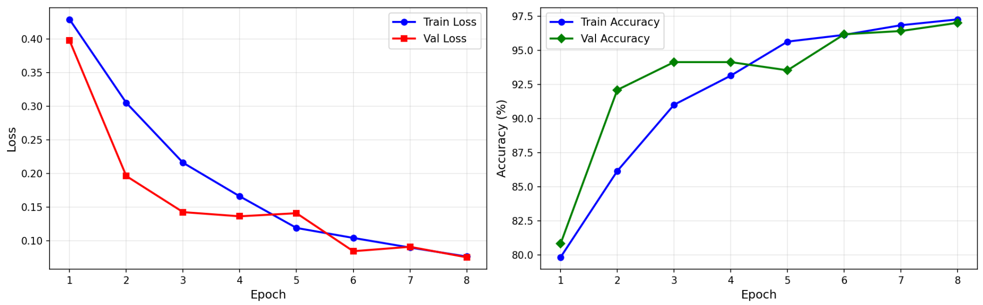

(**e**) Fold 5

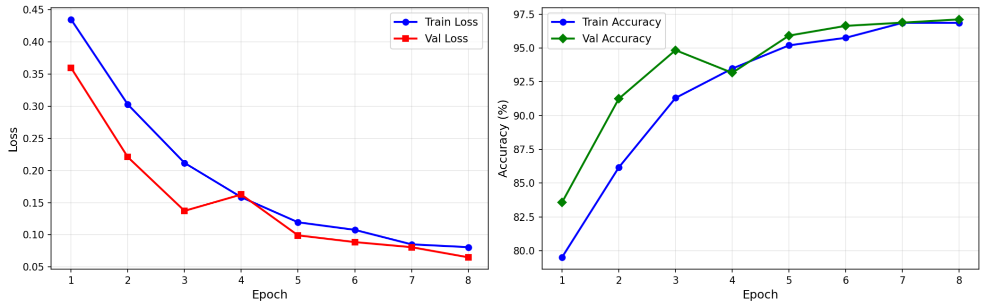

(**f**) Fold 6

Figure S18. Per-fold training and validation loss and accuracy curves for EfficientNet-B0 with CWT representation on the rTMS dataset.

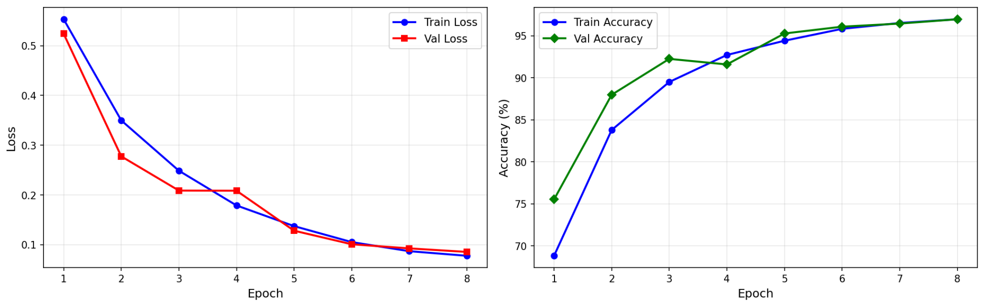

(**a**) Fold 1

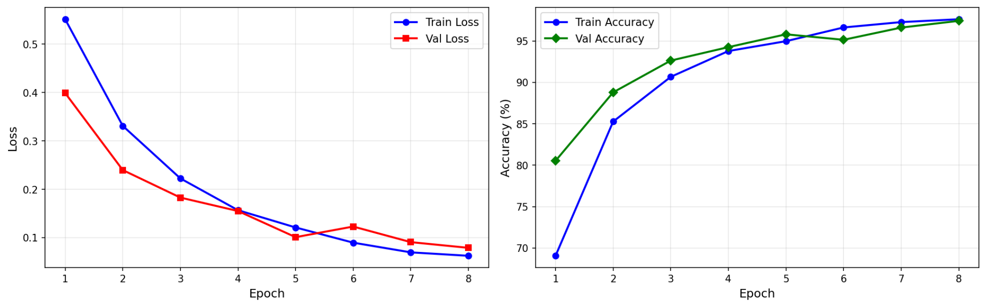

(**b**) Fold 2

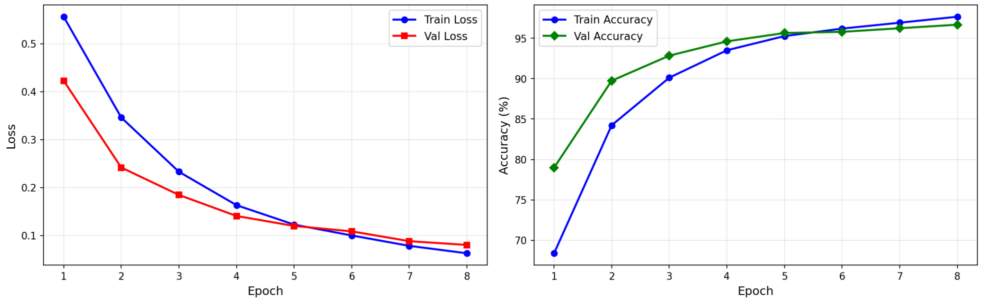

(**c**) Fold 3

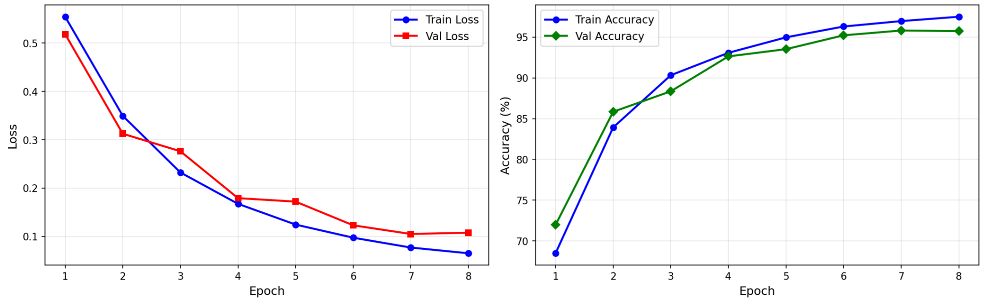

(**d**) Fold 4

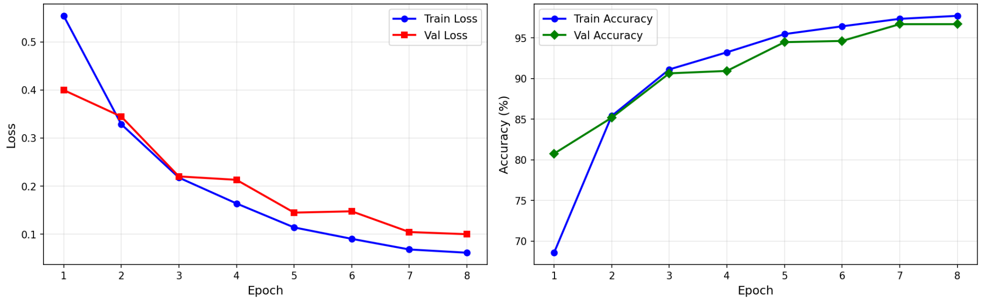

(**e**) Fold 5

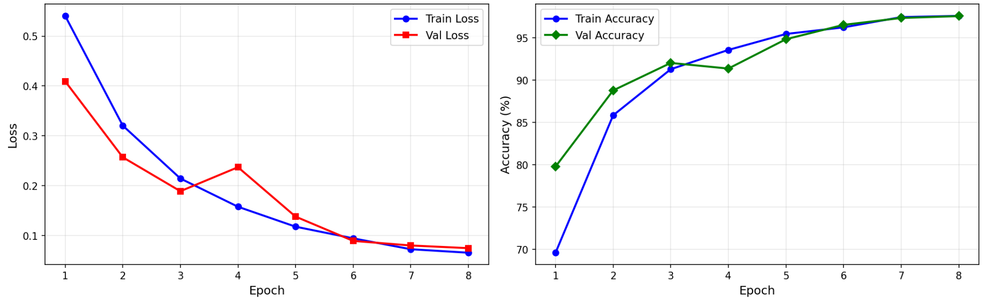

(**f**) Fold 6

Figure S19. Per-fold training and validation loss and accuracy curves for MobileNet-V3 with CWT representation on the rTMS dataset.

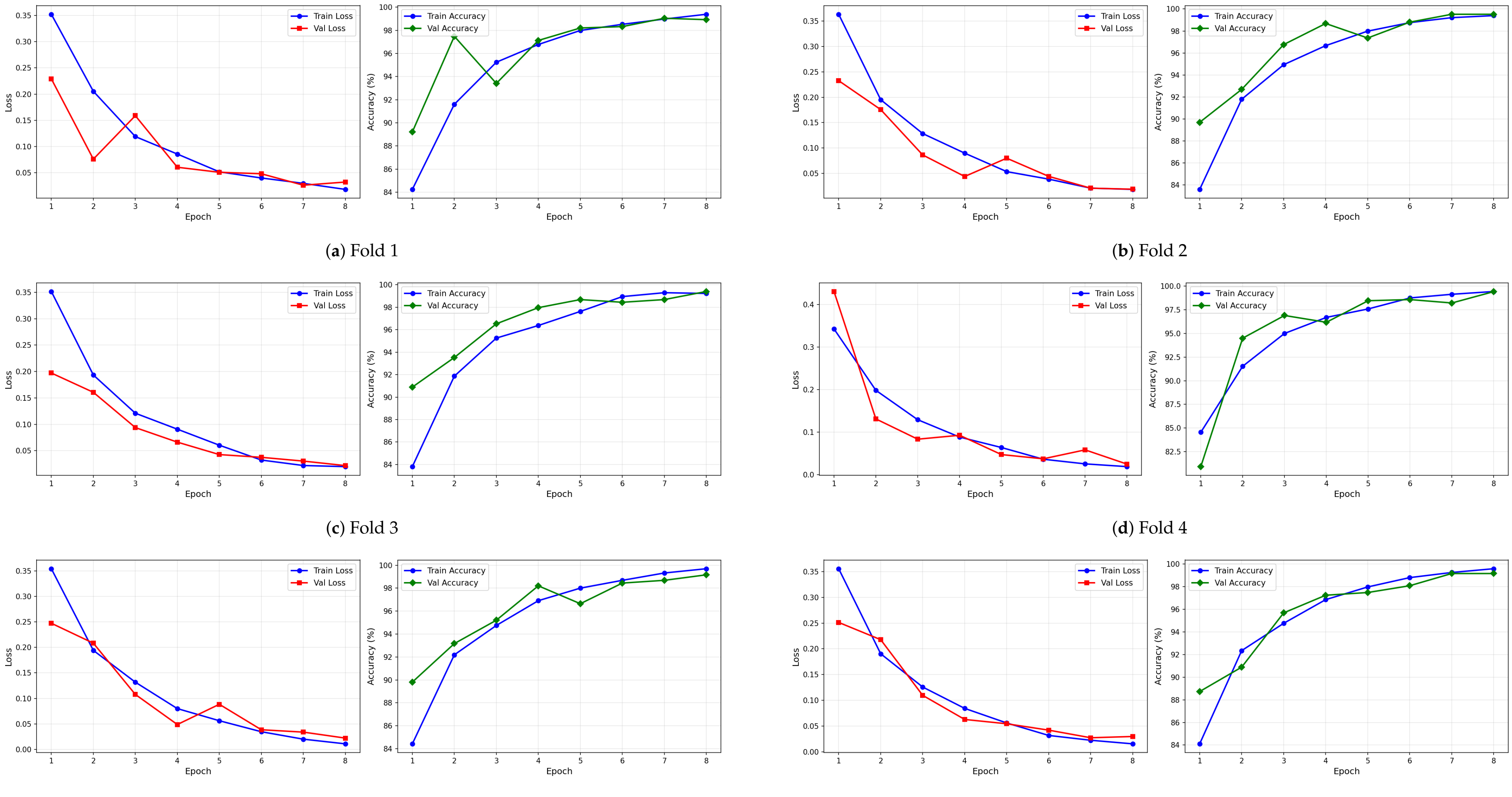

(**a**) Fold 1 (**b**) Fold 2

(**c**) Fold 3 (**d**) Fold 4

(**e**) Fold 5 (**f**) Fold 6

Figure S20. Per-fold training and validation loss and accuracy curves for TinyViT-Hybrid with CWT representation on the rTMS dataset.

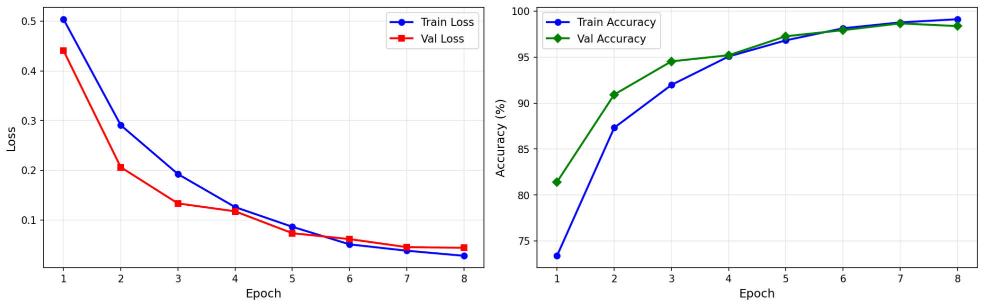

(**a**) Fold 1

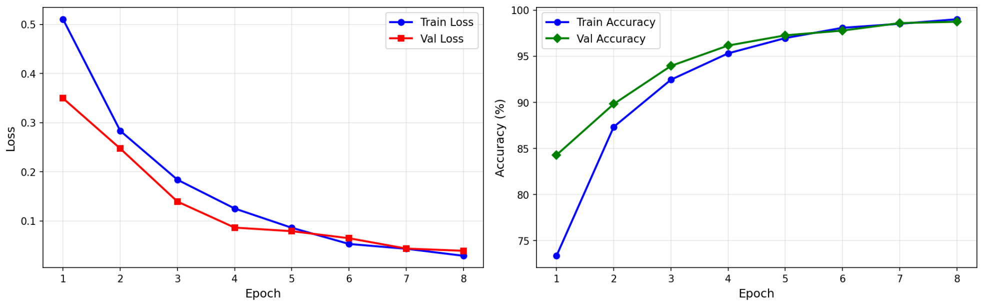

(**b**) Fold 2

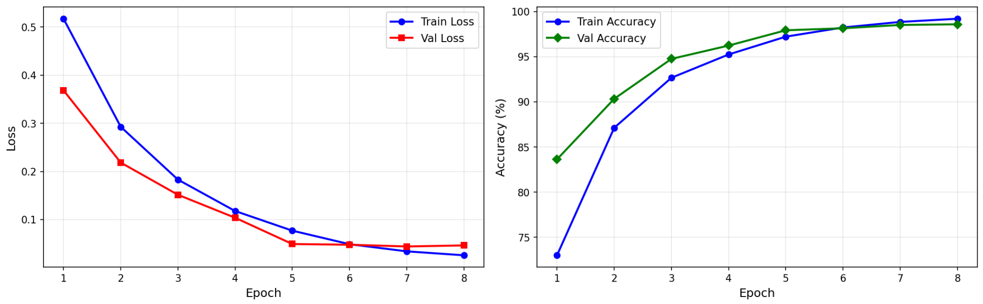

(**c**) Fold 3

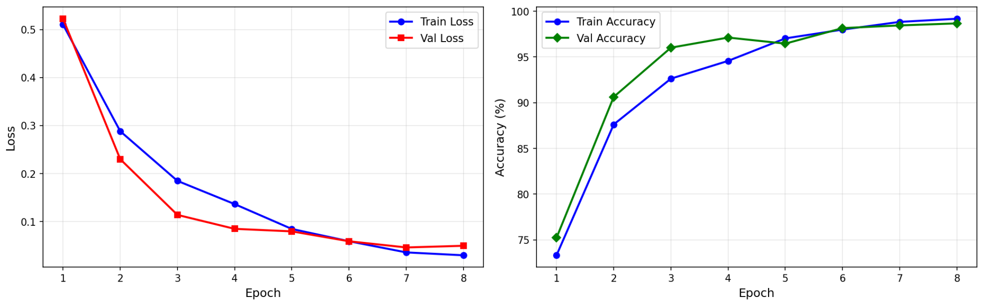

(**d**) Fold 4

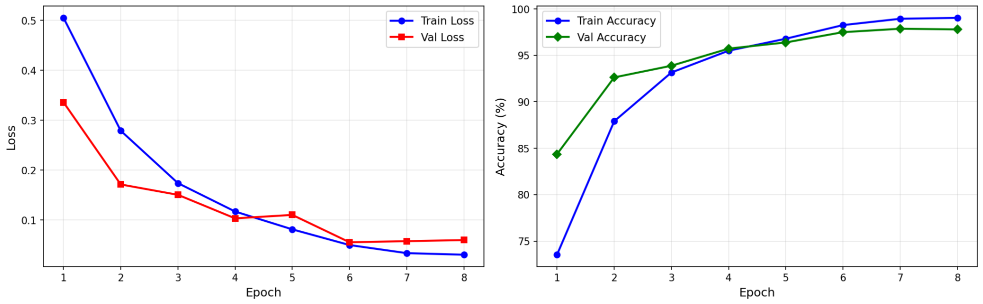

(**e**) Fold 5

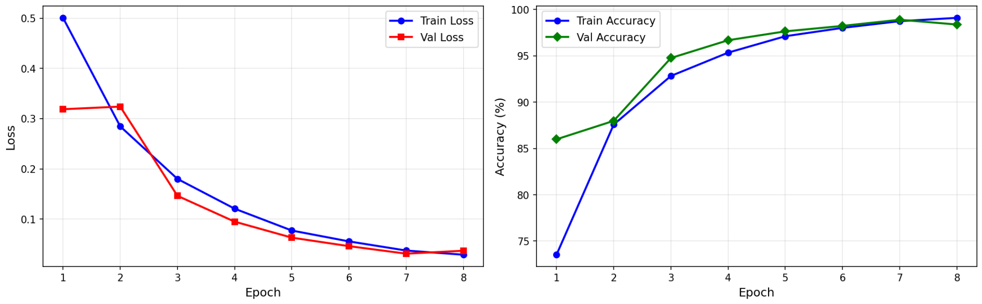

(**f**) Fold 6

Figure S21. Per-fold training and validation loss and accuracy curves for ResNet-18 with VMD representation on the rTMS dataset.

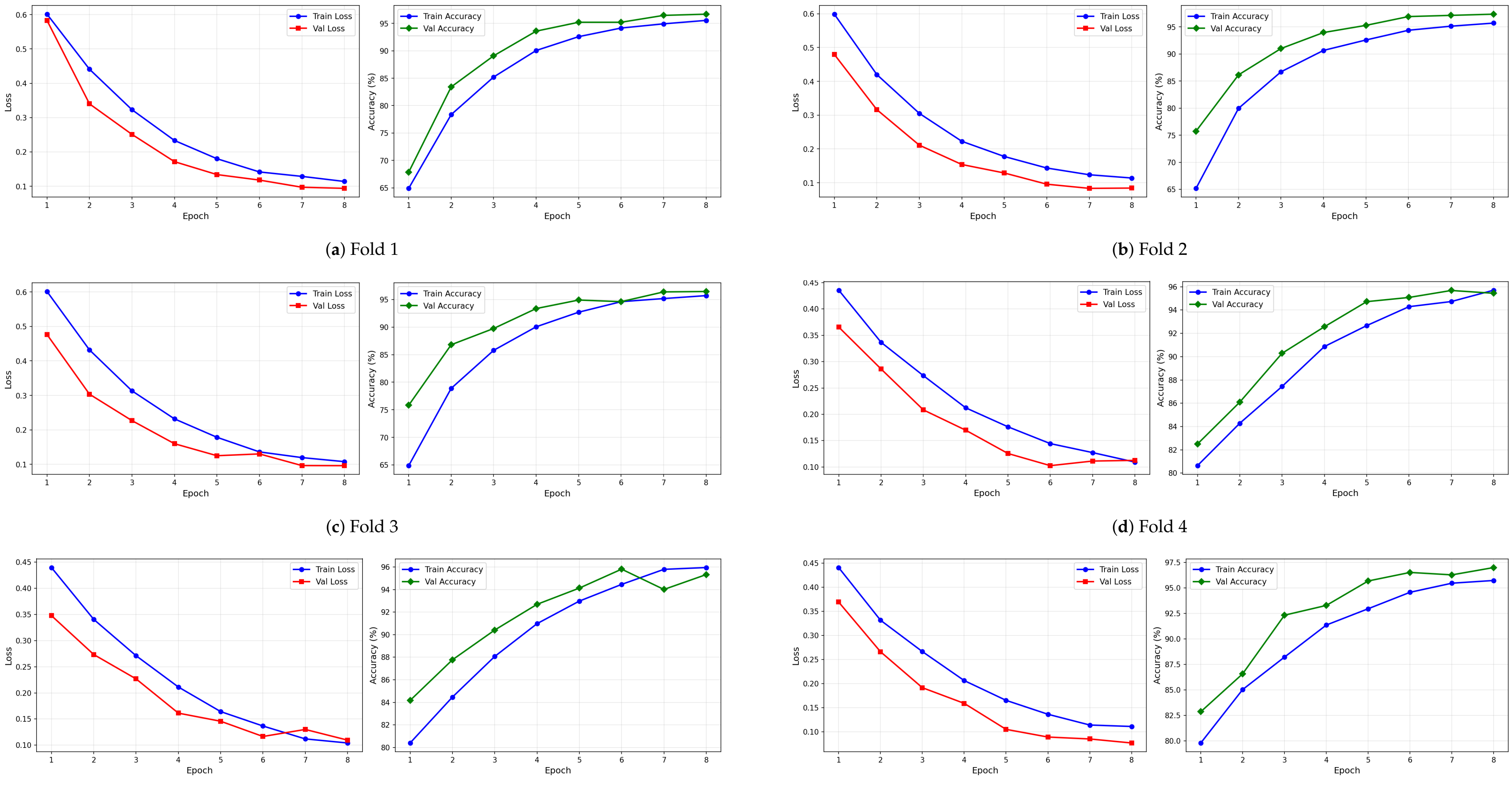

(**a**) Fold 1 (**b**) Fold 2

(**c**) Fold 3 (**d**) Fold 4

(**e**) Fold 5 (**f**) Fold 6

Figure S22. Per-fold training and validation loss and accuracy curves for EfficientNet-B0 with VMD representation on the rTMS dataset.

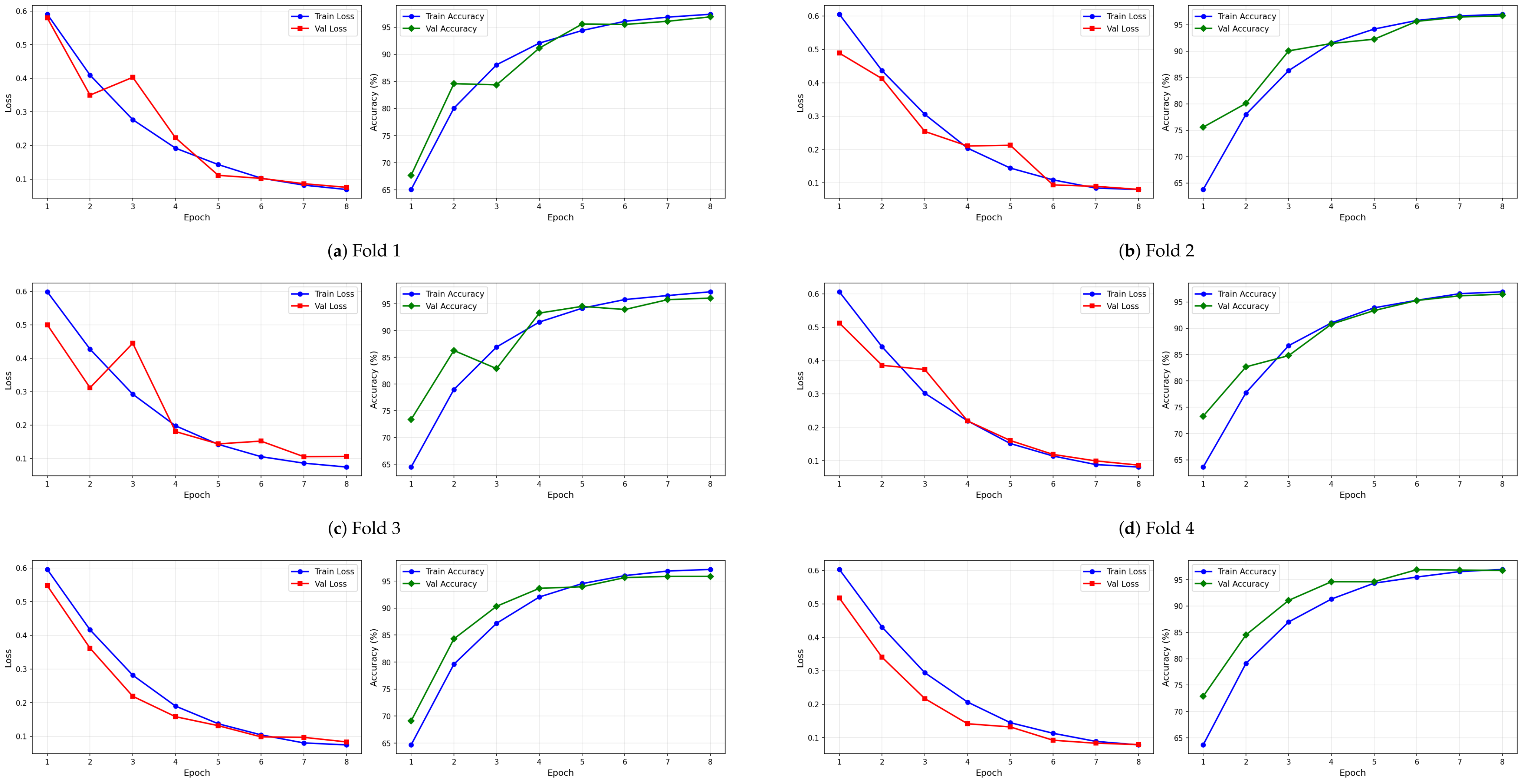

(**a**) Fold 1 (**b**) Fold 2

(**c**) Fold 3 (**d**) Fold 4

(**e**) Fold 5 (**f**) Fold 6

Figure S23. Per-fold training and validation loss and accuracy curves for MobileNet-V3 with VMD representation on the rTMS dataset.

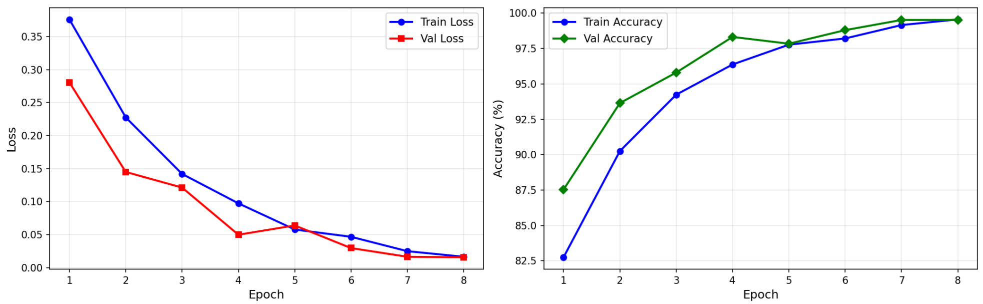

(**a**) Fold 1

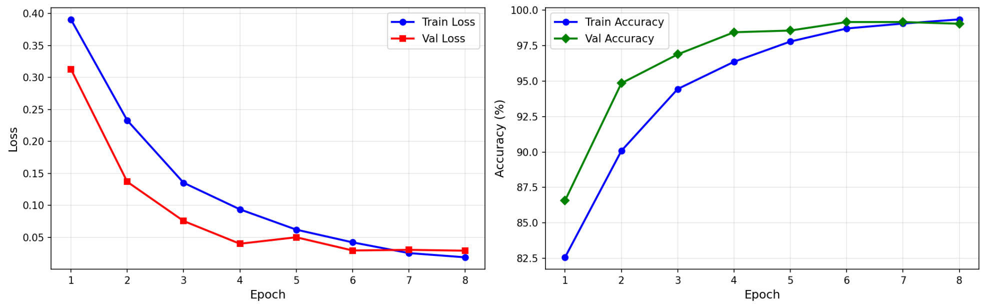

(**b**) Fold 2

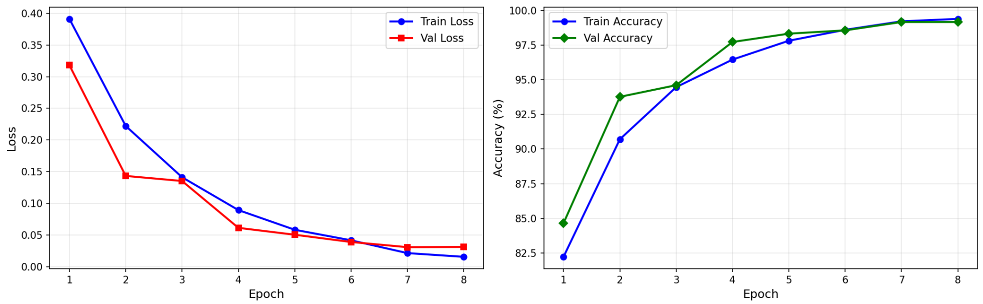

(**c**) Fold 3

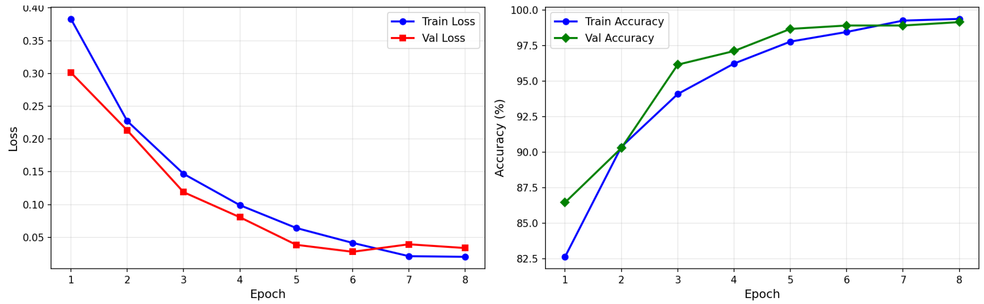

(**d**) Fold 4

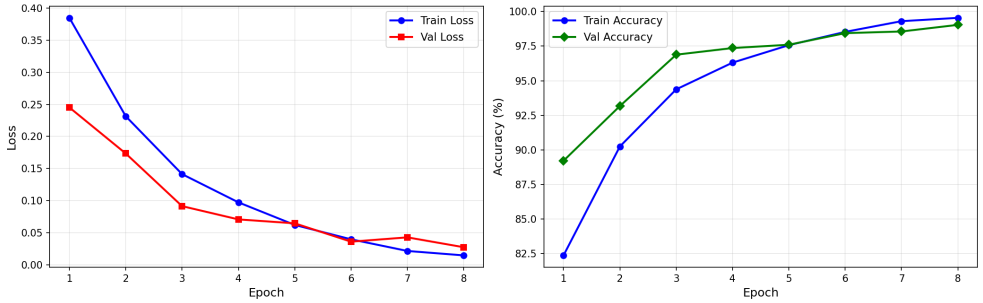

(**e**) Fold 5

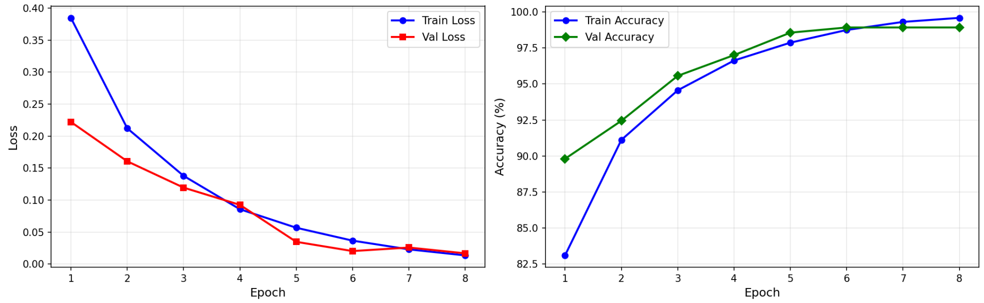

(**f**) Fold 6

Figure S24. Per-fold training and validation loss and accuracy curves for TinyViT-Hybrid with VMD representation on the rTMS dataset.

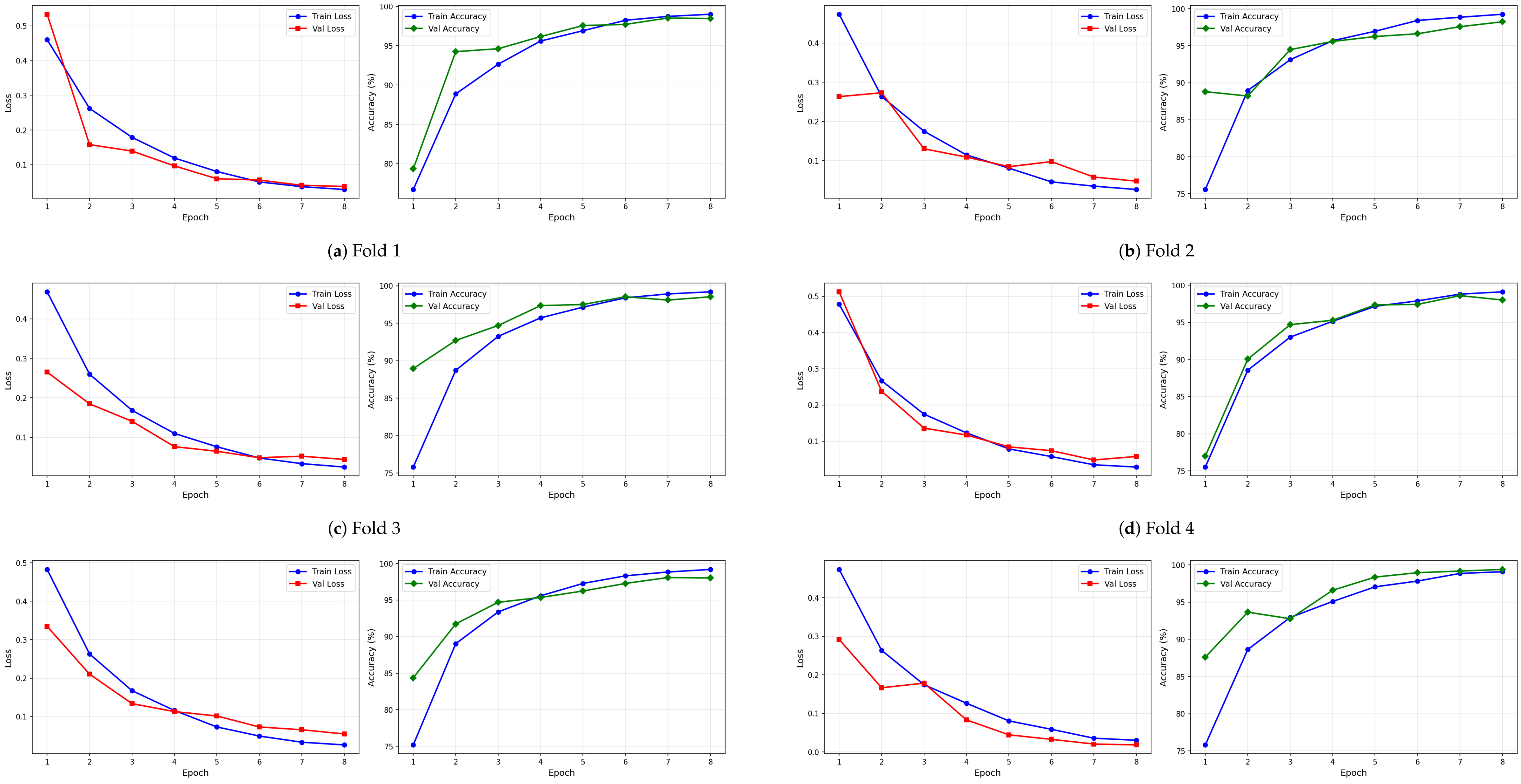

(**a**) Fold 1
(**b**) Fold 2
(**c**) Fold 3
(**d**) Fold 4
(**e**) Fold 5
(**f**) Fold 6

Figure S25. Per-fold training and validation loss and accuracy curves for ResNet-18 with Fusion representation on the rTMS dataset.

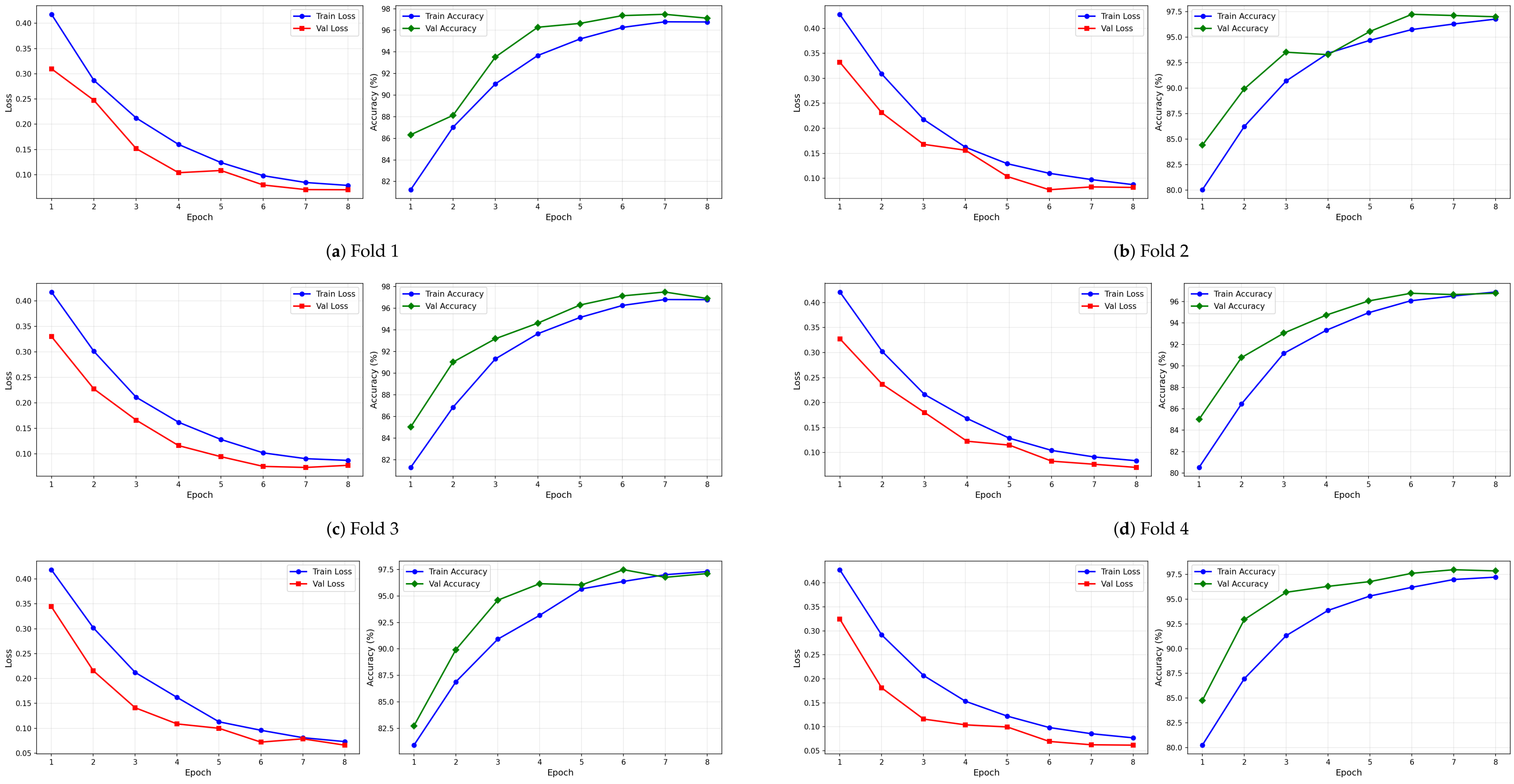

(**a**) Fold 1 (**b**) Fold 2

(**c**) Fold 3 (**d**) Fold 4

(**e**) Fold 5 (**f**) Fold 6

Figure S26. Per-fold training and validation loss and accuracy curves for EfficientNet-B0 with Fusion representation on the rTMS dataset.

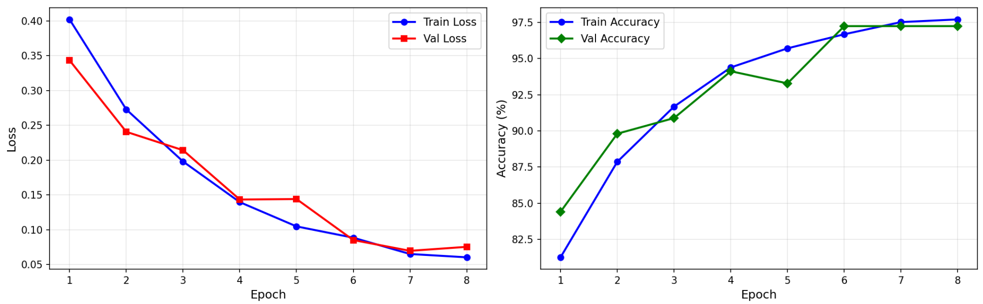

(**a**) Fold 1

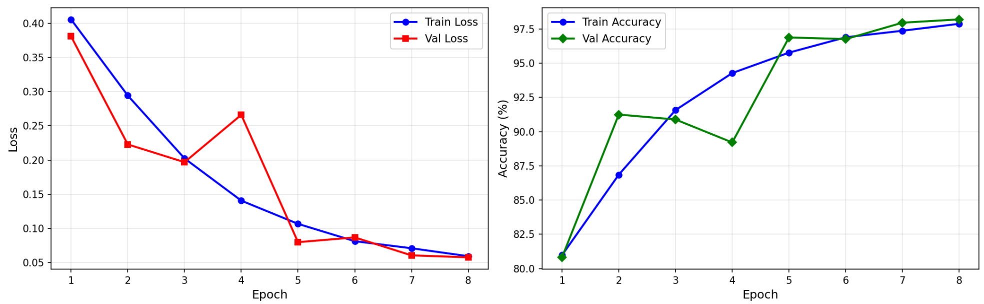

(**b**) Fold 2

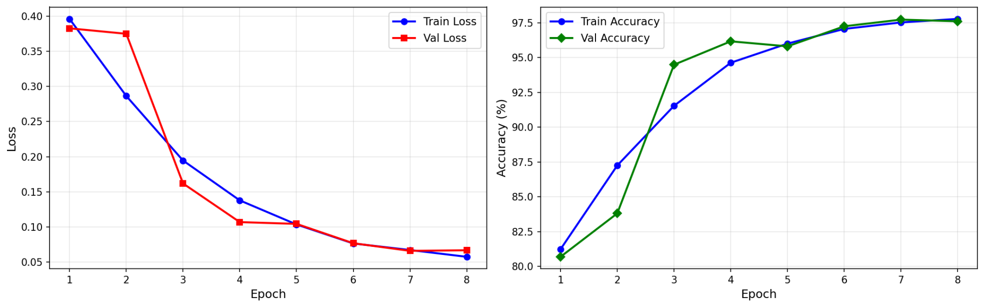

(**c**) Fold 3

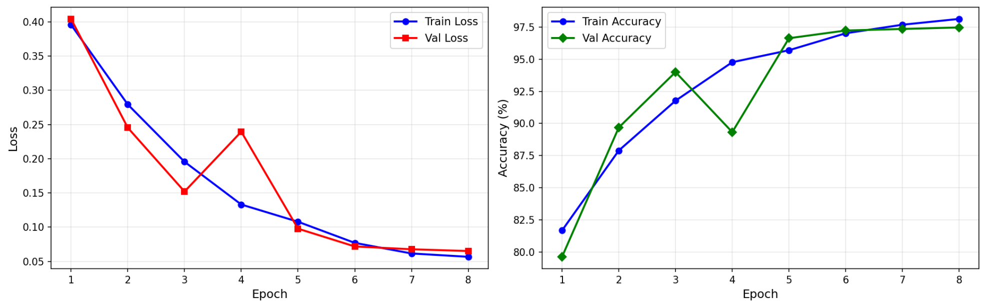

(**d**) Fold 4

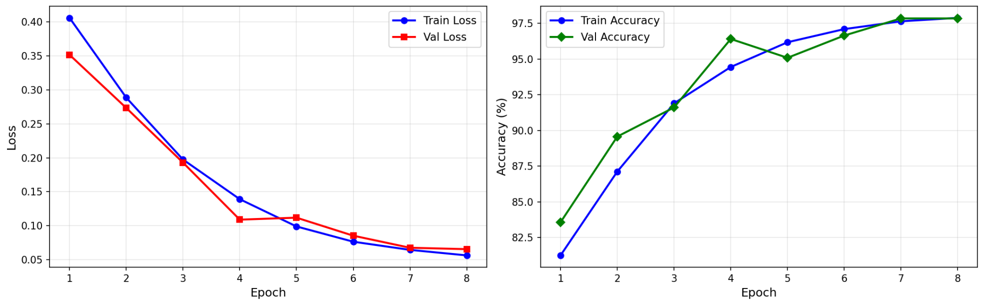

(**e**) Fold 5

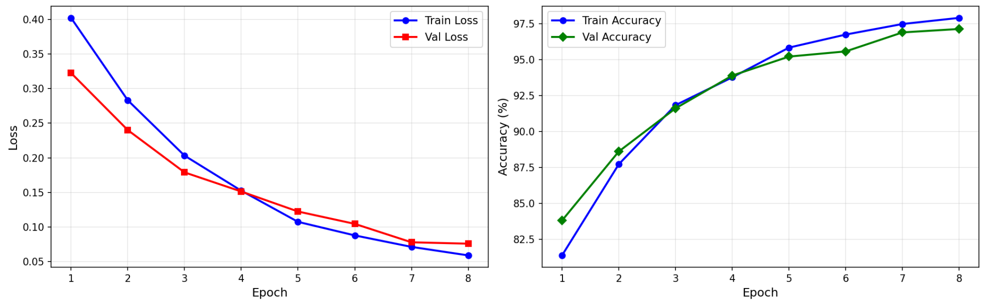

(**f**) Fold 6

Figure S27. Per-fold training and validation loss and accuracy curves for MobileNet-V3 with Fusion representation on the rTMS dataset.

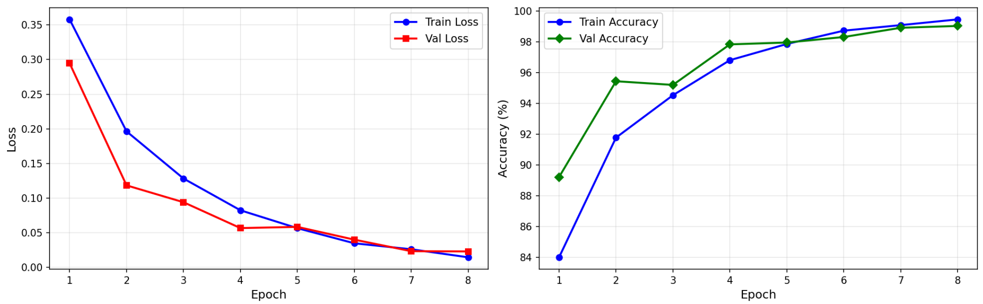

(**a**) Fold 1

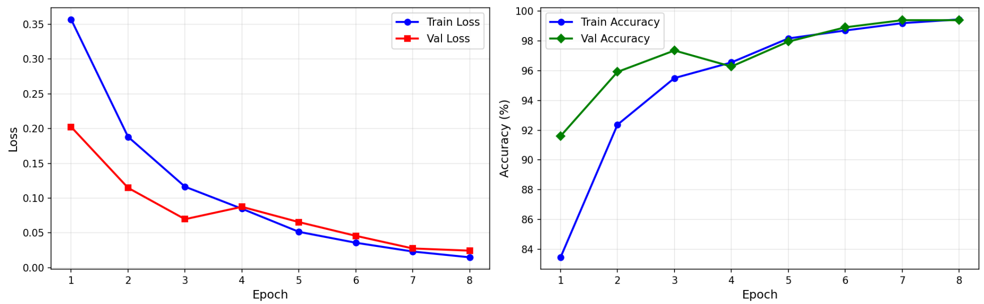

(**b**) Fold 2

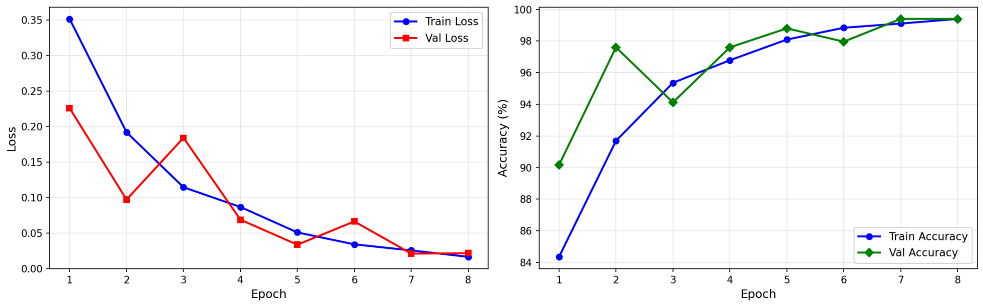

(**c**) Fold 3

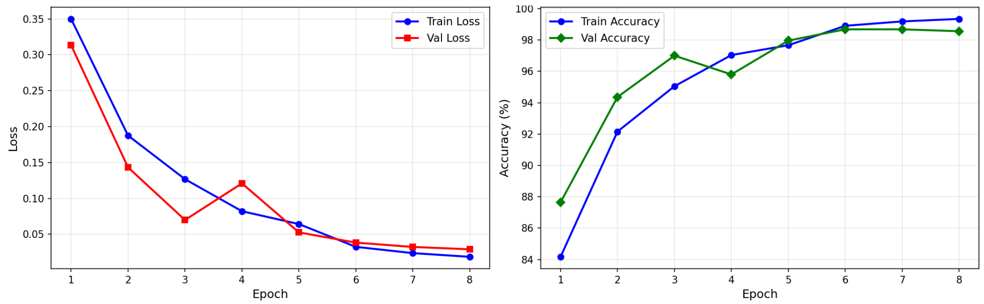

(**d**) Fold 4

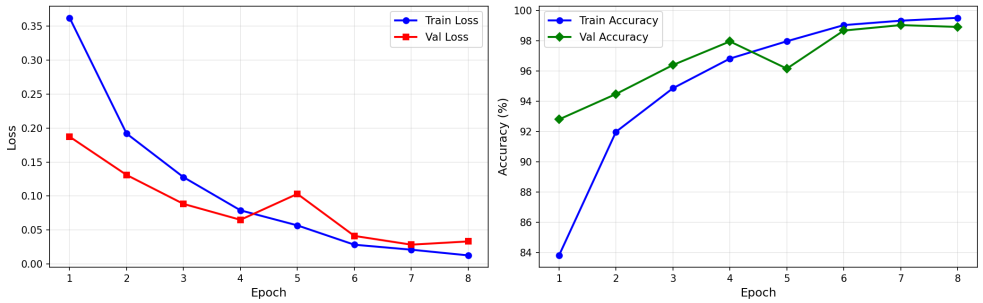

(**e**) Fold 5

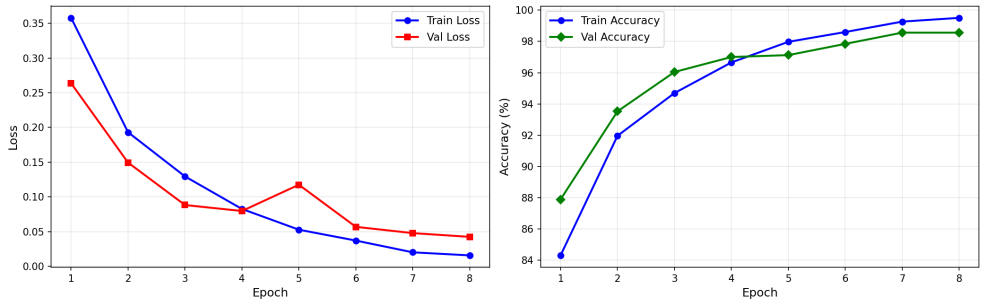

(**f**) Fold 6

Figure S28. Per-fold training and validation loss and accuracy curves for TinyViT-Hybrid with Fusion representation on the rTMS dataset.
